# Pharmacological Approaches to Attenuate Inflammation and Obesity with Natural Products Formulations by Regulating the Associated Promoting Molecular Signaling Pathways

**DOI:** 10.1155/2021/2521273

**Published:** 2021-11-12

**Authors:** Muhammad Imran Khan, Muhammad Zubair Khan, Jin Hyuk Shin, Tia Sun Shin, Young Bok Lee, Min Yung Kim, Jong Deog Kim

**Affiliations:** ^1^Department of Biotechnology, Chonnam Notational University, San96-1, Dun-Duk Dong, Yeosu, Chonnam, (59626), Republic of Korea; ^2^Department of Food Science and Nutrition, Chonnam National University, 77 Yongbong-ro, Buk-gu, Gwangju (61186), Republic of Korea; ^3^Research Center on Anti-Obesity and Health Care, Chonnam National University, San96-1, Dun-Duk Dong, Yeosu, Chonnam, (59626), Republic of Korea; ^4^Department of Refrigeration Engineering, Chonnam National University, San 96-1, Dun-Duk Dong, Yeosu, Chonnam (59626), Republic of Korea

## Abstract

Obesity is a public health problem characterized by increased body weight due to abnormal adipose tissue expansion. Bioactive compound consumption from the diet or intake of dietary supplements is one of the possible ways to control obesity. Natural products with adipogenesis-regulating potential act as obesity treatments. We evaluated the synergistic antiangiogenesis, antiadipogenic and antilipogenic efficacy of standardized rebaudioside A, sativoside, and theasaponin E1 formulations (RASE1) *in vitro* in human umbilical vein endothelial cells (HUVECs), 3T3-L1 preadipocytes respectively, and *in vivo* using a high-fat and carbohydrate diet-induced obesity mouse model. Orlistat was used as a positive control, while untreated cells and animals were normal controls (NCs). Adipose tissue, liver, and blood were analyzed after dissection. Extracted stevia compounds and green tea seed saponin E1 exhibited pronounced antiobesity effects when combined. RASE1 inhibited HUVEC proliferation and tube formation by suppressing VEGFR2, NF-*κ*B, PIK3, and-catenin beta-1 expression levels. RASE1 inhibited 3T3-L1 adipocyte differentiation and lipid accumulation by downregulating adipogenesis- and lipogenesis-promoting genes. RASE1 oral administration reduced mouse body and body fat pad weight and blood cholesterol, TG, ALT, AST, glucose, insulin, and adipokine levels. RASE1 suppressed adipogenic and lipid metabolism gene expression in mouse adipose and liver tissues and enhanced AMP-activated protein kinase levels in liver and adipose tissues and in serum adiponectin. RASE1 suppressed the NF-*κ*B pathway and proinflammatory cytokines IL-10, IL-6, and TNF-*α* levels in mice which involve inflammation and progression of obesity. The overall results indicate RASE1 is a potential therapeutic formulation and functional food for treating or preventing obesity and inflammation.

## 1. Introduction

Obesity is defined as having a body mass index of ≥30 kg/m^2^ and is a major and complex metabolic disorder characterized by high body fat accumulation. Obesity is associated with type II diabetes mellitus, hypertension, coronary heart disease, fatty liver disease, and different types of cancer [1, 2]. Expansion of adipose tissue due to an imbalance between energy intake and expenditure results in obesity. This excess energy is stored in adipose cells, thereby enlarging or increasing cell numbers (hypertrophy and hyperplasia, respectively) [3]. With over 2.1 billion cases being reported, obesity remains a continuous global epidemic accounting for approximately 5% of all deaths [4–6]. Obesity has been reported to lead to cognitive dysfunction, depression, and emotional trauma, resulting in a poor quality of life [4, 7].

In adipose tissue, adipokines, including monocyte chemoattractant protein-1, tumor necrosis factor-alpha (TNF-*α*), and interleukin 6 (IL-6), play major roles in inflammation in type 2 diabetes, insulin resistance, cardiovascular diseases, and cancer [8, 9]. Macrophages associated with adipocytes in adipose tissues play role in physiological energy metabolism and homeostasis however in chronic energy surplus conditions macrophages develop chronic inflammation and contribute to the expansion and progression of the adipose tissues by secreting the proinflammatory cytokines IL-1, IL-6, and TNF alpha [10, 11]. Hence, the molecular mechanisms underlying adipocyte differentiation, physiology, and morphology must be investigated to contribute to the overall understanding of adiposity and to develop new ways to combat inflammation, obesity, and associated complications. Information regarding the role of genes, proteins, and signaling intermediates regulating adipogenesis is also of importance, with all these factors serving as promising targets for the discovery of novel antiobesity drugs. Molecular mechanisms of obesity mediated by cytokines, adiponectin, and leptin have been correlated with increased inflammation and oxidative stress, leading to metabolic disease development. Lipid metabolism has been targeted for the treatment of obesity and obesity-related metabolic diseases [12].

Adipogenesis is a complex mechanism of adipocyte differentiation from preadipocytes and involves various transcription factors, including peroxisome proliferator-activated receptor gamma (PPAR*γ*), CCAAT enhancer-binding protein alpha (C/EBP*α*), and the enzyme fatty acid synthase (FAS) [13]. Adipocytes store excess energy as total triglycerides (TGs), which are produced by lipogenic enzymes. This process is regulated by TG synthesis factors, including lysophosphatidic acid acyltransferase theta, diacylglycerol acyltransferase 1, and phosphatidate phosphatase [14].

AMP-activated protein kinase (AMPK) is a central regulator of energy metabolism and mitochondrial biogenesis in adipocytes [15]. AMPK activation deactivates acetyl-CoA carboxylase (ACC), resulting in decreased fatty acid synthesis and increased fatty acid oxidation (FAO) [16]. ACC promotes intracellular lipid synthesis in late-stage adipogenesis. Thus, regulation of adipogenic transcription factors is important for attenuating adipocyte differentiation. Additionally, AMPK activation elevates the mitochondrial oxidative capacity and the expression of factors involved in thermogenesis, including PRDM16, PGC1*α*, and UCP1 [17, 18]. Recent studies suggest that AMPK is necessary for brown adipose tissue (BAT) development in obese mice, which is associated with PRDM16 expression. Additionally, PGC1*α* is induced not only by PRDM16 but also by AMPK activation [19, 20]. Consequently, researchers have been exploring plant materials containing antioxidants as alternatives to previously adopted conventional approaches, including surgery and antiobesity drugs that lack enduring effects, have side effects [21], or have a yo-yo effect [22]. Therefore, identifying natural products with antioxidant properties for the treatment and/or prevention of obesity and its comorbidities would be insightful.

An unhealthy diet is one of the major risk factors for obesity and diabetes, with dietary fat intake widely accepted as being directly or indirectly related to obesity, diabetes, high cholesterol, and other diseases [23]. Adipogenesis involves several complex signaling pathways and is considered a crucial target for obesity control, as it contributes to the pathophysiology of obesity and related complications. Natural products with adipogenesis-regulating potential act as therapeutics that are safe to use for the treatment of obesity.

Several bioactive compounds, such as isoflavonoids, anthocyanins, glucosinolates, alkaloids, phenolic acids of Asteraceae, coumarins, coumestans, curcuminoids, tannins, glucosinolates, isothiocyanates, secoiridoids, and flavonols, inhibit adipocyte differentiation (adipogenesis), as evidenced by studies on various signaling molecules, transcriptional factors, and genes [24, 25].

Bioactive compound consumption from the diet or intake of dietary supplements is one of the possible ways to control obesity, thus, preventing or reducing the risk of developing various obesity-related diseases. Saponins and phenolics from food legumes possess biological activities, including anti-inflammatory [24], anticancer [25], and antihypertension activities [26]. Adzuki bean, mainly produced and consumed in China and other East Asian countries, has been used as a traditional Chinese herbal medicine and food for thousands of years. Its effects include antitumor, antidiabetic [27], and antioxidant activities [28].

Additionally, the anti-inflammatory [29], antiarthritic [30], antibacterial [31], antiangiogenic [32], antioxidative [33], antiviral [34], and neuroprotective effects [35] of green tea and its constituents have frequently been examined. The hypolipidemic and antiobesity effects of green tea in animals and humans have slowly become a hot topic for molecular nutrition and food research. Over the past decade, studies have shed light on the role of green tea catechins in controlling hyperlipidemia and fat mass gain in high-fat diet-induced obese rodent models. However, little is known regarding the exact antiobesity effects of green tea in humans, and the underlying mechanism of body weight management, especially regarding the regulation pathway.

*Stevia rebaudiana* (SR) is often used in the food industry owing to its steviol glycoside content, a suitable calorie-free sweetener. Furthermore, both *in vitro* and *in vivo* studies indicate that these glycosides and SR extracts have pharmacological and therapeutic properties, including antioxidant, antimicrobial, antihypertensive, antidiabetic, and anticancer properties. However, there is no evidence that they offer weight-loss advantages over other nonnutritive sweeteners [36]. It has been reported that Stevia leaf extract possesses antiadipogenic effects in high-fat diet-induced obese mice. However, the pure compounds responsible for such effects have not been identified [37].

## 2. Results

### 2.1. Identification, Structure Elucidation, and Quantification of the Isolated Compounds

**Figure 1:**
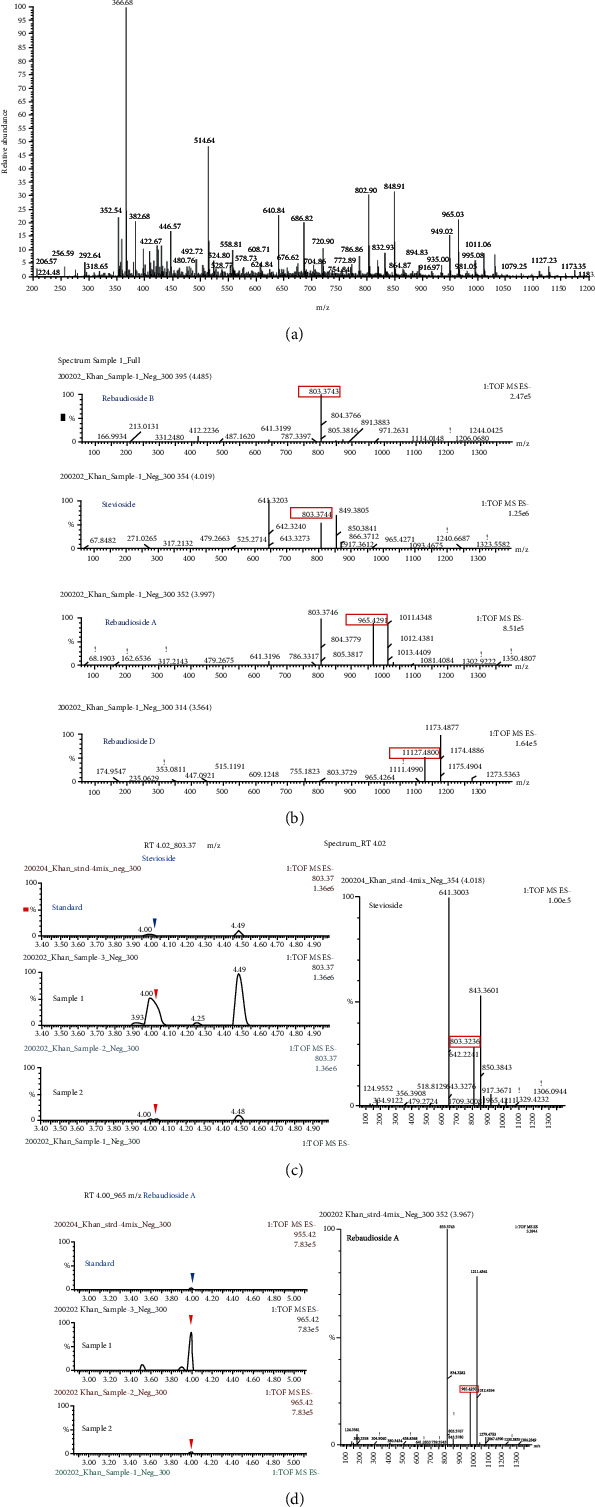
Isolated *Stevia rebaudiana* fraction of pure compounds detected and determined by LC-MS-QTOF. (a) LC-MS chromatogram of isolated stevia fraction of pure compounds. (b) Identification of pure compounds present in the fraction by comparison with standards. (c) LC-MS chromatogram and mass spectrum of isolated pure compound stevioside. (d) LC-MS chromatogram and mass spectrum of isolated pure compound rebaudioside A.

The whole process of compounds isolation is given in Supplementary File 1. Purified fractions isolated from *S. rebaudiana* ethanolic extracts by resin column chromatography followed by preparative HPLC were analyzed by LC/TOF-MS and NMR for identification, structure elucidation, and quantification of the compounds present (Figure 1; Supplementary File 2 & File 3).

**Figure 2:**
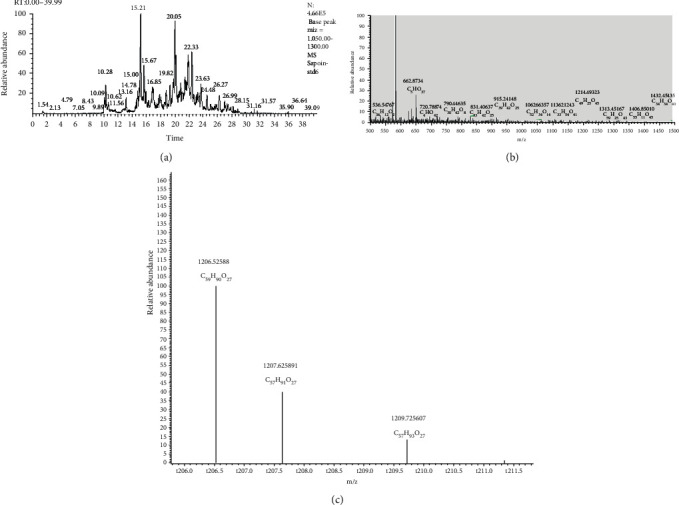
LC-MS-TOF analysis of green tea seed extracted saponins. (a) LC-MS chromatogram of green tea seed extracted saponin-rich fraction. (b) LC-MS spectrum of green tea seed extracted with pure saponins. (c) Base peak intensity mass spectrum of green tea seed isolated saponin theasaponin E1 fraction.

Steviosidea, rebaudioside A, rebaudioside B, and rebaudioside D were found in the isolated fraction. The fraction was resubjected to HPLC, and a pure stevioside and rebaudioside A fraction with traces of rebaudioside B and D was obtained. Theasaponin E1 was isolated by HPLC from the purified green tea seed extract saponin-rich fraction. All identification, quantification, and determination data of LC/TOF-MS and NMR are provided in the supplementary materials (Supplementary File 4 & File 5, respectively). LC/TOF-MS data of green tea seed saponins are presented in Figure 2.

### 2.2. Cell Viability

**Figure 3:**
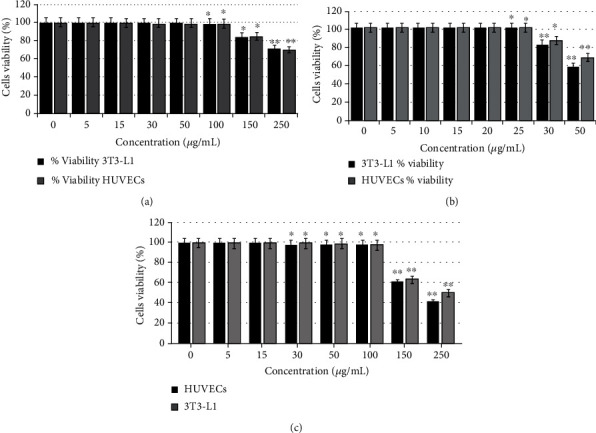
The cell viability and toxicity of pure saponins, extracted stevia compounds, and their combination (RASE1) in HUVECs and 3T3-L1 cells via MTT assay. (a) Cell (HUVECs and 3T3) viability under various concentrations of extracted stevia compounds. (b) Cell (HUVECs and 3T3) viability under various concentrations of theasaponin E1. (c) Cell (HUVECs and 3T3) viability under various concentrations of RASE1. Cell viability was calculated for each group by determining the absorbance of the wells after the addition of the MTT reagent. Safe and toxic concentrations were determined. Data are expressed as mean ± SEM (*n* = 3). (^∗^*p* < 0.01 and ^∗∗^*p* < 0.001 compared to the control).

The toxicity of pure compounds of the isolated stevia fraction, green tea seed saponin, E1, stevia fraction, and E1 (RASE1) combination in HUVECs and 3T3-L1 cells was determined by MTT assay. Safe and toxic doses were determined from cell viability measured by MTT assay. Cell viability results of the test samples are shown in Figure 3, which also shows that the purified stevia fraction of stevioside and rebaudiosides and the combined RASE1 sample did not pose toxic effects to HUVEC and 3T3-L1 cell viability up to the working concentration range of 5–100 *μ*g/mL. Theasaponin E1 alone was safe at the dose rate of 5–25 *μ*g/mL.

### 2.3. Antiangiogenesis Effect

**Figure 4:**
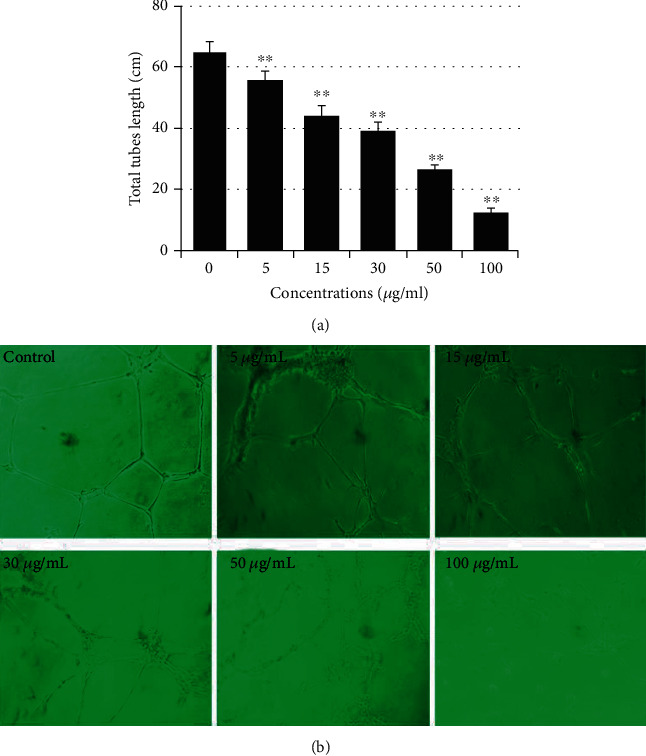
Inhibitory effects of RASE1 on HUVEC proliferation and capillary tube formation in Matrigel. Cells proliferated in EBM2 media in the presence and absence of various concentrations of RASE1, i.e., 5, 30, 50, and 100 *μ*g/mL (nontoxic range) of samples and tube formation by HUVECs in Matrigel was observed using a phase contrast inverted microscope (Nikon, Tokyo, Japan). Each treatment and control (cells not treated with sample) group was photographed using Scion Image software (NIH, ML, USA) at five random sites. Total tube length was calculated for each group. Experiments were performed in triplicate. (a) Total tube length of HUVECs in control and treated groups. (b) Photographs of HUVEC proliferation and tube formation in the control wells and as treated groups (with various concentrations of RASE1). Data are the mean values ± SEM of three separate experiments (data are significant at ^∗^*p* < 0.01 and ^∗∗^*p* < 0.001 compared to the control).

The effect of isolated stevia extract fraction alone, containing steviosides and rebaudiosides, and the combination of the isolated stevia fraction with green tea seed saponins was examined on endothelial cell tube formation by the quantitative analysis of the tube length formed on the Matrigel. Each purified stevia extract fraction and RASE1 exhibited significant inhibitory effects on capillary tube formation in a dose-dependent manner when HUVECs were supplemented with increasing doses (5, 15, 30, 50, and 100 *μ*g/mL) of test samples. The lowest concentration of samples significantly inhibiting the tubular structure formation was 10 *μ*g/mL, and the complete disruption of capillary tubes was observed at 100 *μ*g/mL. Results demonstrated that the extracted stevia compound and theasaponin E1 effectively inhibited HUVEC proliferation and tube formation on Matrigel. Total tube length was significantly decreased with increasing sample concentration compared to control cell tube length (Figure 4(a)). Inhibitory effects of samples on HUVEC proliferation and capillary tube formation in Matrigel are obvious from the images shown in Figure 4(b), taken from cells of each concentration.

To evaluate the mechanism of antiangiogenic effects of stevia and saponin samples, the effects on VEGFR-2 expression and the PI3K/AKT/ERK pathway were further investigated in HUVECs using safe sample concentration ranges. The regulatory effect on the gene expression level was determined by RT-PCR using gene-specific primers (Table S1 supplementary File 6).

**Figure 5:**
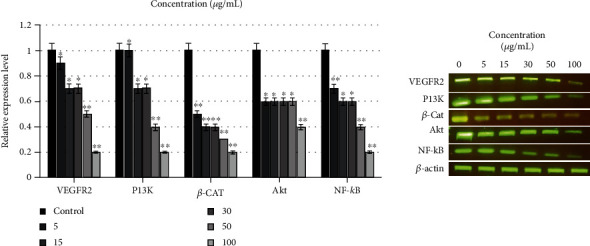
Gene expression levels of various angiogenesis-promoting signaling molecules and transcriptional factors in HUVECs treated with various extracted stevia compounds and tea seed saponin doses. Data are the mean values ± SEM of three separate experiments (data are significant at ^∗^*p* < 0.01 and ^∗∗^*p* < 0.001 compared to the control).

Samples reduced VEGFR-2 and VE-Cadherin complex expression via NF-*κ*B downregulation, an important growth factor for proliferation and vascular remodeling. Various growth factors play important roles in PI3K/AKT and ERK pathway activation. RT-PCR results showed the *AKT* and *ERK* mRNA level inhibition via VEGFR-2 downregulation in a dose-dependent manner. Treatment with RASE1 median concentration (100 *μ*g/mL) led to significant *PI3K*, *AKT*, *ERK*, *VEGFR-2*, NF-*κ*B, and *β*-catenin mRNA expression inhibition. Evidently, the extracted stevia compounds and GTS downregulated PI3K/AKT and ERK pathways by the suppression of VEGFR expression (Figure 5).

### 2.4. Lipid Accumulation Inhibition in 3T3-L1 Preadipocytes

**Figure 6:**
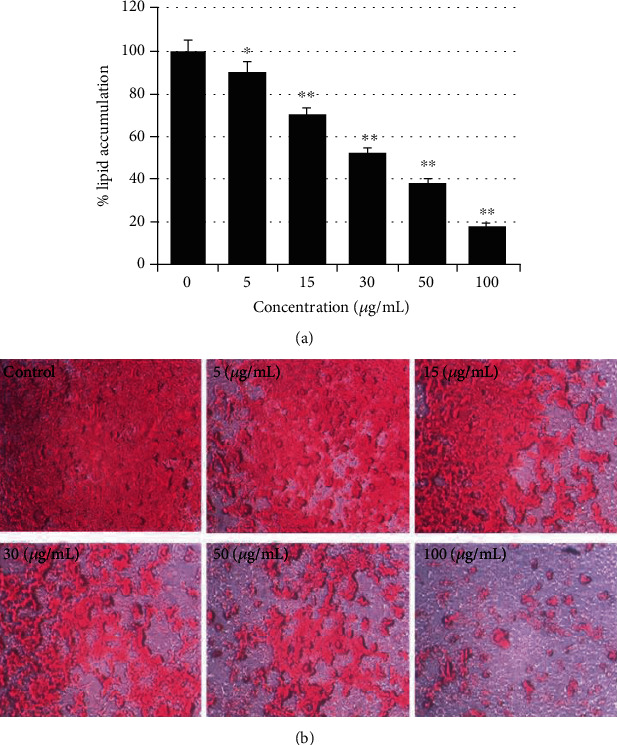
Fat accumulation inhibition in 3T3-L1 via adipogenesis and lipogenesis suppression by RASE1 determined by Oil red O staining. 3T3-L1 premature cells were cultured and proliferated in DMEM under various RASE1 concentrations. (a) Lipid droplets accumulated in mature differentiated 3T3-L1 cells were extracted with Oil red O solvent and quantified by checking the absorbance with a microtiter plate reader spectrophotometer. Data are the means ± SEM of three different experiments. (^∗^*p* < 0.001 and ^∗∗^*p* < 0.001 compared to the control). (b) Lipid droplets accumulated in mature 3T3-L1 cells of control and treated groups were observed using a phase contrast inverted microscope (Nikon, Tokyo, Japan) and photographed with Scion Image software (NIH, ML, USA) at five random sites.

To investigate the effect of the stevia-derived purified compound fraction and adipocyte differentiation, preadipocyte 3T3-L1 cells were differentiated into mature adipocytes in the presence of various sample concentrations. Cell treatment with the stevia-derived fraction and RASE1 during differentiation suppressed lipid accumulation in a dose-dependent manner as measured by Oil red O staining (Figure 6(a)). The TG amount was significantly decreased in the treated group compared with that of the controls. The inhibitory effect of samples on adipogeneses and lipogenesis significantly increased with increasing sample concentrations. Fat accumulation inhibition in 3T3-L1 cells by various samples and control doses is shown in Figure 6(b).

### 2.5. Effects on mRNA Expression Levels of Adipogenesis- and Lipogenesis-Related Genes

**Figure 7:**
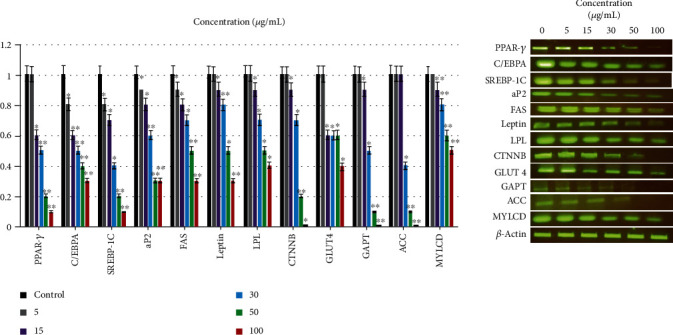
The RASE1 effect on mRNA gene expression levels of adipogenesis- and lipogenesis-related genes and signaling molecules in 3T3-L1 adipocytes. 3T3-L1 preadipocytes were differentiated to adipocytes in the absence (control) and presence of RASE1 concentrations. (a) Relative mRNA expression levels of adipogenesis, lipogenesis, and signaling proteins. mRNA levels of each group were normalized to *β*-actin and expressed relative to the control group. (b) PCR products of adipogenesis- and lipogenesis-related genes and signaling proteins visualised as gel bands. Experiments were performed in triplicate, and data are expressed as the ±SEM of three separate experiments (data are significant at ^∗^*p* < 0.01 and ^∗∗^*p* < 0.001 compared to the control).

Mechanisms of AnT-Fr antihyperlipidimic activity on 3T3-L1 proliferation and lipid accumulation were analyzed by RT-PCR. RT-PCR results showed that AnT-Fr significantly inhibited the expression levels of adipogenesis- and lipogenesis-related genes and signal molecules, including PPAR*γ*, C/EBP*α*, aP2, and sterol regulatory element-binding protein (SREBP-1c), in a dose-dependent manner (Figure 7).

### 2.6. AMPK Activation

**Figure 8:**
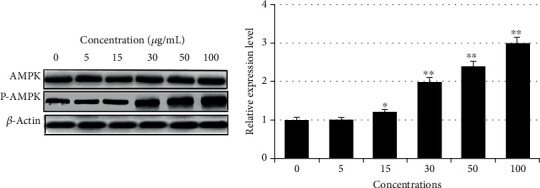
AMPK-to-p-AMPK activation and phosphorylation of RASE1 were determined by western blot after 3T3-L1 preadipocytes were differentiated to adipocytes in the absence and presence of various sample concentrations. Protein levels are expressed as fold change in relation to the control (actin) after normalization. (a) Protein bands visualized after western blotting for AMPK, p-AMPK*α*, and *β*-actin. (b) p-AMPK*α* relative protein expression levels determined by western blotting. Data are mean (*n* = 3) ± SEM (^∗^*p* < 0.01 and ^∗∗^*p* < 0.001 compared to the control).

The effects of extracted stevia compounds of an isolated fraction on the activation of AMPK levels were investigated in 3T3-L1 preadipocytes. After cell treatment with samples and protein extraction, activation (phosphorylation) was determined by western blotting. Results showed that the samples enhanced AMPK-to-p-AMPK activation in a dose-dependent manner (Figure 8).

### 2.7. In Vitro Lipolysis Activity

**Figure 9:**
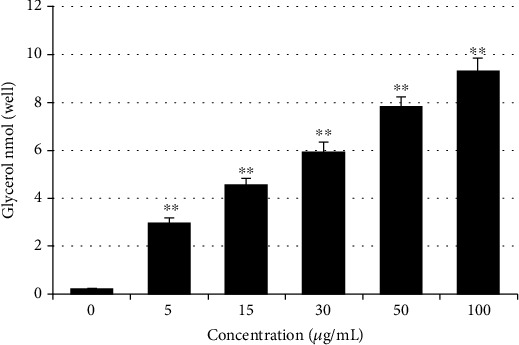
*In vitro* lipolysis potential of extracted stevia compounds and GTS saponin (RASE1). 3T3-L1 premature cells proliferated in DMEM. Differentiated 3T3-L1 cells were treated with samples according to kit instructions and were quantified. Lipolysis potential was calculated in relation to glycerol production (^∗^*p* < 0.01 and ^∗∗^*p* < 0.001 compared to the control).

Lipid breakdown potentials of extracted stevia compounds and green tea seed saponins were investigated *in vitro* using 3T3-L1 preadipocytes. Following cell differentiation and lipid droplet (TGs) accumulation, cells were treated with various safe sample doses. The extracted stevia compound fraction alone and in combination with theasaponin 3 significantly enhanced the lipolysis process in a dose-dependent manner (Figure 9).

### 2.8. In Vivo Antiadipogenic and Lipogenic Effects

#### 2.8.1. Food Intake and Body Weight of Mice

**Figure 10:**
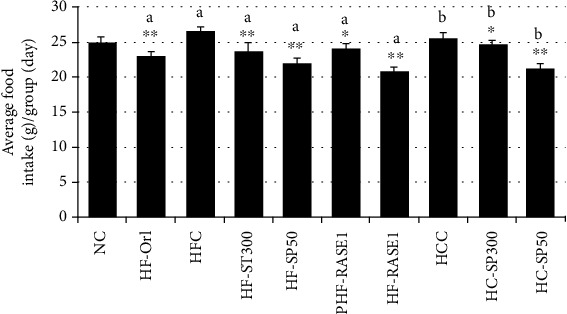
RASE1 effect on the food intake of animals in various groups. Data are the mean (*n* = 6) ± SEM. Data were considered statistically significant at ^∗^*p* < 0.01 and ^∗∗^*p* < 0.001 vs. normal control group. a indicates statistical significance ^∗^*p* < 0.01 for HFC, and b indicates statistical significance ^∗^*p* < 0.01 for HCC.

To investigate obesity modulation by stevia-derived compounds and theasaponin E1 in a mouse model, eight-week-old female ICR mice were randomly assigned to 10 groups, each containing six mice. The first group was fed the ND, six groups were fed the HFD, and the other three groups were fed the HCD. Samples were orally administered daily to the treatment groups. Food intake was measured daily and was slightly different among all groups. Animals treated with 300 mg doses in HFD and HCD groups showed a slight gradual decrease in food intake, and animals of the HFD group treated with stevia and saponins showed the highest gradual decrease in daily food intake compared to controls (Figure 10).

**Figure 11:**
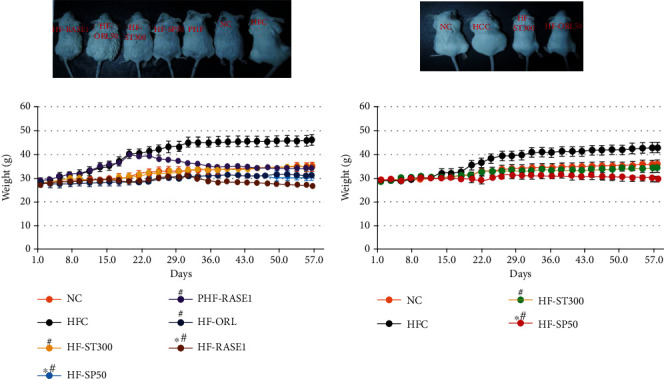
RASE1 effects on mouse body weights of different groups fed carbohydrate- and fat-rich food. Animals were divided into 10 different groups with six animals per group, which were weighed every 24 h for 50 days. Data represent the means (*n* = 6) ± standard deviation. Significance at ^∗^*p* < 0.01 vs. NC and ^#^*p* < 0.01 vs. HFC and HCC, respectively.

After 8 weeks, the total body weights of the mice in the HFD control (HFC) and HCD control (HCC) groups were significantly higher than those in the NC group. Mouse body weights in the HF-100 and HF-300 groups significantly decreased compared with those of the HFC group. The PHF-300 group was fed a HFD for 3 weeks to induce obesity before treatment. After treatment with a 300 mg/kg sample in the PHF-300 group, the weight gain was significantly reduced for 5–8 weeks. The highest body weight reduction was found in the HFD group with a combined sample of stevia and in the saponin SP + ST (RASE1) group (Figure 11).

The extracted stevia sample also suppressed the body weight gains in the HCD group. The significant inhibitory effect on body weight gain was observed during 5–8 weeks in RASE1 (mice fed with stevia extract plus saponin), i.e., RASE1, HF-300, and HC-300 groups. The highest body weight gain was observed in the HFC and HCC groups. Slimming effects were observed in all treatment groups in a dose-dependent manner in mice fed both HCD and HFD. The positive control HF-O group showed comparatively increased body weight gain compared to that of the RASE1 group and almost the same to the HF-300 group. However, gastrointestinal side effects, such as oily and loose stools, were observed.

#### 2.8.2. Effect on Liver and Adipose Tissue Mass Gain

**Figure 12:**
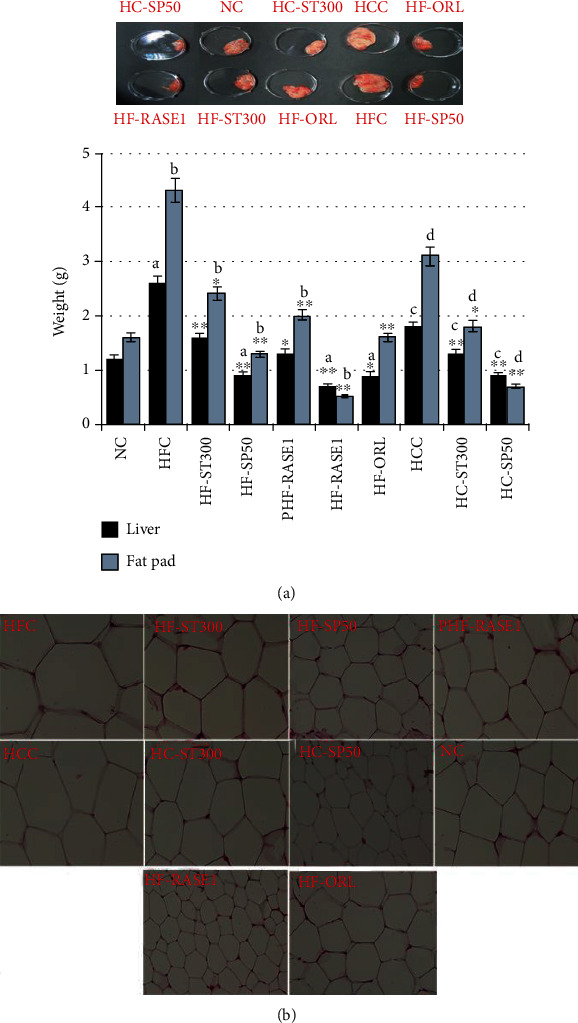
RASE1 effect on liver and adipose tissue size and fat accumulation in various animal groups. (a) Liver and fat tissues (perirenal, epididymal, and mesenteric WAT) were collected from each animal after surgery and were weighed. (b) Adipose tissues were stained, and adipocyte size was determined in the mice of various groups under a microscope. Data represent means (*n* = 6) ± SEM. Data are significant at ^∗^*p* < 0.01, ^∗∗^*p* < 0.001 vs. control. a indicates statistical significance ^∗^*p* < 0.01 for HFC, and b indicates statistical significance ^∗^*p* < 0.01 for HCC.

The inhibitory effects of the extracted stevia fraction and RASE1 on adipose tissue and liver weight gain were determined by collecting and weighing adipose and liver tissues of all animals of each group after dissection. Liver and adipose tissues were measured, and data of the same group of mice are presented as means.  Figure 12 shows that the liver was fatty and had a greater weight gain in HFC and HCC groups. These were significantly decreased in the treatment groups compared to HFC and HCC groups (*p* < 0.05). Liver and WAT weight in the treatment group of mice was lowest in the HF-300 group compared with that of the NC and positive control (HF-ORL) groups. Among all groups, the lowest WAT and liver weight were calculated for the ST + SP group. Adipocyte size of the epididymal adipose tissue in various groups after dissection and staining is presented in Figure 12(b). Adipocyte size was reduced in treatment groups compared to the NC. Adipocytes were abnormally expanded and of greatest size in HFD and HCC animal groups.

### 2.9. Serum and Fecal Biochemical Parameters

**Figure 13:**
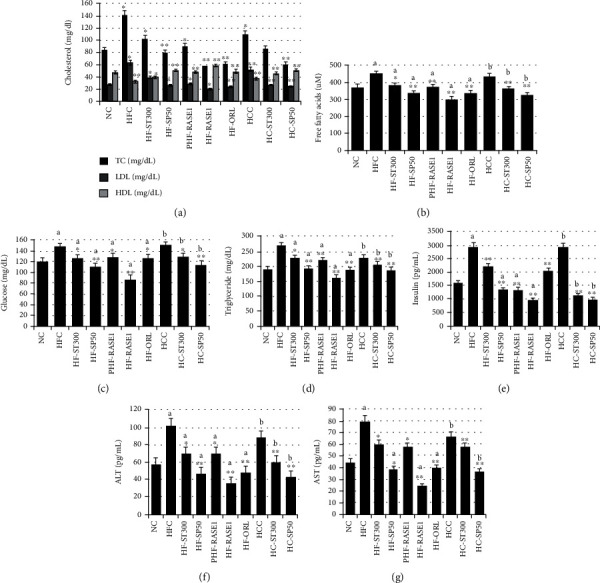
*Stevia* compounds and green tea seed saponin (RASE1) effects on serum TG, TC, HDL, LDL, glucose, ALT, and AST levels in various animal groups. Animals were treated with or without different RASE1 doses, and blood samples were collected from each animal at the end of the experiment and analyzed for biochemical parameters of obesity using the corresponding ELISA kits. Data are the mean values ± SEM. ^∗∗^*p* < 0.01 and ^∗∗^*p* < 0.001 vs. control (NC). a indicates statistical significance ^∗^*p* < 0.01 for HFC, and b indicates statistical significance ^∗^*p* < 0.01 for HCC.

Blood was collected in heparin tubes from all animals of each group for elucidating the effects of the extracted stevia compounds and theasaponin E1. Results of serum biochemical parameters are shown in Figure 13. Serum levels of TG, TC, FFA, LDL cholesterol, glucose, ALT, and AST were higher in HFC and HCC groups than in NC and treatment groups. A notable increase in HDL cholesterol was observed in all treatment groups compared with HFC, HCC, and NDC groups; however, the highest increase of HDL was in the HF-RASE1 group. Extracted stevia compound doses alone and in combination with green tea saponin E1 administration caused a significant (*p* < 0.05) decrease in TG, TC, FFA, LDL, cholesterol, glucose, ALT, and AST levels. Additionally, 300 mg/kg of RASE1 effectively decreased the above parameter levels more than the NC. The abovementioned obesity biomarkers were highly suppressed in HF-RASE1 compared to those of the positive control group (HF-ORL) and were highly reduced in HC-RASE1.

**Figure 14:**
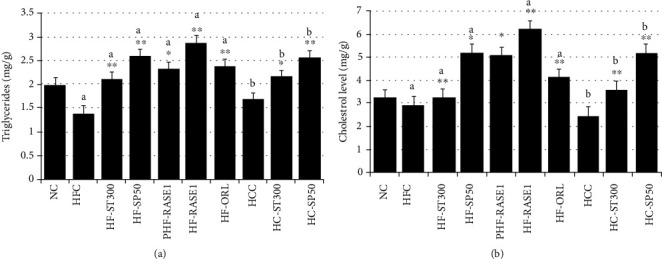
The RASE1 effect on fecal TG and TC levels of various animal groups. Fecal samples collected during the last week of the experiment were processed. TG and TC concentrations were measured in the samples of each animal group using the corresponding kits. Data represent means (*n* = 6) ± SEM. Data are significant at ^∗^*p* < 0.01, ^∗∗^*p* < 0.001 vs. control (NC). a indicates statistical significance ^∗^*p* < 0.01 for HFC, and b indicates statistical significance ^∗^*p* < 0.01 for HCC.

TG and TC fecal levels were collected and analyzed after 5 weeks. Significantly higher TG and TC levels were observed in the treatment groups compared with those in NDC, HFC, and HCC groups. The highest TG and TC fecal levels were recorded for HF-RASE1 followed by HC-RASE1 (Figure 14).

### 2.10. Effects of Stevia and Green Tea Constituents on Serum Insulin, Leptin, Adiponectin, IL-10, IL-6, and TNF-*α* Levels

**Figure 15:**
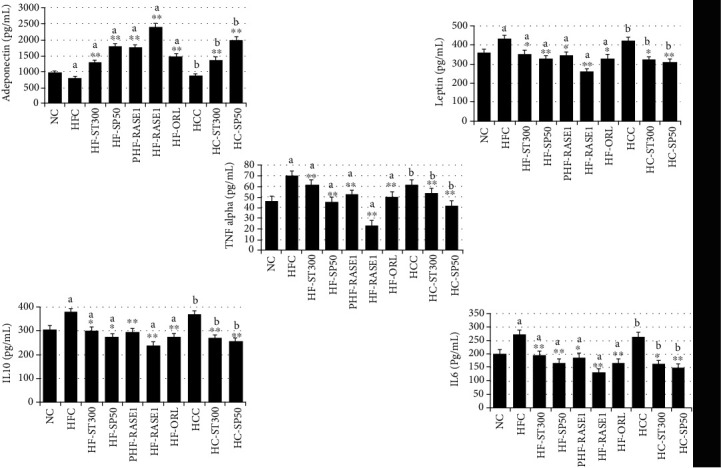
RASE1 effect on serum adipokine levels in different mouse groups. Animals were treated with or without different RASE1 doses and blood samples collected from each animal after the end of the experiment were analyzed for biochemical parameters of obesity using the corresponding ELISA kits. Data are the mean values ± SEM. ^∗∗^*p* < 0.01 and ^∗∗^*p* < 0.001 vs. control (NC). a indicates statistical significance ^∗^*p* < 0.01 for HFC, and b indicates statistical significance ^∗^*p* < 0.01 for HCC.

Serum insulin, leptin, IL-10, IL-6, and TNF-*α* levels in HFC and HCC groups significantly increased compared with those in the NC and all treatment groups, where levels were suppressed. The lowest levels were noted in the HC-RASE1 group, which received extracted compounds from stevia plus green tea seed extracted saponin doses. However, serum adiponectin levels were significantly increased by stevia and saponin administration in HC-RASE1, HF-RASE1, HF-SP50, and HF-ST300 groups. Sample effects on serum insulin, leptin, adiponectin, IL-10, IL-6, and TNF-*α* levels are shown in Figure 15.

### 2.11. Effects of Isolated Pure Compound Stevia Fraction and Green Tea Seed Saponin on Adipose Tissue Leptin, IL-6, IL-10, and Adiponectin Levels

**Figure 16:**
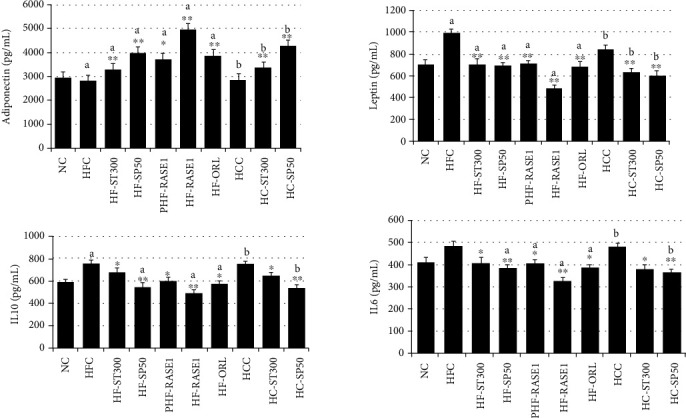
The effect of the standardized formulation of stevia compounds and green tea seed saponin (RASE1) on adipose tissue adipokine levels in different mouse groups. Data are expressed as mean ± SEM (*n* = 6). ^∗∗^*p* < 0.01 and ^∗∗^*p* < 0.001 vs. control. a indicates statistical significance ^∗^*p* < 0.01 for HFC, and b indicates statistical significance ^∗^*p* < 0.01 for HCC.

Leptin, IL-6, IL-10, and adiponectin concentrations in mesenteric adipose tissue were determined after animal dissection and adipose tissue collection. As shown in Figure 16, elevated leptin, IL-6, and IL-10 concentrations were higher in HFC and HCC adipose tissues. In contrast, mice of the treatment group exhibited significant decreases in leptin, IL-6, and IL-10 adipose tissue concentrations than NC group mice. Treatment with stevia extract plus saponins significantly decreased leptin, IL-6, and IL-10 adipose tissue concentrations in HF-RASE1 mice. Adiponectin concentrations in mesenteric WAT were more significantly increased in treatment groups dose-dependently than in HCC, HFC, and NC group mice.

### 2.12. GTE on mRNA Expression of Energy and Lipid Metabolism Enzymes and Transcription Factors

**Figure 17:**
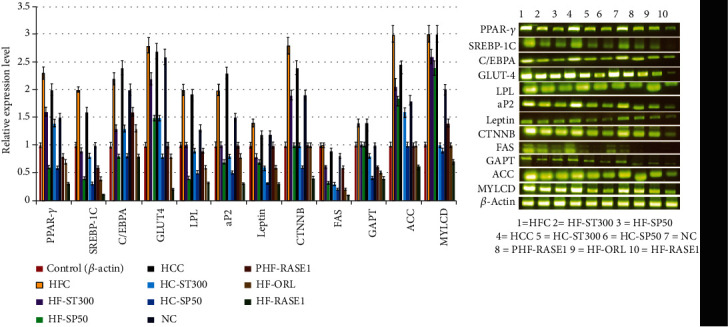
Adipogenesis, lipogenesis, and lipid metabolism mRNA expression levels determined by RT-PCR analysis in mesenteric adipose tissue from mice of different groups. (a) Relative expression levels of various genes normalized to control (actin). (^∗^*p* < 0.01 and ^∗∗^*p* < 0.001 compared to the control). (b) Gene expression levels of adipogenesis, lipogenesis, and lipid metabolism genes of treated and control groups presented in the form of a gel band of the PCR product.

The effect of the isolated stevia fraction of stevioside and rebaudioside and its combination with green tea seed saponin E1 on the transcriptional factors and genes involved in adipogenesis, lipogenesis, and lipid metabolism was determined in the adipose and liver tissue of mice analyzed by RT-PCR. Mesenteric adipose and liver tissues were used to evaluate the effect of the samples on lipid and energy metabolism pathway biomarkers. In liver tissue, ACC-1, GPAT, FAS, and MLYCD mRNA expression levels decreased in the treatment group in a dose-dependent manner compared with those of the HFC and HCC groups (Figure 17). Stevia extract and saponins reduced the expression of lipogenesis- and adipogenesis pathway-related genes in mesenteric adipose tissue. mRNA expression levels of transcription factors (PPAR*γ*, C/EBP*α*, and SREBP) were more downregulated in the treatment groups than in the HFC and HCC groups. Levels of target gene lipoprotein lipase (LPL), adipocyte protein 2 (aP2), and leptin of lipogenesis and adipogenesis transcription factors in mesenteric WAT also decreased with an increased dose of sample and was most effective and significant in the RASE1 group mice, which received combined extracted stevia compound and green tea seed saponin.

## 3. Discussion

Obesity is a common disorder caused by interactions of environmental, genetic, and nutritional factors, and its pervasiveness is accelerating worldwide. Socioeconomic changes, extensive consumption of calorific foods, and increasingly sedentary lifestyles are the predominant causative factors for abnormal adipose tissue development and rise in obesity. Abnormalities, both in adipose tissue development and preadipocyte differentiation to mature adipocytes, are directly linked to obesity.

Obesity has become a critical concern worldwide due to its association with comorbidities, including cancer, cardiovascular diseases, and diabetes [38]. Adipose tissue is an endocrine organ, thus, plays a critical role in the survival of an individual; however, its dysfunction or excess accumulation is directly linked to obesity [39, 40]. The journey from multipotent mesenchymal stem cells (MSCs) to the formation of mature adipocytes is a well-orchestrated program requiring the expression of several genes, their transcriptional factors, and signaling intermediates from numerous pathways. Understanding all intricacies of adipogenesis is vital for countering the current obesity epidemic as the limited understanding of these intricacies is the main barrier to developing potent therapeutic strategies against obesity [41].

Adipogenesis is determined by gene expression and protein functions dictating adipocyte phenotypes [42]. Hyperplasia and hypertrophy of WAT through adipogenesis lead to obesity [38, 42, 43]. The adipogenesis cellular process involves the commitment of MSCs to the adipocyte lineage, followed by mitotic clonal expansion with DNA replication and cell duplication, and, finally, terminal differentiation, involving the expression of genes and transcriptional factors, including the C/EBP family and PPAR*γ*, and a dramatic increase in lipogenesis and induction of lipogenic genes, including ACC, FAS, and aP2 [44, 45]. Preadipocyte differentiation into mature adipocytes is also influenced by other factors, such as insulin, which is one of the potent adipogenic hormones that induce the transcription of various positive regulators of adipogenesis [46, 47].

AMPK inhibits de novo synthesis by inactivating ACC-1 and FAS, which catalyzes key regulatory steps in fatty acid and sterol synthesis and activates FAO [48]. AMPK also regulates ligand-activated transcriptional factors, including PPAR*γ*, C/EBP*α*, and SREBP, which are central regulators of adipogenesis and lipogenesis [49, 50]. Thus, the AMPK pathway is a potential therapeutic target for metabolic disorders.

Lipolysis entails TG hydrolysis into glycerol and FFAs within the cell. Glycerol and FFAs are then released into the bloodstream or culture media. Lipolysis occurs in essentially all cells but is most abundant in WAT and BAT. Deficiencies in lipolysis lead to increased intracellular lipid accumulation, resulting in abnormal cellular physiology, hyperlipidemia, and insulin resistance. Lipolysis can be induced by catecholamines and certain hormones.

Stevia extract is used as a natural sweetener in various parts of the world; however, its biological effects, especially antiobesity activity, have not been well documented. Green tea (*C. sinensis*) contains many bioactive compounds, including catechins, caffeine, saponins, theanine, vitamins, mineral oil, and trace elements [51, 52]. Green tea components have many biological and biochemical effects, including antimutation [53], anticarcinogenesis [54], antioxidation [55], apoptosis-inducing [41], and antiangiogenesis effects. Major bioactive compounds present in green tea seeds include flavonoids, EGCG, saponin glycosides, kaempferol, and naringenin [56–59].

The present study evaluated the effect of standardized green tea seed extract, saponin E1, and extracted stevia compound supplementation on adipocyte differentiation, angiogenesis, and body weight gain in HFD and HCD mice using biochemical markers to elucidate the molecular mechanism underlying such an effect.

Diets are prevalent in inducing obesity, whereas natural products show antiobesity effects involving diverse mechanisms, including appetite, food intake, FAO, lipogenesis, and adipogenesis regulation. As with tumors, the growth and expansion of adipose tissue require new blood vessel (angiogenesis) formation to provide oxygen and nutrients to adipocytes, which expand both in size and number. Thus, angiogenesis inhibition also inhibits adipogenesis by the reduction of the adipocyte numbers and fat content [49]. Results from Oil red O staining demonstrated that RASE1 prevents preadipocyte differentiation and lipid accumulation. In the *in vivo* experiment, aqueous extract of green tea seed effectively protected HFD mice, whereas a HCD-induced body weight gain. Adipose tissue and liver masses correlated with body weight in mice fed RASE1, suggesting that the RASE1-mediated decrease in body weight could be attributed to a reduction in adipose tissue and liver mass, independent of food intake. In this study, green tea seed extract significantly lowered serum lipid and glucose levels compared with those of the control group. Treatment also lowered serum and mesenteric adipose tissue levels of leptin and IL-6, whereas adiponectin levels were significantly increased. NF-*κ*B pathway and proinflamatory cytokines such as IL-10, IL-6, and TNF-*α* promote inflammation and exacerbate obesity.

RASE1 suppressed transcription factors and their target genes (LPL, aP2, and leptin) expression in the mesenteric adipose tissue of treatment group mice. *In vivo* studies in mice revealed that RASE1 does not affect food intake, rather, it suppresses anabolic pathways and stimulates catabolic pathways [60]. Traditional medicine uses natural products to enhance efficacy and, at the same time, reduces side effects. The RASE1 antiobesity effect was largely influenced in HCD mice treated with 300 mg/kg of RASE1.

In this study, the extracted stevia compounds (stevioside and rebaudioside A) and green tea seed saponin theasaponin E1 exhibited higher antiangiogenesis, antiadipogenesis, antilipogenic, and lipolysis effects synergistically compared to those exhibited by individual compound. RASE1 suppressed NF-*κ*B, pathway and proinflammatory cytokines IL-10, IL-6, and TNF-*α* level, which are involved in inflammation and progression of obesity.

The RASE1 effect on endothelial cell tube formation was examined by quantitative analysis of tube length formed on Matrigel (a reconstituted extracellular matrix preparation of EHS mouse sarcoma). RASE1 exhibited a significant inhibitory effect on capillary tube formation in a dose-dependent manner when HUVECs were supplemented with increasing doses of RASE1. The lowest GTE concentration that significantly inhibited tubular structure formation was 5 *μ*g/mL, with complete disruption of capillary tubes observed at 100 *μ*g/mL. Results demonstrated that RASE1 effectively inhibits HUVEC tube formation on Matrigel.

The RASE1 effect on VEGFR-2 expression and the PI3K/AKT/ERK pathway in HUVECs was evaluated using concentration-dependent experiments. RASE1 reduced VEGFR-2 and VE-cadherin complex expression via NF-*κ*B downregulation, which are important growth factors for proliferation and vascular remodeling. Various growth factors have been documented to play an important role in PI3K/AKT and ERK pathway activation [61]. RT-PCR results showed AKT and ERK mRNA level inhibition via VEGFR-2 downregulation in a dose-dependent manner. Treatment with a median concentration (100 *μ*g/mL) of RASE1 led to significant PI3K, AKT, ERK, VEGFR-2, NF-*κ*B, and *β*-catenin mRNA expression inhibition. Thus, the results showed that RASE1 regulates PI3K/AKT and ERK pathways through VEGF expression.

These results provide a molecular basis for understanding green tea seed extract saponins and stevia extract compounds in combination (RASE1) and antihyperlipidemic and fat-pad lowering effects. RASE1 treatment played an important role in lipid and pathological inflammation regulation in HFD and HCD mice. These results demonstrated that RASE1 displays remarkable bioactivity for the prevention of angiogenesis and obesity-related metabolic disorders by inhibiting adipocyte differentiation, angiogenesis, and body weight gain, and by reducing body fat pad weight and serum lipid levels *in vivo*.

## 4. Methods

The experimental research and studies on plants, including the collection of plant materials, comply with relevant institutional, national, and international guidelines and legislation.

### 4.1. Extraction of Theasaponin E1 from Green Tea Seed and Sativoside and Rebaudioside A from Stevia rebaudiana

Green tea (*Camellia sinensis*) seeds were collected from the Myungin Shin Gwang Su tea garden (Suncheon, Korea). Seeds were dried, dehulled, and ground into powder. The powder (3 kg) was defatted with n-hexane (4 L) under sonication at 30°C for 5 h and then dried. Defatted seed powder was further extracted by refluxing with 70% ethanol at 60°C for 8 h. The resulting extract was filtered, concentrated using a rotatory vacuum evaporator (SB-100, Eyela), freeze-dried, and weighed. The extract was resubjected to extraction with butanol and water mixture and concentrated with the rotatory evaporator. Saponin extraction from crude extracts was carried out using nonpolar macroporous resins (D101). Resins were thoroughly washed twice with ethanol followed by distilled water. Approximately 10 g of the extract was dissolved in 30 mL of double-distilled water, mixed with washed resin, and kept overnight at room temperature. The extract (50 g) was dissolved in 100 mL of double-distilled water and passed through the resin column first eluted with 0.4 N NaOH, followed by extract and resin mixture neutralization with HCl, and reelution with 100% ethanol. This saponin mixture was subjected to column chromatography using the C18 column. The mixture was first eluted with 10% MeOH, followed by elution with 60% MeOH, and finally with 100% MeOH to obtain the saponin mixture. Pure saponin (theasaponin E1) was then isolated from this fraction by preparative HPLC (Shimadzu Co., Kyoto, Japan) equipped with a photodiode array detector. The extract was separated on the Luna C-18(2) reverse phase column (250 × 21.2 mm, 15 *μ*m; Phenomenex, Inc., Torrance, CA, USA) at 35°C. Solvent A was methanol, and solvent B was distilled water containing 0.1% formic acid. The nonlinear gradient system used was A/B (74 : 26) to A/B (74.8 : 25.2) for 33.5 min to A/B (100 : 0) for 2 min, held at A/B (100 : 0) for 10 min, and then A/B (74 : 26) for 12 min. Components were detected at 210 nm. The flow rate was 7 mL/min. Saponin fractions obtained were analyzed by LC-MS and NMR to identify and characterize the saponins present. Major saponins detected in this fraction were theasaponin E1 (C59H90O27), theasaponin C, assamsaponin A (C57H88O25), theasaponin E3 (C57H88O26), theasaponin A1 (C57H90O26), assamsaponin B (C61H92O28), and theasaponin A3 (C61H94O28). Fr2 and Fr3 were obtained from the saponin mixture by preparative HPLC (Develosil ODS-HG-5, MeCN-0.05% aqueous TFA 55 : 45, 4 mL/min); theasaponin E1 was isolated from the saponin fraction by further subjection and purification with preparative HPLC.

### 4.2. Extraction of the Sativoside and Rebaudioside Fractions from Stevia

Dried stevia leaves were ground into a fine powder and extracted with 70% EtOH at 60°C for 6 h by continuous reflux in a heating mantle. The mixture was filtered using filter bags followed by a super jet filter and concentrated with a rotatory vacuum evaporator (SB-100, Eyela). The concentrated extract was collected as crude SR extract and dried by lyophilization. Crude extract was further extracted with nonpolar macroporous resin. The extract and resin mixtures were loaded into a column of 500 mL and eluted with 80% EtOH, resulting in a dark reddish-brown fraction. Resins were washed twice with 2DH_2_O, mixed with the fraction, incubated overnight in the column, and then eluted with 100% ethanol, resulting in the required fraction. In this process, unwanted color compounds were separated and discarded, and the brownish fraction was obtained. This fraction was dried and further fractionation and purification was conducted by column chromatography using the C-18 column. First, the column was washed with 10% EtOH. Approximately 50 g of the fraction was dissolved in 100 mL of 100% EtOH and loaded into the C-18 Luna column. The fraction was first eluted with 60% EtOH, the second fraction was eluted with 100% EtOH, and another fraction, believed to contain stevioside and rebaudiosides, was obtained. This fraction was reloaded into the column and eluted with 100% EtOH. The resulting fraction was concentrated and analyzed. Identification and determination of the isolated compounds were conducted by LC/TOF-MS and NMR. Rebaudioside A and stevioside with small fractions of rebaudiosides B and D were detected in the isolated purified fraction. The fraction was further purified from the traces of other compounds by eluting again with 100% EtOH using the C18-Luna column. The purified final fraction contained stevioside and rebaudioside A (76.5% and 32.1%, respectively).

### 4.3. Sample Preparation

Green tea seed isolated theasaponin E1 (C_59_H_90_O_27_), and isolated stevia fractions comprising stevioside (C_38_H_60_O_18_) and rebaudioside A (C_44_H_70_O_23_) were used to analyze their antiobesity effects. Pure isolated compounds, i.e., the fraction of stevioside and rebaudioside A purified from stevia leaf extracts and theasaponin E1, were used for investigating the synergistic antiobesity effects both *in vitro* and *in vivo*. The combination of sativoside, rebaudioside A, and theasaponin E1 was named the standardized formulation of RASE1. The ratio of the sativoside and rebaudioside A fraction to theasaponin E1 in RASE1 was 5 : 1, respectively, based on the results of the toxicity and cell viability experiments. For the *in vitro* studies, 100 *μ*g/mL of RASE1 was used as the highest concentration, while for *in vivo* experiments, 300 mg/kg body weight of RASE1 was used as the highest dose based on the acute toxicity experiment on mice. All final products were obtained in the dried form as powder and were dissolved in distilled water for all experiments.

For *in vitro* and in vivo experiments, we followed our previously described methods (Chaudhary et al. 2015 [62] and Khan et al. 2018 [63] with some modifications).

### 4.4. Cell Culture

Human umbilical vein endothelial cells (HUVECs) and the 3T3-L1 cell line were obtained from the Korean Cell Line Bank, Seoul, Korea. HUVECs were cultured in EBM-2 (Clonetics, Walkersville, MD, USA) supplemented with EGM-2 using the Single Quots Kit (Clonetics) at 37°C in a humidified 5% CO_2_ incubator. Then, 3T3-L1 cells were cultured in DMEM medium (Gibco, Grand Island, NY, USA) supplemented with NaHCO_3_ (3.7 g/L), 100,000 IU/L penicillin, 100 mg/L streptomycin, and 10% (*v*/*v*) fetal bovine serum (FBS).

### 4.5. Cell Viability Assays

Sample toxicity was checked to determine safe sample concentrations. Cell viability was determined by 3-(4,5-dimethylthiazol-2-yl)-2,5-diphenyltetrazolium bromide (MTT) assay using 96-well plates. HUVECs and 3T3-L1 cells (1 × 10^4^ cells/well) were seeded into a 96-well plate separately for 24 h and treated with different concentrations of stevia fraction, theasaponin E1, and their combination (RASE1) for 24 h at 37°C in a humidified 5% CO_2_ incubator. Then, 0.5% MTT solution (Sigma) was added to the medium and incubated for 4 h at 37°C. MTT solution was aspirated, and dimethyl sulfoxide (Sigma) was added to dissolve the formazan crystals for 15 min. Absorbance was measured at 540 nm using a microplate reader (Biochrom Ltd., Cambridge, UK). Cell viability was calculated as the relative percentage compared to the control (cells untreated with sample).

Cell viability experiments were performed 2 times in triplicate.

### 4.6. Antiangiogenesis Assays

An *in vitro* angiogenesis assay was performed using HUVECs. Cells were cultured in 96-well microtiter plates coated with 100 *μ*L/well of Matrigel (BD Bioscience, MA, USA) and were allowed to solidify at 37°C. HUVEC suspensions in medium were added to Matrigel-coated wells (1 × 10^4^ cells per well) and were incubated for 4 h in 5% CO_2_ at 37°C. Different concentrations of stevia extract, saponins, and combined products were added to the wells and further incubated for 4 h. Tube formation was observed and photographed using a phase contrast inverted microscope (Nikon, Tokyo, Japan). The tube length of five photographs obtained from random cell culture fields in each well was analyzed and analyzed using ImageJ version 1.53 k (NIH, Bethesda, MD, USA) software with an angiogenesis analyzer. The total tube length was calculated and compared with that of the controls. Experiments were performed 2 times in triplicate.

### 4.7. mRNA Gene Expression Levels of Angiogenesis

The effect of samples on angiogenesis suppression was determined by the effect of samples on mRNA expression levels of genes of angiogenesis promoting signaling molecules by RT-PCR. Relative gene expression levels were calculated for each transcriptional factor and signaling proteins involved in the pathway.

For angiogenic biomarker analysis, HUVECs were seeded into a six-well plate at a density of 9.6 × 10^4^ cells per well. Cells were maintained in EBM-2 complete medium to up to 70% confluency. Then, different concentrations of stevia extract and saponins were added to the wells containing cells and were incubated for 24 h. Total RNA was extracted from a cell monolayer using an RNA extraction kit (Qiagen, USA) according to the manufacturer's instructions. cDNA synthesis was performed with 1 *μ*g of total RNA using the RevertAid Premium First Strand cDNA Synthesis Kit (Thermo scientific, EU). RT-PCR was used to analyze VEGFR-2, B-catenin, PI3K, VE-cadherin, AKT, NF-*κ*B, and ERK2 mRNA expression levels using gene-specific primers. PCR reactions were performed using a PCR kit (Thermofisher). PCR reactions consisted of an initial denaturing cycle at 94°C for 3 min, followed by 30 amplification cycles of 94°C for 30 s, 60°C for 45 s, and 7°C for 1 min. One additional cycle of 72°C for 7 min was run to allow trimming of incomplete polymerization. Amplified products were separated by electrophoresis on a 1.5% agarose gel and were visualized by UV transillumination. Gene expression analysis experiments were performed 2 times in triplicate.

### 4.8. Antiadipogenesis, Antilipogenesis, and Lipolysis Assays

Antiadipogenesis and antilipogenesis effects of the samples were analyzed by the synthesis and accumulation of fat in 3T3-L1 cells using an Oil red O staining assay, and sample effects on gene expression levels were analyzed by RT-PCR. Similarly, sample lipolysis potential was determined *in vitro* by a commercial specialized kit using 3T3-L1 cells.

### 4.9. Oil Red O Staining Assay

3T3-L1 cells were cultured (1 × 10^4^) in DMEM using 96-well microtiter culture plates. Two days after reaching confluency, cells were kept for another 24 h in this state to arrest cell division. At this point (day 0), the culture medium was changed to adipogenesis induction medium or differentiation induction medium (1 mM dexamethasone, 0.5 mM 3-isobutyl-1-methylxanthine, and 10 *μ*g/mL insulin). After 2 days, cells were maintained in maintenance medium (10 *μ*g/mL insulin in culture medium). Maintenance medium was changed every 2 days until day 8. Cells were treated with different safe concentrations of samples of the extracted stevia purified compound fraction, green tea seed saponins, and a combination of these at each addition of differentiation induction medium and maintenance medium. Cells were washed with PBS and fixed with 10% formalin for 1 h. Again, the cells were washed with 60% 2-propanol to dryness and then incubated with Oil red O working solution for 3 h. Once again, the cells were washed four times with distilled water, and completely dried cells were washed with 100% 2-propanol to extract the staining dye in the cells. The absorbance of the extracted Oil red O solution was measured at 520 nm using a 96-well plate reader. Fast determination experiments were performed 2 times in triplicate.

### 4.10. Adipogenesis and Lipogenesis Gene Expression Levels

The mRNA expression of adipogenesis- and lipogenesis-related genes was analyzed by RT-PCR. The inhibitory effect of the samples on mRNA expression levels of adipogenesis- and lipogenesis-related genes and signal molecules was investigated using gene-specific primers. 3T3-L1 cells were cultured in DMEM supplemented with 10% FBS in a six-well plate and were incubated at 37°C in a CO_2_ incubator. Cells were treated with different concentrations of individual and combined samples and were incubated in the CO_2_ incubator for 24 h. Total RNA was isolated from 3T3-L1 cells using TRI reagent (Sigma Aldrich), followed by DNase treatment and quantification on the NanoDrop 2000 spectrophotometer. cDNA was synthesized from 500 ng total RNA using the Revert Aid Premium First-Strand cDNA Synthesis Kit (Thermo Fisher Scientific) and was quantified by measuring absorption on a NanoDrop spectrophotometer. cDNA was used for real-time qPCR analysis with gene-specific primers. DNA bands were obtained on agarose gel after electrophoresis. Adipogenesis and lipogenesis gene expression level experiments were performed 2 times in triplicate.

### 4.11. In Vitro Lipolysis Assays

The *in vitro* lipolysis potential of RASE1 was investigated in 3T3-L1 cells using the Lipolysis (3T3-L1) Assay Kit (MBS841692). Glycerol released from cells after lipolysis induction was measured using the colorimetric method. Color intensity was directly proportional to the glycerol amount. For the lipolysis assay, 3T3-L1 cells were cultured in DMEM in a 96-well plate and kept in a 5% CO_2_ humidified incubator. After 24 h, when cell confluency reached 70%, the medium was replaced with differentiation induction medium. Cells were kept in this state for 24 h to arrest cell division. After 2 days, the culture medium was replaced with maintenance medium. Cells were then fed with maintenance medium every 2 days for up to 8 days. Samples were added during induction and maintenance media addition. Cells were then treated using a lipolysis assay kit for induction, and lipolysis measurements were obtained. Lipolysis assay experiments were performed 2 times in triplicate.

### 4.12. Western Blotting for Measuring the p-AMPK Level

3T3-L1 cells (1 × 10^4^ cells/well) were cultured in DMEM using 96-well microtiter culture plates. After treatment with various extracted stevia compounds and green tea saponin concentrations, 3T3-L1 adipocytes after differentiation were harvested at subconfluency, washed with PBS buffer, and lysed with radioimmunoprecipitation assay buffer (Sigma-Aldrich, MO, USA) containing phenylmethylsulphonyl fluoride and protease inhibitor cocktail. Cell lysates were sonicated and centrifuged, and supernatants were collected. The supernatant protein concentration was measured using the BCA Protein Assay Reagent (Pierce, Rockford, IL, USA). Approximately 30 *μ*g of protein for each sample was separated by 10% SDS-PAGE and transferred onto nitrocellulose membranes (Bio-Rad, Hercules, CA). Membranes were blocked in Tris-buffered saline-Tween 20 solution containing 3% bovine serum albumin. Membranes were then incubated overnight with AMPK, phospho-AMPK*α* (Cell Signaling Technology, Danvers, MA), and *β*-actin (Santa Cruz, CA) primary antibodies at 4°C. Secondary antibody was conjugated to horseradish peroxidase, and enhanced chemiluminescence (Amersham Bioscience, UK) was undertaken to visualize the protein bands. Bands were analyzed using ImageJ (1.53 k NIH, Bethesda, MD, USA). Experiments were performed 2 times in triplicate.

### 4.13. Animal Experiments

The study was carried out in compliance with the ARRIVE guidelines. All animals used in this study were treated according to the National Institutes of Health Guide for the Care and Use of Laboratory Animals. The experimental protocol was approved by the Chonnam National University Ethical Committee for Animal Studies under the approval number CNU IACUC–YS–2020-1. Sixty 8-week-old female ICR mice (Taconic, Chungbuk, Korea) were randomly assigned to 10 groups containing six mice each after 1 week of acclimation. All animals were housed under standard conditions (12 h light/dark cycle at 22°C and relative humidity 50% ±5%) and were provided diet and water *ad libitum*. The acute toxicity level of the test samples and standards was determined before starting the antiobesity experiment. Control groups were fed a normal diet (normal diet control (NC); NIH #31 M Rodent Diet, Taconic), high-fat diet (high-fat diet control (HFC); 45% fat, D12451 Research Diets, New Brunswick, NJ, USA), and high-carbohydrate diet (high-carbohydrate diet control (HCC); 70% carbohydrate, TD.98090 Harlan Teklad, Seoul, Korea). One group served as a positive control group (HF-ORL) fed a high-fat diet plus 50 mg/kg/d orlistat. Mice in the other groups received a high-fat or high-carbohydrate diet plus doses of either extracted stevia compounds or green tea seed extracted theasaponin E1 alone or combined (RASE1). These were 300 mg/kg/d stevia extracted standardized fraction (HF-ST300), high-fat diet plus 50 mg/kg/d theasaponin E1 (HF-SP50), high-fat diet plus 300 mg/kg/d (stevia extracted standardized fraction plus theasaponin E1 250 + 50; HF-RASE1), high-carbohydrate diet plus 300 mg/kg/d purified stevia compounds (HC-ST300), and high-carbohydrate diet plus 50 mg/kg/d green tea saponin (HC-SP50). Another group was fed a high-fat diet for 4 weeks to induce obesity pretreatment and then fed 300 mg/kg/d RASE1 (PHF-RASE1). All test and control samples were administered by oral gavage once a day, and control groups were administered distilled water without drugs at the same time. Body weight and food intake were measured after every 24 h in each group. At the end of the 8-week experiment, mice were fasted for 12 h, anesthetized with isoflurane, and euthanized through cardiac puncture. Perirenal white adipose tissue (WAT), epididymal WAT, mesenteric WAT, and liver tissue were excised and weighed. Mesenteric adipose and liver tissues were removed under aseptic conditions, snap frozen in liquid nitrogen, and stored at −80°C for RNA and protein isolation. Total RNA was extracted using an RNA extraction kit according to the manufacturer's instructions, and protein concentration was measured by the BCA Protein Assay Reagent (Pierce, Rockford, IL, USA). Serum samples were stored at −80°C until analysis.

### 4.14. Biochemical Analyses of Blood, Serum, and Adipose Tissue

Total cholesterol (TC), triacylglycerol (TG), HDL cholesterol, LDL cholesterol, free fatty acid (FFA), glucose, alanine transaminase (ALT), and aspartate transaminase (AST) levels in serum were measured according to kit manufacturer's instruction. The serum insulin level was measured by immunoassay using a mouse insulin ELISA kit. Leptin, adiponectin, IL-6, IL-10, and TNF-*α* serum levels and mesenteric adipose tissue concentrations of leptin, adiponectin, and IL-6 were measured using a mouse ELISA kit. The kits used in these experiments were the Adiponectin Mouse ELISA kit (ab108785), Mouse Aspartate Aminotransferase ELISA Kit (MBS450720), Leptin Mouse ELISA kit (ab100718), IL-6 Mouse ELISA kit (ab100712), Free Fatty Acid Assay Kit-Quantification (ab65341), IL-10 Mouse ELISA kit (ab100697), TNF-*α* Mouse ELISA kit (ab108910), Cholesterol Assay Kit-HDL and LDL/VLDL (ab65390), Mouse Triglyceride ELISA Kit (MBS726589), Mouse Alanine Aminotransferase ELISA Kit (MBS264717), Mouse Insulin ELISA Kit (MBS038565), and Mouse Glucose ELISA Kit (MBS7200879).

### 4.15. RT-PCR Analysis of Adipogenesis, Lipogenesis, Lipid Metabolism, and Browning Effects

Inhibitory effects of stevia extract on mRNA expression levels of adipogenesis- and lipogenesis-related genes and signal molecules were investigated by RT-PCR analysis using gene-specific primers. Mouse liver tissues were processed for RNA extraction using an RNA extraction kit (Qiagen). Total RNA after isolation and purification was quantified on a NanoDrop 2000 spectrophotometer and was reverse transcribed to cDNA using the Revert Aid Premium First-Strand cDNA Synthesis Kit (Thermo Fisher Scientific). cDNA was quantified by observing absorption on a NanoDrop spectrophotometer and then used for real-time qPCR with gene-specific primers for adipogenesis- and lipogenesis-related genes. DNA bands were obtained on an agarose gel after electrophoresis.

### 4.16. Fecal Lipid Concentration

For fecal lipid analysis, feces were collected for 3 weeks at the beginning and end of the experiment. Fecal samples were cleaned and dried at 70°C for 1 h. Then, 100 mg aliquots of feces were incubated with 2 mL of chloroform methanol (2 : 1) at 60°C for 30 min with constant agitation and then centrifuged. Then, 1 mL of water was added to the supernatant, vortexed, and centrifuged (2,000 rpm for 10 min). The lower phase was collected in a new tube, and the sample was evaporated to dryness and resuspended in 0.5 mL of Triton X-100/methanol (2 : 1). TC and TG quantities in fecal lipid extracts were measured according to the kit manufacturer's instructions.

### 4.17. Statistical Analysis

Data are expressed as **means** ± **SEM**. Differences among groups were tested by one-way ANOVA followed by post hoc comparisons by the Tukey–Kramer multiple comparison test (IBM SPSS Statistics 21.0). Data normality was determined (**p** > 0.05) before performing ANOVA. Values of **p** < 0.05 were considered statistically significant.

## 5. Conclusions

In summary, the pure fractions (stevioside and rebaudioside A) extracted from *S. rebaudiana* and green tea seed extracted saponin E1 showed antiangiogenic, antiadipogenic, and antilipogenic effects in obesity model cells and animals. However, the highest antiobesity effects were found when these natural products were combined in a standardized formulation (RASE1). The animal study also demonstrated the antiobesity effect of these natural products. These results showed that *stevia* and green tea combined natural products (RASE1) decreased the body weight, adipose tissue accumulation, serum TG, TC, and LDL-C levels, and lipid levels of HFD mice, and increased serum HDL level and suppressed the expression of transcriptional factors and genes involved in fat production and inflammation. Results of the present study indicate that *stevia* and green tea are functional foods with medicinal effects for treating and preventing obesity and related diseases.

## Data Availability

The data presented in this study are available on request from the corresponding authors.

## Abbreviations

AMPK: AMP-activated protein kinase
Akt: Protein kinase B
ALT: Alanin transaminase
AST: Aspartate transaminase
aP2: Adipocyte fatty acid-binding protein
BMI: Body mass index
BAT: Brown adipose tissue
FGF-2: Basic fibroblast growth factor
C/EBP*α*: CCAAT enhancer-binding protein alpha
COX-2: Cyclooxygenase-2
ACC: CoA carboxylase
FAS: Fatty acid synthase
GM-CSF: Granulocyte-macrophage colony-stimulating factor
HDL: High-density lipoprotein
HGF: Hepatocyte growth factor
HUVECs: Human umbilical vein endothelial cells
LDH: Lactate dehydrogenase
NF-*κ*B: Nuclear factor-kappa B
ORAC: Oxygen radical absorbance capacity
PI3K: Phosphoinositide 3-kinase
PPAR*γ*: Peroxisome proliferator-activated receptor *γ*
VE-cadherin: Vascular endothelial-cadherin
TGF-beta: Transforming growth factor-beta
VEGF: Vascular endothelial growth factor
VEGFR-2: Vascular endothelial growth factor receptor-2
RASE1: Rebaudioside A, sativoside, and theasaponin E1
SCD1: Stearoyl-coenzyme A desaturase-1
SREBP-1c: Sterol regulatory element-binding protein
TNF-*α*: Tumor necrosis factor *α*
WAT: White adipose tissue.

## Additional Points

*Sample Availability*. Samples of the compounds are available from the authors.

## Supplementary Materials

Supplementary 1 File 1: compound isolation and purification process.

Supplementary 2 File 2: stevioside and rebaudioside A identification and quantification BY LC-QTOF-MS.

Supplementary 3 File 3: stevioside and rebaudioside A structure elucidation by NMR.

Supplementary 4 File 4: theasaponin E1 isolation and quantification BY LC-QTOF-MS.

Supplementary 5 File 5: theasaponin E1 structure elucidation by NMR.

Supplementary 6 File 6: primers sequences used for PCR in this study.

## References

[1] Fox C. S., Massaro J. M., Hoffmann U. (2007). Abdominal visceral and subcutaneous adipose tissue Compartments. *Circulation*.

[2] Fabbrini E., Sullivan S., Klein S. (2010). Obesity, and nonalcoholic fatty liver disease: biochemical, metabolic, and clinical implications. *Hepatology*.

[3] Muir L. A., Neeley C. K., Meyer K. A. (2016). Adipose tissue fibrosis, hypertrophy, and hyperplasia: correlations with diabetes in human obesity. *Obesity*.

[4] Hammond R. A., Levine R. (2010). The economic impact of obesity in the United States. *Diabetes, Metabolic Syndrome and Obesity targets and therapy*.

[5] Ng M., Fleming T., Robinson M. (2013). Global, regional, and national prevalence of overweight and obesity in children and adults during 1980–2013: a systematic analysis for the global burden of disease study. *The lancet*.

[6] Al-Quwaidhi A., Pearce M., Critchley J., Sobngwi E., O’Flaherty M. (2014). Trends and future projections of the prevalence of adult obesity in Saudi Arabia, 1992–2022. *Eastern Mediterranean Health Journal*.

[7] Letra L., Santana I., Seiça R. (2014). Obesity as a risk factor for Alzheimer’s disease: the role of adipocytokines. *Metabolic Brain Disease*.

[8] Burhans M. S., Hagman D. K., Kuzma J. N., Schmidt K. A., Kratz M. (2011). Contribution of adipose tissue inflammation to the development of type 2 diabetes mellitus. *Comprehensive Physiology*.

[9] Park Y.-M., Myers M., Vieira-Potter V. J. (2014). Adipose tissue inflammation and metabolic dysfunction: role of exercise. *Missouri medicine*.

[10] Sakamoto T., Nitta T., Maruno K. (2016). Macrophage infiltration into obese adipose tissues suppresses the induction of UCP1 level in mice. *American Journal of Physiology. Endocrinology and Metabolism*.

[11] Mathis D. (2013). Immunological goings-on in visceral adipose tissue. *Cell Metabolism*.

[12] Reue K., Zhang P. (2008). The lipin protein family: dual roles in lipid biosynthesis and gene expression. *FEBS Letters*.

[13] Gaidhu M. P., Ceddia R. B. (2011). The role of adenosine monophosphate kinase in remodeling white adipose tissue metabolism. *Exercise and Sport Sciences Reviews*.

[14] Bijland S., Mancini S. J., Salt I. P. (2013). Role of AMP-activated protein kinase in adipose tissue metabolism and inflammation. *Clinical Science*.

[15] Daval M., Foufelle F., Ferre P. (2006). Functions of AMP-activated protein kinase in adipose tissue. *The Journal of Physiology*.

[16] Vila-Bedmar R., Lorenzo M., Fernandez-Veledo S. (2010). Adenosine 5’-Monophosphate-Activated protein kinase-mammalian target of rapamycin cross talk regulates brown adipocyte differentiation. *Endocrinology*.

[17] Seale P., Bjork B., Yang W. (2008). PRDM16 controls a brown fat/skeletal muscle switch. *Nature*.

[18] Wan Z., Root-Mccaig J., Castellani L., Kemp B. E., Steinberg G. R., Wright D. C. (2014). Evidence for the role of AMPK in regulating PGC-1 alpha expression and mitochondrial proteins in mouse epididymal adipose tissue. *Obesity*.

[19] Sung Y.-Y., Yoon T., Yang W.-K., Kim S. J., Kim H. K. (2011). inhibitory effects of Elsholtzia ciliata extract on fat accumulation in high-fat diet-induced obese mice. *Journal of Korean Society for Applied Biological Chemistry*.

[20] Annamalai S., Mohanam L., Alwin D., Prabhu V. (2016). Effect of combination therapy of melatonin and orlistat on high fat diet induced changes in lipid profiles and liver function parameters in serum of rats. *Obesity Medicine*.

[21] Miyashita K., Maeda H., Okada T., Abe M., Hosokawa M. (2010). Anti-obesity, and anti-diabetic effects of allenic carotenoid, fucoxanthin. *Agro Food Industry Hi-Tech*.

[22] Wu J., Boström P., Sparks L. M. (2012). Beige adipocytes are a distinct type of thermogenic fat cell in mouse and human. *Cell*.

[23] Hill J. O., Melanson E. L., Wyatt H. T. (2000). Dietary fat intake and regulation of energy balance: implications for Obesity. *The Journal of Nutrition*.

[24] Zha L. Y., Mao L. M., Lu X. C. (2011). Anti-inflammatory effect of soyasaponins through suppressing nitric oxide production in LPS-stimulated RAW 264.7 cells by attenuation of NF-*κ*B-mediated nitric oxide synthase expression. *Bioorganic & Medicinal Chemistry Letters*.

[25] Zhang W., Popovich D. G. (2010). Group B oleanane triterpenoid extract containing soyasaponins I and III from soy flour induces apoptosis in Hep-G2 cells. *J. Agric. Food Chem*.

[26] Takahashi S., Hori K., Shinbo M., Hiwatashi K., Gotoh T., Yamada S. (2008). Isolation of human renin inhibitor from soybean: soyasaponin I is the novel human renin inhibitor in soybean. *Bioscience, Biotechnology, and Biochemistry*.

[27] Itoh T., Furuichi Y. (2009). Lowering serum cholesterol level by feeding a 40% ethanol-eluted fraction from HP-20 resin treated with hot water extract of adzuki beans (_Vigna angularis_) to rats fed a high-fat cholesterol diet. *Nutrition*.

[28] Xu B. J., Chang S. K. C. (2012). Comparative study on antiproliferation properties and cellular antioxidant activities of commonly consumed food legumes against nine human cancer cell lines. *Food Chemistry*.

[29] Donà M., Dell’Aica I., Calabrese F. (2003). Neutrophil restraint by green tea: inhibition of inflammation, associated angiogenesis, and pulmonary fibrosis. *Journal of Immunology*.

[30] Ahmed S., Wang N., Lalonde M., Goldberg V. M., Haqqi T. M. (2004). Green tea polyphenol epigallocatechin-3-gallate (EGCG) differentially inhibits interleukin-1*β*-induced expression of matrix metalloproteinase-1 and -13 in human chondrocytes. *The Journal of Pharmacology and Experimental Therapeutics*.

[31] Sudano Roccaro A., Blanco A. R., Giuliano F., Rusciano D., Enea V. (2004). Epigallocatechin-gallate enhances the activity of tetracycline in staphylococci by inhibiting its efflux from bacterial cells. *Antimicrob Agents Chemother*.

[32] Oak M. H., El Bedoui J., Schini-Kerth V. B. (2005). Antiangiogenic properties of natural polyphenols from red wine andgreen tea. *The Journal of nutritional biochemistry*.

[33] Zhang Y. M., Rock C. O. (2004). Evaluation of Epigallocatechin Gallate and Related Plant Polyphenols as Inhibitors of the FabG and FabI Reductases of Bacterial Type II Fatty-acid Synthase∗. *The Journal of Biological Chemistry*.

[34] Weber J. M., Ruzindana-Umunyana A., Imbeault L., Sircar S. (2003). Inhibition of adenovirus infection and adenain by green tea catechins. *Antiviral Research*.

[35] Weinreb O., Mandel S., Amit T., Youdim M. B. (2004). Neurological mechanisms of green tea polyphenols in Alzheimer's and Parkinson's diseases. *The Journal of Nutritional Biochemistry*.

[36] Carrera-Lanestosa A., Moguel-Ordóñez Y., Segura-Campos M. (2017). Stevia rebaudianaBertoni: a natural alternative for treating diseases associated with metabolic syndrome. *Journal of Medicinal Food*.

[37] Park J. E., Kee H. J., Cha Y. S. (2010). Effect of Stevia rebaudianaBertoni leaf extract on antiobesity in C57BL/6J miceKorean. *Korean Journal of Food Science and Technology*.

[38] Ghaben A. L., Scherer P. E. (2019). Adipogenesis and metabolic health. *Nature Reviews. Molecular Cell Biology*.

[39] Cohen P., Levy J. D., Zhang Y. (2014). Ablation of PRDM16 and beige adipose causes metabolic dysfunction and a subcutaneous to visceral fat switch. *Cell*.

[40] Tung Y. C., Hsieh P. H., Pan M. H., Ho C. T. (2017). Cellular models for the evaluation of the antiobesity effect of selected phytochemicals from food and herbs. *Journal of Food and Drug Analysis*.

[41] Ahmad B., Serpell C. J., Fong I. L., Wong E. H. (2020). Molecular mechanisms of adipogenesis: the anti-adipogenic role of AMP-activated protein kinase. *Frontiers in molecular biosciences*.

[42] Ali A. T., Hochfeld W. E., Myburgh R., Pepper M. S. (2013). Adipocyte and adipogenesis. *European Journal of Cell Biology*.

[43] Haider N., Larose L. (2019). Harnessing adipogenesis to prevent obesity. *Adipocytes*.

[44] Lazar A. D., Dinescu S., Costache M. (2018). Adipose tissue engineering and adipogenesis – a review. *Reviews in Biological and Biomedical Sciences*.

[45] Khalilpourfarshbafi M., Gholami K., Murugan D. D., Abdul Sattar M. Z., Abdullah N. A. (2019). Differential effects of dietary flavonoids on adipogenesis. *European Journal of Nutrition*.

[46] Garten A., Schuster S., Kiess W. (2012). The insulin-like growth factors in adipogenesis and obesity. *Endocrinology and Metabolism Clinics*.

[47] Klemm D. J., Leitner J. W., Watson P. (2001). Insulin-induced Adipocyte Differentiation:. *The Journal of Biological Chemistry*.

[48] Viollet B., Mounier R., Leclerc J., Yazigi A., Foretz M., Andreelli F. (2007). L'AMPK, une nouvelle cible therapeutique pour le traitement des maladies metaboliques. *Diabetes & Metabolism*.

[49] Ejaz A., Wu D., Kwan P., Meydani M. (2009). Curcumin inhibits adipogenesis in 3T3-L1 adipocytes and angiogenesis and obesity in C57/BL mice. *The Journal of Nutrition*.

[50] Rosen E. D., Spiegelman B. M. (2001). PPAR*γ*: a Nuclear Regulator of Metabolism, Differentiation, and Cell Growth∗. *Journal of Biological Chemistry*.

[51] Cabrera C., Artacho R., Giménez R. (2006). Beneficial effects of green tea--a review. *Journal of the American College of Nutrition*.

[52] Matsui Y., Kobayashi K., Masuda H. (2009). Quantitative analysis of saponins in a tea-leaf extract and their antihypercholesterolemic activity. *Bioscience, Biotechnology, and Biochemistry*.

[53] Kaur K., Michael H., Arora S., Härkönen P., Kumar S. (2005). In vitro bioactivity-guided fractionation and characterization of polyphenolic inhibitory fractions from Acacia nilotica (L.) Willd. ex Del. *Journal of Ethnopharmacology*.

[54] Ren N., Chen L., Li B., Rankin G. O., Chen Y. C., Tu Y. (2020). Purified tea (Camellia sinensis (L.) Kuntze) flower saponins induce the p53-dependent intrinsic apoptosis of cisplatin-resistant ovarian cancer cells. *International Journal of Molecular Sciences*.

[55] Syam S., Bustamam A., Abdullah R. (2014). *β*-Mangostin induces p53-dependent G2/M cell cycle arrest and apoptosis through ROS mediated mitochondrial pathway and NfkB suppression in MCF-7 cells. *Journal of Functional Foods*.

[56] Kitagawa I., Hori K., Motozawa T., Murakami T., Yoshikawa M. (1998). Structures of new acylated oleanene-type triterpene oligoglycosides, theasaponins E1 and E2. From the seeds of tea plant, Camellia sinensis (L.) O. Kuntze. *Chemical and pharmaceutical bulletin*.

[57] Lee H. B., Kim E. K., Park S. J., Bang S. G., Kim T. G., Chung D. W. (2011). Isolation and anti-inflammatory effect of astragalin synthesized by enzymatic hydrolysis of tea seed extract. *Journal of the Science of Food and Agriculture*.

[58] Hasegawa T., Akutsu K., Kishi Y., Nakamura K. (2011). Constituents of the green tea seeds ofCamellia sinensis. *Natural Product Communications*.

[59] Musial C., Kuban-Jankowska A., Gorska-Ponikowska M. (2020). Beneficial properties of green tea catechins. *International journal of molecular sciences*.

[60] Lee Y. H., Jin B., Lee S. H. (2016). Herbal formula HT048 attenuates diet-induced obesity by improving hepatic lipid metabolism and insulin resistance in obese rats. *Molecules*.

[61] Bae M. G., Hwang-Bo J., Lee D. Y., Lee Y.-H., Chung I. S. (2021). Effects of 6,8-diprenylgenistein on VEGF-A-induced lymphangiogenesis and lymph node metastasis in an oral cancer sentinel lymph node animal model. *International Journal of Molecular Sciences*.

[62] Chaudhary N., Bhardwaj J., Hwang J. H. (2015). Anti-hyperlipidemic and fat pad lowering effect of standardized tea seed cake extract in mice fed high-fat and high-carbohydrate diet. *Biotechnology and Bioprocess Engineering*.

[63] Khan M. I., Shin J. H., Shin T. S., Kim M. Y., Cho N. J., Kim J. D. (2018). Anthocyanins from Cornus kousa ethanolic extract attenuate obesity in association with anti-angiogenic activities in 3T3-L1 cells by down-regulating adipogeneses and lipogenesis. *PLoS One*.

