# Supplementary material for: Pharmacological Approaches to Attenuate Inflammation and Obesity with Natural Products Formulations by Regulating the Associated Promoting Molecular Signaling Pathways
Source: Biomed Res Int. 2021 Nov 12;2021:2521273. doi: 10.1155/2021/2521273 (PMC8605410; doi:10.1155/2021/2521273)
Supplement: Supplementary 1 — File 1: compound isolation and purification process. [file 2521273.f1.pdf]

theasaponin E1 ($C_{59}H_{90}O_{27}$) and stevia fraction (stevioside ($C_{38}H_{60}O_{18}$) and rebaudioside A ($C_{44}H_{70}O_{23}$)) isolation

theasaponin E1 extraction process

Green tea (Camellia sinensis) seeds were collected from the Myungin Shin Gwang Su tea garden (Suncheon, Korea). The seeds were dried, de-hulled, and ground into powder. The powder (3 kg) was defatted with n-hexane (4 L) under sonication at 30°C for 5 h and then dried. The defatted seed powder was further extracted by refluxing with 70% ethanol at 60°C for 8 h. The resulting extract was filtered, concentrated using a rotatory vacuum evaporator (SB-100, Eyela), freeze dried, and weighed. The extract was again subjected to extraction with butanol and water mixture and concentrated with rotary rotatory evaporator. Saponins extraction from the crude extract was carried out by Non-polar macropours resins (D101). Resins were thoroughly washed 2 times with ethanol and then distal water. 10 g of the extract was dissolved in 30 ml double distal water and mixed with the washed resin and kept overnight at room temperature. 50 g of the extract was dissolved in 100 ml double distilled water and was passed through the resin column eluted first with 0.4 N NaOH followed by neutralization of the extract and resin mixture with HCL and again elution with eluted with 100% ethanol resulted in saponin rich mixture. This saponin mixture was then subjected to column chromatography using C18 column. First eluted with 10% MeOH to wash carbohydrates followed elution with 60% MeOH to wash out various acids and Finally with 100% MeOH to obtain the saponins mixture. Pure saponin (Theasaponin E1) was then isolated from this fraction by preparative high-performance liquid chromatography (HPLC) (Shimadzu Co., Kyoto, Japan) equipped with a photodiode array (PDA) detector. The fraction was separated on a Luna C-18(2) reverse phase column (250 mm x 21.2 mm, 15 µm; Phenomenex, Inc., Torrance, CA, USA) at 35 °C. Solvent A was methanol and solvent B was distilled water containing 0.1% formic acid. The non-linear gradient system used was initially A/B (74:26) to A/B (74.8:25.2) at 33.5 min to A/B (100:0) for 2 min and held A/B (100:0) for 10 min and then A/B (74:26) for 12 min. Components were detected at 210 nm. Flow rate 7 mL/min was used. Identification and determination of the isolated saponins were done by LC/TOF-MS and NMR

Extraction of sativoside and rebaudiosides fraction from stevia

Dried stevia leaves were ground into fine powder and extracted with 70% EtOH at 60°C for 6hrs by continuous reflux in heating mantle. The mixture was filtered using filter bags followed

by super jet filter and concentrated with a rotatory vacuum evaporator (SB-100, Eyela). The concentrated extract was collected as crude *Stevia rebaudiana* extract and dried by lyophilization. The crude extract was further extracted with nonpolar macropours resins. The mixture of the extract and resin was the loaded to a column of 500ml and eluted with 80 % EtOH resulted in a dark radish brown fraction. The resins were then washed twice with $2DH_2O$ and mixed with the fraction and incubated overnight in the column again then eluted with 100% ethanol resulted in the required fraction. In this process unwanted color compounds were separated and discarded, and brownish fraction was obtained. This fraction was dried and further fractionation and purification of the were done by column chromatography using C-18 column. First column was washed with 10% EtOH. 50 g of the fraction was dissolved in 100ml 100% EtOH and loaded to the C-18 Luna column. It was first eluted with 60% EtOH and we got one fraction and elution was continued with 100% EtOH and another fraction was obtained which is believed to contain the stevioside and rebaudiosides. This fraction was again loaded on the column and eluted with 100% EtOH. The resulted fraction was concentrated and was analyzed. The resulting fraction was concentrated and analyzed. Identification and determination of the isolated compounds were conducted by LC/TOF-MS and NMR. Rebaudioside A and stevioside with small fractions of rebaudiosides B and D was detected in the isolated purified fraction. The fraction was further purified from the traces of other compounds by eluting again with 100% EtOH using the C18-Luna col-umn. The purified final fraction contained stevioside and rebaudioside A (76.5% and 32.1%, respectively).

Identification and determination of the isolated compounds were done by LC/TOF-MS and NMR
