# Supplementary material for: Pharmacological Approaches to Attenuate Inflammation and Obesity with Natural Products Formulations by Regulating the Associated Promoting Molecular Signaling Pathways
Source: Biomed Res Int. 2021 Nov 12;2021:2521273. doi: 10.1155/2021/2521273 (PMC8605410; doi:10.1155/2021/2521273)

HPLC Conditions

Column: ZORBAX Extend C18 (2.1 * 150 mm, 5um, Agilent)

Column Temperature: 40℃

Eluents: A: 5mM ammonium formate,

B: 5mM ammonium formate/MeOH

Gradient:

Time (min)	A (%)	B (%)
0.0	90	10
2.0	90	10
5.0	20	80
12.0	0	100
14.0	0	100
15.0	90	10
20.0	90	10

Flow Rate: 200 μℓ/min

Injection volume: 10 μℓ

Mass spectrometer Condition

Ion Source Type: ESI (negative mode)

Spray Voltage: 3000

Sheath Gas Pressure: 40

Aux Gas Pressure: 10

Capillary Temperature: 300 ℃

Full scan: m/z 200-1300

SRM;

Compounds	Parent Ion [m/z]	Product Ion [m/z]	Collision Energy
Stevioside	803.22	641.26	45
RebaudiosideA	966.30	804.33	29

Table S1. Quantification of samples.

stevioside

Filename	RT [min]	Area			Acquired amount [ng/mL]			Average amount [ng/mL]	Calculated amount [ug/mL]	회석 배수
sample 1	8.54	6122118.28	6241433.74	6142647.38	852.83	869.98	855.79	859.53	85953.46	1.E+05
sample 2	8.56	4618518.92	2717348.39	2231974.38	636.72	363.46	293.70	431.29	4.31	1.E+01
sample 3	8.55	1920847.03	1827509.61	1815637.14	248.98	235.56	233.86	239.47	2394.66	1.E+04
sample 4	8.56	2201003.56	2125091.45	2194973.21	289.25	278.33	288.38	285.32	14.27	5.E+01
sample 5	8.57	1451556.44	1551502.87	1522879.57	181.53	195.89	191.78	189.73	1897.32	1.E+04
sample 6	8.56	762421.18	746549.09	758744.15	82.48	80.19	81.95	81.54	815.39	1.E+04

* Calculated amount = Average amount * 희석배수 / 1000 (단위변환)

rebaudioside_A

Filename	RT [min]	Area			Acquired amount [ng/mL]			Average amount [ng/mL]	*Calculated amount [ug/mL]	회석 배수
sample 1	8.53	1307894.66	1380927.16	1351713.48	340.458	360.507	352.487	351.15	35115.05	1.E+05
sample 2	8.55	906271.62	483803.83	404859.69	230.204	114.227	92.555	145.66	1.46	1.E+01
sample 3	8.54	369046.39	365746.33	368243.67	82.724	81.818	82.503	82.35	823.48	1.E+04
sample 4	8.55	448055.68	440812.16	395434.26	104.413	102.425	89.968	98.94	4.95	5.E+01
sample 5	8.54	3438384.81	3615710.53	3604970.68	925.323	974.003	971.055	956.79	956.79	1.E+03
sample 6	8.54	2314130.17	2445474.77	2418146.00	616.691	652.748	645.246	638.23	638.23	1.E+03

* Calculated amount = Average amount * 희석배수 / 1000 (단위변환)

Fig S1. Standard curves.

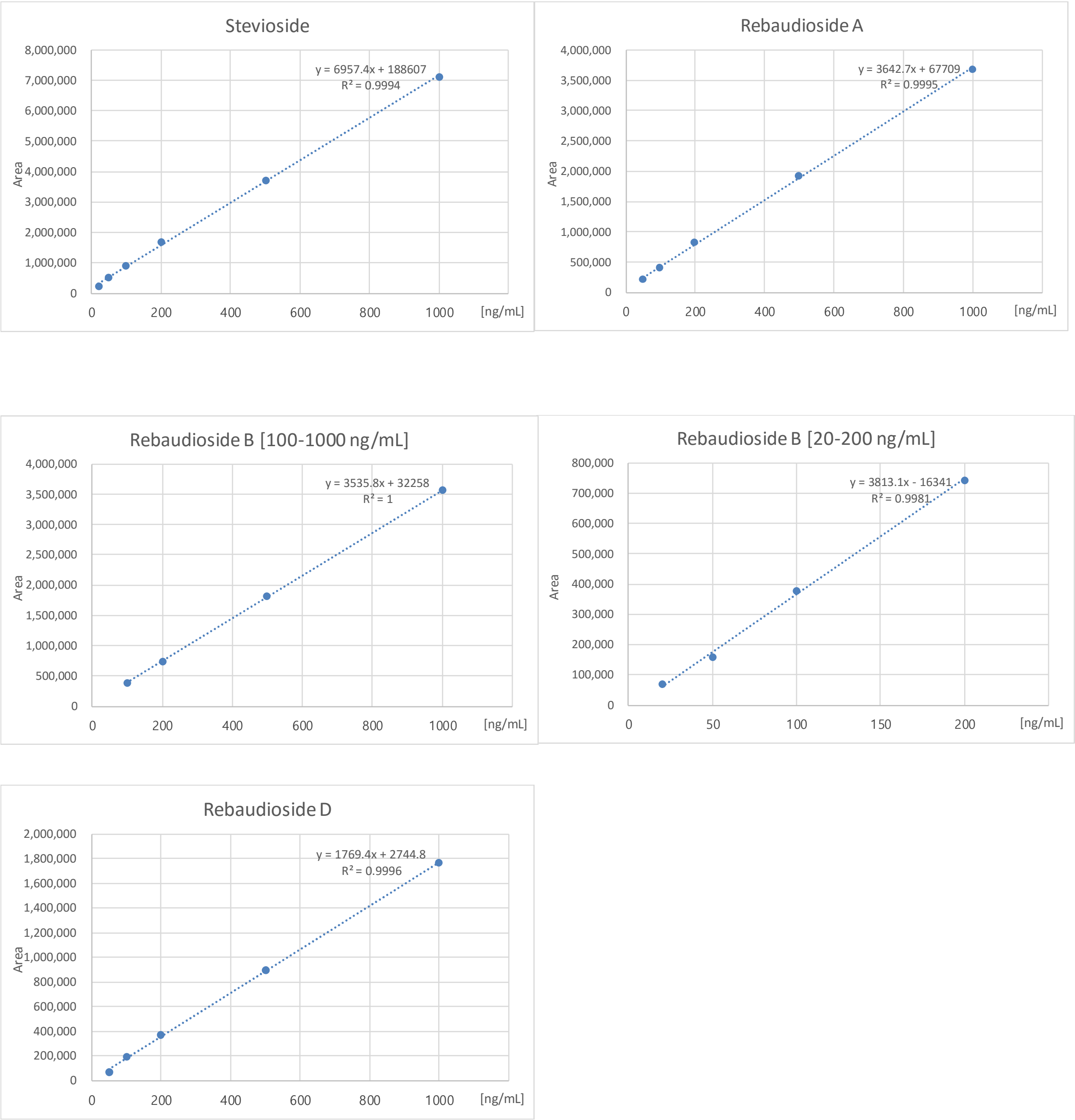

Fig S2. Chromatogramof stnadards, 500 ng/mL.

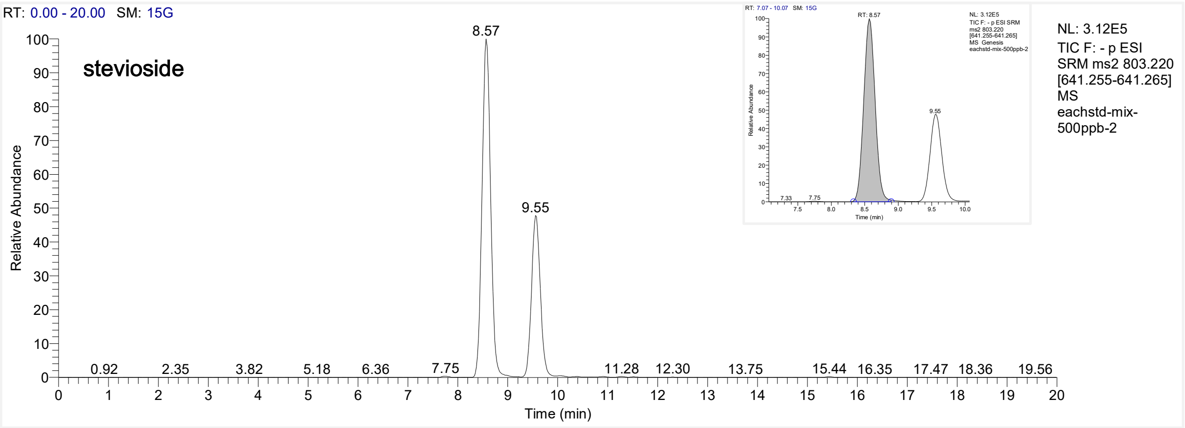

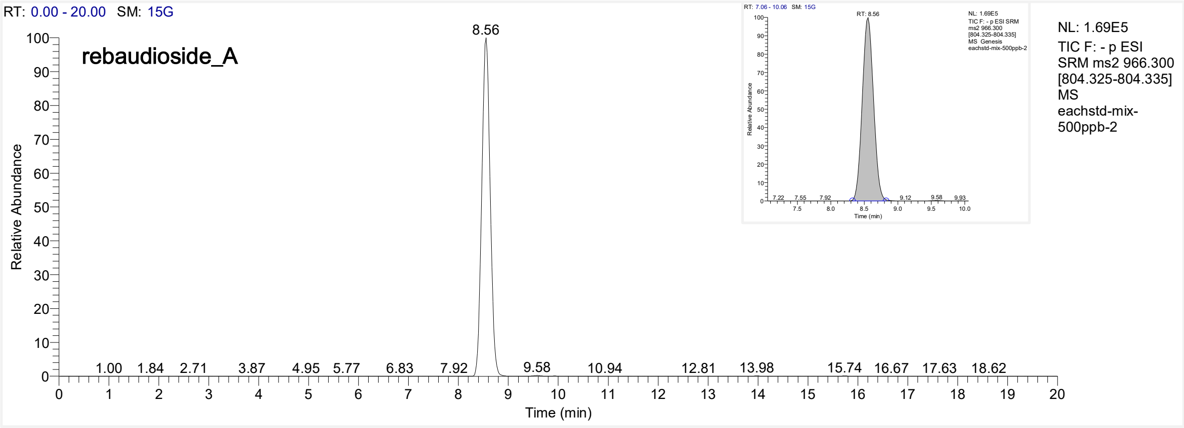

Fig S3. Chromatogram of sample

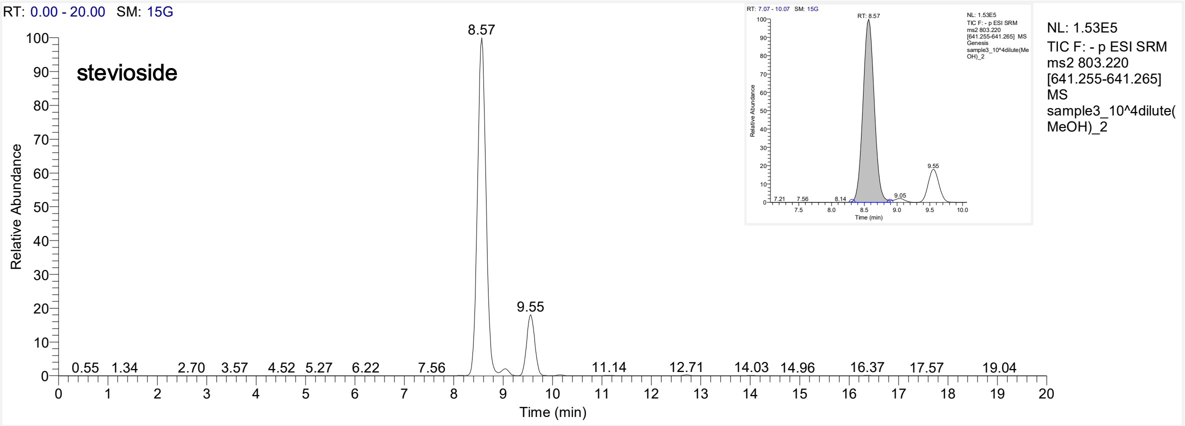

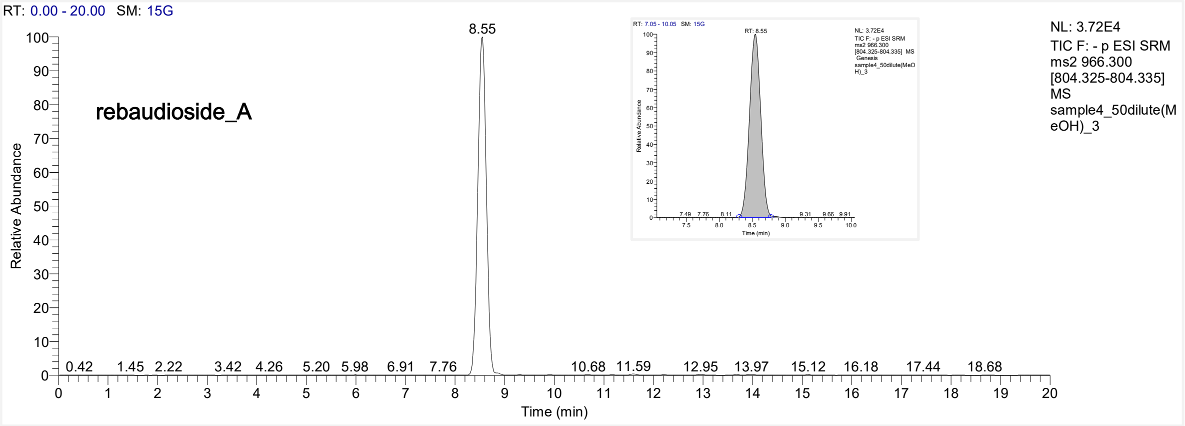

Fig S4. Chromatogram and MS spectrum of standardfull scan data, 1mg/mL

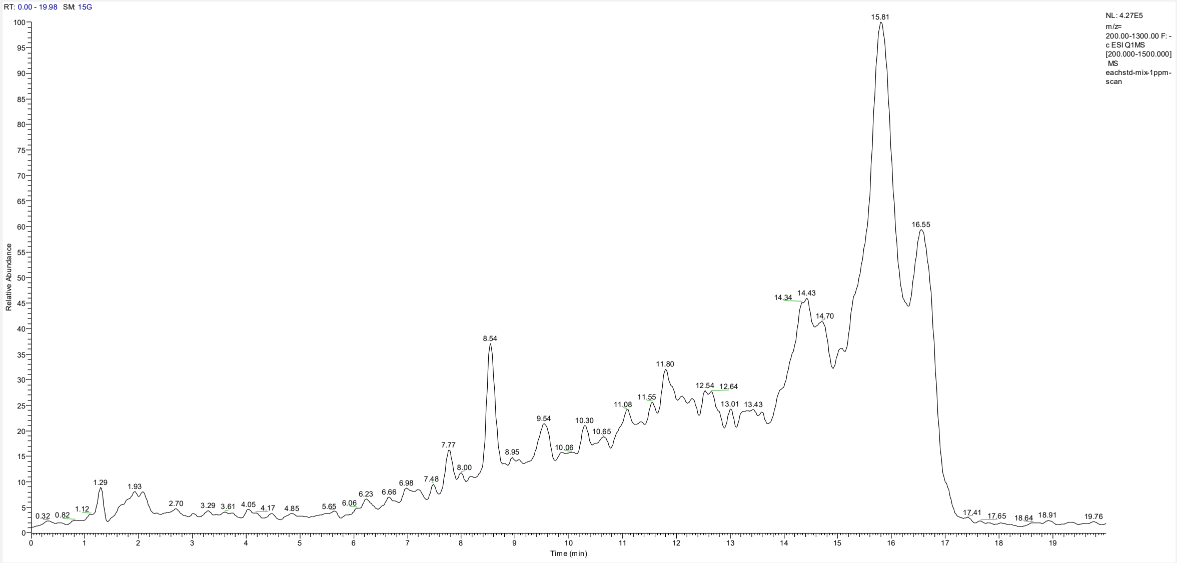

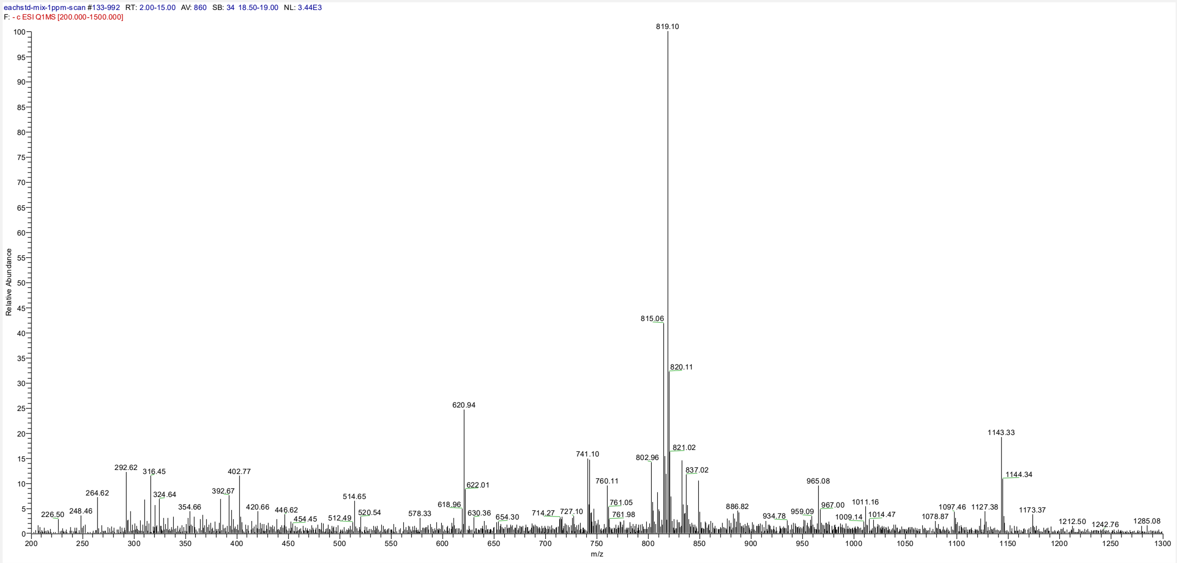

Fig S5. Chromatogram and MS spectrum of samplefull scan data.

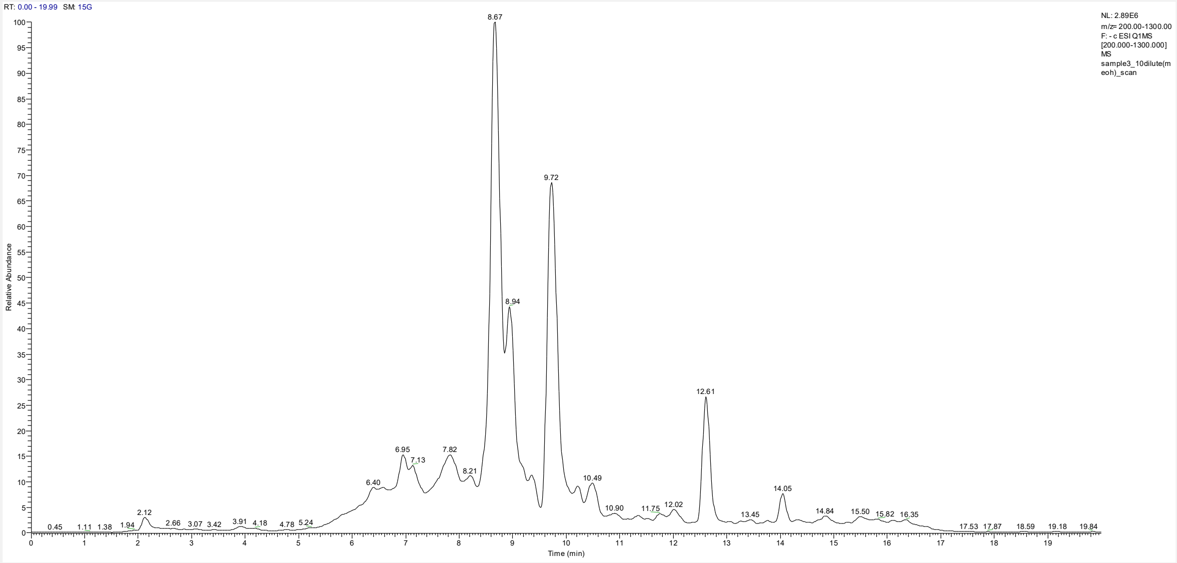

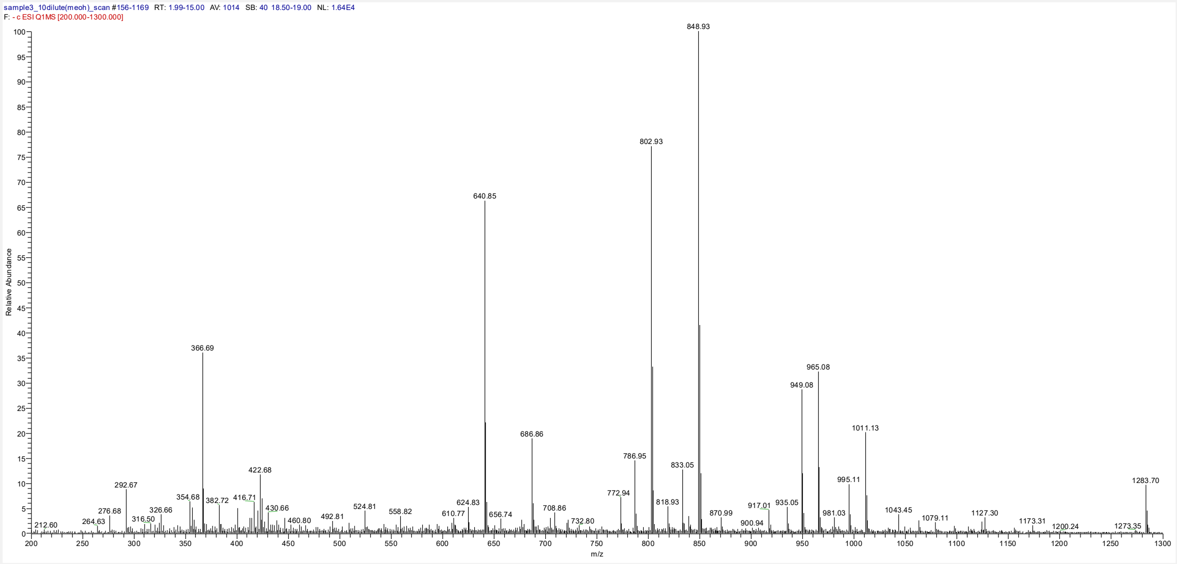

Supplement: Supplementary 2 — File 2: stevioside and rebaudioside A identification and quantification BY LC-QTOF-MS. [file 2521273.f2.pdf]
