# Supplementary material for: Pharmacological Approaches to Attenuate Inflammation and Obesity with Natural Products Formulations by Regulating the Associated Promoting Molecular Signaling Pathways
Source: Biomed Res Int. 2021 Nov 12;2021:2521273. doi: 10.1155/2021/2521273 (PMC8605410; doi:10.1155/2021/2521273)
Supplement: Supplementary 3 — File 3: stevioside and rebaudioside A structure elucidation by NMR. [file 2521273.f3.pdf]

Stevioside δ1H (PPM)	Stevioside δ13C (PPM)	Revaudioside A δ1H (PPM)	Revaudioside A δ13C (PPM)
0.7603	15.1829	0.7773	15.0779
0.7755	18.723	0.7817	18.6776
0.7802	20.0335	0.8683	19.8953
0.7934	21.2446	0.9048	21.2012
0.8697	28.1138	0.9161	28.1208
0.9018	35.6244	0.986	36.2344
0.9125	37.4337	0.9915	37.4312
0.9663	38.9811	1.0406	38.9402
0.972	40.0065	1.0556	40.0057
0.9851	40.1359	1.0594	40.0738
0.9908	41.0325	1.1409	41.0593
1.0042	42.0834	1.331	41.855
1.0094	43.2683	1.3495	43.1298
1.0387	43.5545	1.3584	43.1832
1.0563	46.933	1.3646	47.0106
1.1387	53.1678	1.434	53.2033
1.3229	56.5118	1.4499	56.4668
1.3421	60.5393	1.4696	60.5083
1.3548	60.719	1.489	60.9805
1.3582	61.0641	1.685	61.179
1.3753	69.556	1.7022	68.9136
1.3923	69.683	1.7191	69.5221
1.4624	70.3817	1.7706	70.0742
1.4662	72.5926	1.7883	70.2508
1.4798	75.3525	1.8094	72.5565
1.4868	76.0682	1.8584	73.6811
1.5104	76.2544	1.8678	74.5685
1.6927	76.287	1.8757	76.018
1.7047	76.9557	1.9236	76.4411
1.7116	77.0209	1.9424	76.523
1.7632	77.695	1.9703	76.6657
1.7797	82.6729	1.9954	76.9105
1.7926	84.696	2.0319	76.9558
1.7975	94.1975	2.0531	77.6325
1.8585	96.4148	2.0556	78.8937
1.8678	103.9851	2.0698	85.2025
1.9454	104.6765	2.0888	86.2345
1.9651	153.6076	2.105	94.1342
1.9896	175.7333	2.9602	96.6563
2.0215		2.9717	102.4859
2.0458		2.983	103.0699

2.0498	2.9942	103.9561
2.0586	3.0215	153.2071
2.0763	3.029	175.6123
2.1401	3.034	
2.1562	3.0418	
3.0163	3.0458	
3.027	3.053	
3.0406	3.0574	
3.0442	3.066	
3.0475	3.0713	
3.0515	3.0792	
3.057	3.1178	
3.0732	3.1218	
3.1333	3.1309	
3.1415	3.1391	
3.146	3.1442	
3.1544	3.1459	
3.1582	3.1518	
3.166	3.1557	
3.1747	3.1584	
3.1822	3.1639	
3.185	3.1716	
3.1916	3.1778	
3.196	3.1841	
3.1989	3.187	
3.2123	3.1905	
3.2251	3.1936	
3.2366	3.1979	
3.245	3.2008	
3.3656	3.2039	
3.3732	3.2271	
3.3778	3.2348	
3.4128	3.2397	
3.421	3.2473	
3.4294	3.3912	
3.4557	3.3994	
3.4642	3.4076	
3.4729	3.4137	
3.4791	3.4216	
3.4852	3.4296	
3.4869	3.4372	
3.4943	3.4452	
3.502	3.4534	

3.5646	3.462
3.5683	3.4708
3.5714	3.4761
3.6187	3.4887
3.6254	3.5
3.6332	3.5289
3.6403	3.541
3.6629	3.5535
3.6707	3.5984
3.6788	3.6066
3.6868	3.615
4.1956	3.6176
4.2036	3.6225
4.2118	3.6321
4.3493	3.6397
4.3604	3.6724
4.4483	3.6807
4.4594	3.6867
4.4791	3.6902
4.4871	3.6939
4.495	3.7002
4.6125	3.7031
4.6209	3.7074
4.6292	4.0897
4.7214	4.0979
4.8532	4.1061
4.8593	4.4154
4.9798	4.4267
4.9862	4.4599
4.9922	4.4709
5.0321	4.4842
5.2279	4.4922
5.2363	4.501
5.2481	4.5044
5.2598	4.5892
5.326	4.5968
5.3304	4.6044
5.6884	4.6264
5.6933	4.6376
	4.6799
	4.7385
	4.8955
	4.899

4.9022
4.972
4.9794
4.9861
5.0248
5.0328
5.0515
5.1014
5.1085
5.1377
5.1472
5.2276
5.2359
5.266
5.2777
5.6244
5.6317
