# Supplementary material for: Pharmacological Approaches to Attenuate Inflammation and Obesity with Natural Products Formulations by Regulating the Associated Promoting Molecular Signaling Pathways
Source: Biomed Res Int. 2021 Nov 12;2021:2521273. doi: 10.1155/2021/2521273 (PMC8605410; doi:10.1155/2021/2521273)
Supplement: Supplementary 4 — File 4: theasaponin E1 isolation and quantification BY LC-QTOF-MS. [file 2521273.f4.pdf]

SPECTRUM - MS
Acid-hydrolyzed-saponin.RAW
- c ESI sid=10.00 Q1MS [500.000-1500.000]
Scan #: 1397-4047
RT: 12.44-36.06
AV: 2651

m/z	Intensity	Relative
500.0736	0.4	0
500.741	1140.2	1.77
501.8029	716.4	1.11
502.8096	926.9	1.44
503.6625	366.6	0.57
504.5367	602.2	0.93
505.1422	554.4	0.86
506.0286	534.5	0.83
506.8238	596.9	0.92
507.5253	411	0.64
508.4515	716.7	1.11
509.1575	763.1	1.18
510.114	706.4	1.09
510.9851	751.4	1.16
511.7963	430.2	0.67
512.6928	767.6	1.19
513.224	653.2	1.01
514.1427	513.8	0.8
514.9738	1213.8	1.88
515.9548	653.2	1.01
516.8897	820	1.27
517.6254	368.6	0.57
518.5786	694.6	1.08
519.2082	623.6	0.97
520.154	809.4	1.25
520.9672	823.9	1.28
521.9173	535.4	0.83
522.9651	1715.3	2.66
523.6426	649.6	1.01
524.502	960.9	1.49
525.2253	741.4	1.15
526.3338	805.2	1.25
527.1133	1235.3	1.91
528.1584	777.4	1.2
529.0404	901.1	1.4
529.9582	558	0.86
530.9678	1231.4	1.91
532.0649	746.9	1.16
533.0484	1106	1.71
533.9666	601.4	0.93
535.0694	1602.8	2.48
536.2041	885.2	1.37

Percentage composition of the s

Theasaponin E1(44%)
Theasaponin E3 (15%)
Theasaponin C (10%)
Assamsaponin B (9%)
Theasaponin A1(5%)
Assamsaponin A (4%)
Theasaponin A3 (2%)

537.1655	755.1	1.17
538.2501	823.7	1.28
539.1274	1619.5	2.51
540.2134	929.9	1.44
541.0997	1350.2	2.09
541.7499	372.8	0.58
542.6727	1175.3	1.82
543.6776	770.1	1.19
544.3045	2.2	0
544.8793	1258.5	1.95
545.999	778.8	1.21
547.0239	880.5	1.36
548.1405	571.9	0.89
549.1602	4816.1	7.46
550.2074	1816.5	2.81
551.1345	3119.3	4.83
552.146	1227.5	1.9
552.9313	665.5	1.03
553.6632	408.5	0.63
554.5862	552.3	0.86
555.2847	441.7	0.68
556.1717	472.2	0.73
556.9276	779.8	1.21
557.9416	590.5	0.91
558.9058	1194.4	1.85
559.8465	666.6	1.03
560.8132	890.8	1.38
561.5296	465.8	0.72
562.4952	753.3	1.17
563.2993	646.9	1
563.9071	0.7	0
564.5348	861.8	1.34
565.4669	547.7	0.85
566.374	817.7	1.27
567.1923	666.4	1.03
568.1232	549.7	0.85
568.9767	868.1	1.34
569.8602	449	0.7
570.8249	937.3	1.45
571.6871	428.7	0.66
572.5221	731.5	1.13
573.2181	700.3	1.08
574.2488	847	1.31
575.1242	704.8	1.09
576.059	508.8	0.79
577.0042	858.7	1.33
577.8909	447.3	0.69
578.4245	0.4	0
579.0048	1696.4	2.63
580.1656	989.3	1.53
581.0408	1142.9	1.77
581.8597	493.2	0.76

582.6625	765.7	1.19
583.2863	667.4	1.03
584.3413	782.6	1.21
585.1449	894.7	1.39
586.1753	689.5	1.07
587.1265	1125	1.74
588.154	758.6	1.18
589.0495	774.7	1.2
590.0468	565.1	0.88
591.071	1787.9	2.77
592.1027	959.9	1.49
593.0959	1404.3	2.18
594.161	971.3	1.5
595.1155	1085.8	1.68
596.1421	668.1	1.04
597.1068	968.2	1.5
598.1869	753.2	1.17
599.0324	1183.3	1.83
599.9025	588	0.91
600.8424	894.9	1.39
601.5987	481.1	0.75
602.4593	637.6	0.99
603.1604	823.2	1.28
604.1529	630.8	0.98
605.0389	860.2	1.33
606.0062	611.9	0.95
606.9495	1017.4	1.58
607.7318	523.6	0.81
608.6442	801	1.24
609.3264	601.7	0.93
610.1751	621.8	0.96
610.9948	629.5	0.98
611.6785	363.4	0.56
612.5649	629.3	0.97
613.1808	612.4	0.95
614.069	547.4	0.85
615.013	917.6	1.42
615.9384	562.8	0.87
616.4733	3.3	0.01
617.0664	2548.6	3.95
617.7625	658.7	1.02
618.4039	947.5	1.47
619.1839	1028.5	1.59
619.9704	516.4	0.8
621.0931	3290.4	5.1
621.7391	4	0.01
622.2455	1587	2.46
623.1812	3378.2	5.23
623.7053	0.1	0
624.2221	1548.1	2.4
625.1199	1223.8	1.9
625.9452	607.5	0.94

626.9099	814.3	1.26
627.9236	540.4	0.84
628.9357	851	1.32
629.9975	653.4	1.01
631.1714	16608.3	25.73
631.6879	0	0
632.2422	5903.6	9.15
633.1901	10905.4	16.9
634.2369	4070.4	6.31
635.2101	1457.6	2.26
635.9342	482.4	0.75
636.7661	893.6	1.38
637.4263	515.3	0.8
638.2378	538.6	0.83
639.0924	813.3	1.26
640.1535	636	0.99
641.0792	630	0.98
642.0827	484.2	0.75
642.9754	691.1	1.07
643.8161	556.9	0.86
644.806	1295	2.01
645.6192	796.4	1.23
646.744	1479.3	2.29
647.3904	2.7	0
647.9384	1060.2	1.64
648.996	1054.1	1.63
649.9925	682.5	1.06
650.9309	1191.8	1.85
651.68	612.4	0.95
652.6997	1246.2	1.93
653.6315	901.3	1.4
654.7067	857.6	1.33
655.5592	515.2	0.8
656.5034	650.1	1.01
657.1848	627.8	0.97
658.054	523.2	0.81
659.0434	1364.3	2.11
659.9947	864.5	1.34
660.5721	0.1	0
661.0909	2820.3	4.37
662.0859	1600	2.48
662.9533	1531.8	2.37
663.5057	593.8	0.92
664.2809	758.3	1.17
665.0904	1724.4	2.67
666.0582	1020.3	1.58
667.0144	1574.2	2.44
667.9757	853.3	1.32
668.9448	1041.5	1.61
669.847	667.7	1.03
670.8759	1348.4	2.09
671.6786	662.3	1.03

672.6653	1092.1	1.69
673.2882	1881.4	2.91
674.0688	26.7	0.04
674.6392	2593.5	4.02
675.3098	2486.6	3.85
675.9619	4.9	0.01
676.5325	2217.4	3.44
677.3866	971.5	1.51
678.2897	789.3	1.22
679.1601	4640.7	7.19
680.1854	2207.5	3.42
681.0743	2390.9	3.7
682.0421	1144	1.77
682.8683	868.7	1.35
683.6073	565.4	0.88
684.2422	2.2	0
684.7976	1215.6	1.88
685.7055	711.3	1.1
686.763	967.8	1.5
687.3163	0.7	0
687.8356	1072.1	1.66
688.4592	1205	1.87
689.2388	1196	1.85
690.207	3150.7	4.88
691.1347	2376.2	3.68
692.1243	1123.1	1.74
693.0862	1003.4	1.55
694.0062	563.1	0.87
694.7937	823.9	1.28
695.3873	706.3	1.09
696.2355	680.3	1.05
697.0844	973.7	1.51
697.9712	635.9	0.99
699.0349	2603.5	4.03
699.9899	1291.5	2
700.6309	820	1.27
701.2183	1554.2	2.41
702.2473	6363.6	9.86
703.2016	3352.5	5.19
704.1042	1258.9	1.95
705.02	1408.4	2.18
706.0861	1001.5	1.55
707.0848	1752.5	2.72
708.0956	1077.9	1.67
709.0624	2126.6	3.29
709.9123	925.6	1.43
710.7114	1004.2	1.56
711.4447	729.2	1.13
712.4525	719	1.11
713.1584	1129.8	1.75
714.2202	3586.3	5.56
715.1527	2268.1	3.51

716.2893	36180.8	56.05
717.3215	15376.8	23.82
718.3594	4354	6.75
719.2461	1386.1	2.15
719.8627	553.2	0.86
720.4624	3.7	0.01
721.0778	3428.5	5.31
721.6177	0	0
722.1955	1651.9	2.56
723.1901	2199.5	3.41
723.7009	0.9	0
724.2409	1247.5	1.93
725.1605	5657.6	8.76
726.2208	2320.8	3.6
727.0958	1415.5	2.19
728.2753	9971.2	15.45
729.3113	7432.7	11.51
730.367	5605.3	8.68
731.2763	3145.9	4.87
732.0638	1389	2.15
732.8446	1218.8	1.89
733.7121	897.5	1.39
734.5738	1106.6	1.71
735.3758	721.8	1.12
736.2559	815.2	1.26
737.0364	977.4	1.51
737.9355	893.4	1.38
738.6357	5.9	0.01
739.1722	13135.2	20.35
739.7199	0.4	0
740.2794	4763.4	7.38
741.2249	1771.9	2.75
741.7277	0.3	0
742.2374	2874.2	4.45
743.1053	2538.1	3.93
744.0316	1249.4	1.94
745.1395	4481.1	6.94
745.6926	0.5	0
746.2001	2069.9	3.21
746.7137	0.4	0
747.2324	5780	8.95
748.304	3252.7	5.04
749.3127	1794.8	2.78
750.2806	1007.5	1.56
751.116	948.3	1.47
752.0675	1343.8	2.08
753.0399	1482	2.3
754.1164	1157.1	1.79
755.0987	1579.4	2.45
756.1565	1307.8	2.03
757.1402	1439	2.23
758.1621	1100.4	1.7

759.0956	2210.7	3.42
760.1391	1312.9	2.03
761.2587	42959.1	66.55
762.2342	23157.4	35.88
763.327	6187.4	9.59
764.3895	2203.6	3.41
765.3125	1288.8	2
766.3397	996.2	1.54
767.1264	2619	4.06
768.0355	1802.6	2.79
769.0924	3682.4	5.7
770.2746	2054.2	3.18
771.1949	1661.3	2.57
772.3051	1378.1	2.14
773.2363	1606.6	2.49
774.2527	2809.5	4.35
775.174	2607.8	4.04
776.2095	3600.7	5.58
777.1981	2377.1	3.68
778.2453	1708.1	2.65
779.2308	2455.5	3.8
780.2434	1396.6	2.16
781.1624	5071.7	7.86
782.2353	3006.4	4.66
783.2603	1940.2	3.01
784.2479	4336.1	6.72
785.2148	2739.3	4.24
786.2317	1557.1	2.41
787.0419	1354.1	2.1
787.7922	991.2	1.54
788.6861	1529	2.37
789.3236	1317.4	2.04
790.3102	2157.1	3.34
791.1983	10267.3	15.91
792.1998	5009.8	7.76
793.2094	3161.3	4.9
794.2987	1872.3	2.9
795.2422	4850.4	7.51
796.2187	2655.8	4.11
797.1199	8617.7	13.35
798.1855	4957.7	7.68
798.6967	0.7	0
799.2135	3986.2	6.18
800.2794	2270.1	3.52
801.3468	1790.4	2.77
802.4035	1642.6	2.54
803.2054	7962.9	12.34
804.2759	6527.7	10.11
805.2316	12864.8	19.93
806.2554	4942.8	7.66
807.1844	37736.7	58.46
808.2838	15592.9	24.16

809.3259	5651.3	8.76
810.4735	2505.8	3.88
811.1731	11030	17.09
812.3346	7239.7	11.22
813.2625	5933.6	9.19
814.2573	3166.4	4.91
815.19	1991.3	3.08
816.1189	2181.1	3.38
817.0278	2412.1	3.74
818.102	1673.4	2.59
819.0578	3946.7	6.11
819.8978	1785.4	2.77
820.7004	575.6	0.89
821.2198	64548.1	100
822.3177	27132.4	42.03
823.3293	8373.8	12.97
824.3249	3994.7	6.19
825.1777	2562.8	3.97
826.0757	1832.7	2.84
827.082	3104.5	4.81
827.6013	0.1	0
828.1592	1733	2.68
829.1468	6103.5	9.46
830.1846	4031.9	6.25
831.2337	2235	3.46
832.4471	2004.2	3.11
833.4587	1479.8	2.29
834.5124	1123.2	1.74
835.1586	3500.4	5.42
836.187	2622.2	4.06
837.1484	5727.7	8.87
838.2472	4214.5	6.53
839.2235	4440	6.88
840.1912	1845.7	2.86
840.9735	3311.1	5.13
841.5502	1589.3	2.46
842.4569	2639.1	4.09
843.2932	2179.4	3.38
844.2198	1683	2.61
845.0862	1864.4	2.89
845.9639	1814.8	2.81
846.9146	2282	3.54
847.9296	1492.2	2.31
848.564	8.2	0.01
849.1546	10753.5	16.66
850.2592	6078.9	9.42
851.1968	10115.6	15.67
852.2292	5282.7	8.18
853.1042	5916.7	9.17
853.9981	3032.9	4.7
855.0559	5184.8	8.03
856.0741	2491.6	3.86

857.021	2229	3.45
857.9295	1298.9	2.01
859.004	2923.3	4.53
860.0209	1708.6	2.65
860.9551	2120.1	3.28
861.8522	1281	1.98
862.6659	234.3	0.36
863.1941	20626.2	31.95
863.717	0.9	0
864.2744	10195.7	15.8
865.3144	5028.5	7.79
866.4824	2671.1	4.14
867.2063	4187.6	6.49
868.2302	3240.2	5.02
869.2534	2302.1	3.57
869.7649	0.2	0
870.3232	1583.5	2.45
871.1464	2542.6	3.94
872.1468	2050	3.18
873.1062	2996.2	4.64
874.0273	1636.8	2.54
875.0815	3856.1	5.97
876.1726	2438.4	3.78
877.1975	2660.9	4.12
878.2622	1788.7	2.77
879.1315	3095.7	4.8
880.2169	2822.5	4.37
881.1543	2394.1	3.71
882.0349	1531.7	2.37
882.8617	1604	2.48
883.6203	1013.3	1.57
884.5423	1546.6	2.4
885.4523	1295.2	2.01
886.5012	1416.4	2.19
887.1471	1331.8	2.06
888.0405	1161.8	1.8
888.5899	0.7	0
889.1419	7666.5	11.88
890.2018	3864.3	5.99
891.1536	2790.6	4.32
892.1857	1749.3	2.71
893.1694	2228.9	3.45
894.23	1707.8	2.65
894.7472	1.5	0
895.2829	1689.8	2.62
896.2875	1106.6	1.71
897.135	2371.6	3.67
898.1	1867.4	2.89
899.1217	1599.4	2.48
899.6224	0.2	0
900.144	946	1.47
900.9263	1321.4	2.05

901.5283	843.1	1.31
902.2725	1003.7	1.55
903.1278	1793.9	2.78
903.9628	903.9	1.4
904.7822	1575.1	2.44
905.3239	2016.8	3.12
906.2326	2322.6	3.6
907.0597	1548.3	2.4
907.9791	2359	3.65
908.9337	4003.9	6.2
910.0113	2476.1	3.84
911.0497	2042.3	3.16
911.9208	1008.3	1.56
912.7022	1072	1.66
913.4834	1118.2	1.73
914.5217	1237.9	1.92
915.3645	1013.7	1.57
916.2689	837.1	1.3
917.0567	1301.3	2.02
917.8316	902.1	1.4
918.5374	1130.1	1.75
919.2553	1875.9	2.91
920.1575	1459.9	2.26
920.9486	1616.4	2.5
921.5679	1014.2	1.57
922.322	1540.9	2.39
923.1246	1467.3	2.27
923.8405	925.5	1.43
924.6193	1172.5	1.82
925.3965	967.3	1.5
926.2617	945.9	1.47
927.1558	1271.1	1.97
928.0737	897.1	1.39
928.9662	1088.1	1.69
929.8849	872.6	1.35
930.4467	4.2	0.01
931.0602	3163.3	4.9
932.149	1927.2	2.99
933.0828	1483.7	2.3
933.9256	1088	1.69
934.8963	1641.9	2.54
935.7571	1000	1.55
936.7648	1335	2.07
937.6006	849	1.32
938.5641	1262.9	1.96
939.3404	1119.8	1.73
940.3054	1196.5	1.85
941.2016	1376.4	2.13
941.7096	0.1	0
942.2363	1074.9	1.67
943.1246	1227.9	1.9
944.031	951.2	1.47

944.9433	1168.3	1.81
945.7819	790.4	1.22
946.2976	0.6	0
946.8106	1499.8	2.32
947.6984	1325.6	2.05
948.7082	1402.7	2.17
949.6047	1166.7	1.81
950.1425	0	0
950.7	1523.9	2.36
951.5129	944.2	1.46
952.4017	1236.9	1.92
953.3634	1148.9	1.78
954.4218	1204.1	1.87
955.2581	1000.3	1.55
956.1487	794	1.23
957.0962	1498.9	2.32
958.1223	1141.2	1.77
959.0609	1245.1	1.93
959.888	832.2	1.29
960.8574	1172.1	1.82
961.819	893.8	1.38
962.8481	1134.8	1.76
963.7751	843.7	1.31
964.8789	1436.8	2.23
965.9471	1142.6	1.77
967.016	1353.2	2.1
968.0275	948.3	1.47
968.9982	1183.3	1.83
969.9738	833.3	1.29
970.8875	873.1	1.35
971.7777	811.4	1.26
972.9213	1403	2.17
973.8954	827.1	1.28
974.7574	969.4	1.5
975.5331	691.4	1.07
976.213	755.2	1.17
977.0483	1066.6	1.65
977.9271	862.4	1.34
978.8707	928.9	1.44
979.7399	747.4	1.16
980.7591	983.6	1.52
981.7657	929.1	1.44
982.8466	1294.5	2.01
983.7918	942.6	1.46
984.666	837.9	1.3
985.3523	690.7	1.07
986.3193	862.9	1.34
987.2466	1060.6	1.64
988.2732	909.6	1.41
989.0932	976.2	1.51
990.1196	1024.5	1.59
991.1184	1172.4	1.82

992.1055	865.8	1.34
993.0862	1029.7	1.6
994.0745	880.7	1.36
995.0462	1024	1.59
995.9921	730	1.13
996.8657	780.9	1.21
997.6835	636.8	0.99
998.4939	638.3	0.99
999.2223	911.6	1.41
1000.053	727.6	1.13
1000.874	826.2	1.28
1001.547	630.3	0.98
1002.324	654.9	1.01
1003.054	699.4	1.08
1003.786	528.9	0.82
1004.553	805.4	1.25
1005.412	950.2	1.47
1006.325	777.4	1.2
1007.124	854.9	1.32
1007.921	731.9	1.13
1008.86	1041.6	1.61
1009.948	959.7	1.49
1010.99	1093.8	1.69
1011.528	1.3	0
1012.029	935.9	1.45
1013	977.6	1.51
1014.016	873.5	1.35
1015.039	998.3	1.55
1015.985	640.8	0.99
1016.866	864.5	1.34
1017.701	652.5	1.01
1018.519	635.6	0.98
1019.294	814.4	1.26
1020.014	768.1	1.19
1020.766	646.1	1
1021.487	649.4	1.01
1022.349	582.2	0.9
1023.293	814	1.26
1024.28	761.3	1.18
1025.177	782	1.21
1026.164	885.3	1.37
1027.14	959.4	1.49
1028.084	712.5	1.1
1028.989	716.3	1.11
1029.891	656.3	1.02
1030.841	909.4	1.41
1031.867	821.4	1.27
1032.89	825.9	1.28
1033.758	658.6	1.02
1034.747	934.7	1.45
1035.541	688.5	1.07
1036.424	699.1	1.08

1037.235	572.9	0.89
1038.023	522.7	0.81
1038.945	839.6	1.3
1039.951	713.4	1.11
1040.964	757.2	1.17
1041.898	702.3	1.09
1042.707	634	0.98
1043.429	552.8	0.86
1044.227	576	0.89
1044.977	703.7	1.09
1045.661	410.8	0.64
1046.426	514.6	0.8
1047.145	587.5	0.91
1047.922	540	0.84
1048.699	598.4	0.93
1049.467	720.3	1.12
1050.182	589.4	0.91
1051.011	627.8	0.97
1051.75	488.2	0.76
1052.581	522	0.81
1053.283	548	0.85
1054.086	587	0.91
1054.918	721.7	1.12
1055.722	755.6	1.17
1056.685	788.8	1.22
1057.648	702	1.09
1058.642	701.3	1.09
1059.45	581.2	0.9
1060.299	580.3	0.9
1061.161	651.3	1.01
1062.143	768.5	1.19
1063.182	711.2	1.1
1064.226	832	1.29
1065.279	818.3	1.27
1066.205	474	0.73
1066.914	463.7	0.72
1067.736	615.2	0.95
1068.731	641.3	0.99
1069.519	573.2	0.89
1070.427	769.3	1.19
1071.397	682.9	1.06
1072.419	649.6	1.01
1073.273	815.9	1.26
1074.288	633.2	0.98
1075.273	587.4	0.91
1076.203	539.6	0.84
1076.991	484.4	0.75
1077.699	550.8	0.85
1078.555	630.2	0.98
1079.389	540.8	0.84
1080.342	680.2	1.05
1081.38	635.5	0.98

1082.296	596.2	0.92
1083.12	519.6	0.8
1083.905	495.4	0.77
1084.696	538.5	0.83
1085.418	486.9	0.75
1086.183	570.6	0.88
1087.007	582.4	0.9
1087.841	565.2	0.88
1088.742	579.2	0.9
**1089.767**	606.7	0.94
1090.773	566.9	0.88
1091.528	547.8	0.85
1092.266	528.5	0.82
1093.222	955.9	1.48
1094.148	412.4	0.64
1094.77	570.9	0.88
1095.709	650.7	1.01
1096.626	546.3	0.85
1097.465	531.7	0.82
1098.414	616.5	0.96
1099.35	594.1	0.92
1100.2	428.7	0.66
1100.993	537.9	0.83
1101.892	461.1	0.71
1102.828	608.8	0.94
1103.65	576	0.89
1104.472	486.9	0.75
1105.194	502.7	0.78
1106.142	616.7	0.96
1107.198	798	1.24
1108.189	681.7	1.06
1109.15	543.2	0.84
1109.983	420.4	0.65
1110.847	512	0.79
1111.64	454.4	0.7
1112.426	520.4	0.81
1113.275	602.5	0.93
1114.195	417.3	0.65
1115.072	466.8	0.72
1115.758	506	0.78
1116.552	582.9	0.9
1117.47	531.2	0.82
1118.361	483.4	0.75
1119.337	540	0.84
1120.325	538.6	0.83
1121.196	545.6	0.85
1121.918	470	0.73
1122.676	413.7	0.64
1123.543	378.2	0.59
1124.484	520.8	0.81
1125.534	601.6	0.93
1126.478	645	1

1127.414	532.8	0.83	
1128.217	444.4	0.69	
1129.082	454.6	0.7	
1129.936	461.3	0.71	
1130.707	437.3	0.68	
1131.562	466.5	0.72	
1132.276	407.9	0.63	
1133.214	680.2	1.05	
1134.174	618	0.96	
1134.994	481.3	0.75	
1135.796	484.7	0.75	
1136.821	589.3	0.91	
1137.437	1.7	0	
1137.95	680.3	1.05	
1138.645	0	0	
1139.166	601.3	0.93	
1139.772	5.6	0.01	
1140.307	520	0.81	
1141.412	701.4	1.09	
1142.138	11.4	0.02	
1142.694	2826.5	4.38	
1143.648	1912.9	2.96	
1144.196	0.1	0	
1144.747	844.9	1.31	
1145.806	628.3	0.97	
1146.975	719.2	1.11	
1147.522	2.7	0	
1148.216	579.8	0.9	
1148.928	0.6	0	
1149.485	935.2	1.45	
1150.535	673.2	1.04	
1151.41	518.3	0.8	
1152.317	488.8	0.76	
1152.981	3	0	
1153.548	1226.4	1.9	
1154.051	0	0	
1154.688	874.1	1.35	
1155.256	0.9	0	
1155.775	641.9	0.99	
1156.826	629.4	0.98	
1157.67	721.1	1.12	
1158.491	492.2	0.76	
**1159.31**	795.5	1.23	desacyl-theasaponin F
1160.247	425.1	0.66	
1161.17	588	0.91	
1162.057	383.4	0.59	
1162.75	424.9	0.66	
1163.497	461.7	0.72	
1164.172	332.9	0.52	
1164.939	567.9	0.88	
1165.636	440.8	0.68	
1166.494	381.3	0.59	

1167.259	376.1	0.58		
1168.071	374.6	0.58		
1168.855	399.9	0.62		
1169.66	578.8	0.9		
1170.777	703	1.09		
**1171.709**	**776**	**1.2**	**$C_{57}H_{88}O_{25}$**	**Assamsaponin A**
1172.615	590.6	0.92		
1173.462	487.4	0.76		
1174.366	515.2	0.8		
1175.341	534.2	0.83		
1176.394	722.9	1.12		
1176.945	1.6	0		
1177.595	631.2	0.98		
1178.547	481.3	0.75		
1179.482	536.8	0.83		
1180.369	470.6	0.73		
1181.212	428.4	0.66		
1182.072	442.1	0.68		
1182.763	387.2	0.6		
1183.61	565.5	0.88		
1184.737	686.9	1.06		
1185.777	535.3	0.83		
1186.762	708.2	1.1		
**1187.644**	**611.4**	**0.95**	**$C_{57}H_{88}O_{26}$**	**Theasaponin E3**
1188.365	377.8	0.59		
**1189.03**	**403.2**	**0.62**	**$C_{57}H_{90}O_{26}$**	**Theasaponin A_1**
**1189.833**	**424.7**	**0.66**	**$C_{57}H_{90}O_{26}$**	**Theasaponin A_1**
1190.708	477.6	0.74		
1191.734	742.6	1.15		
1192.784	683.9	1.06		
1193.94	624.4	0.97		
1195.016	544.5	0.84		
1195.892	337.7	0.52		
1196.616	393.5	0.61		
1197.387	490.8	0.76		
1198.297	485.7	0.75		
1199.349	612.3	0.95		
1200.228	542.2	0.84		
1200.977	597.1	0.93		
1201.854	623.9	0.97		
1202.78	421.6	0.65		
1203.706	413.3	0.64		
1204.597	790.7	1.22		
1205.449	844.5	1.31		
1206.265	553.9	0.86		
1207.124	547.3	0.85		
1207.919	459.6	0.71		
1208.764	512.9	0.79		
1209.805	1721.2	2.67		
1210.374	0.9	0		

1210.877	1152	1.78		
**1211.876**	544.5	0.84	$C57H88O26$	
1212.82	502.4	0.78		
1213.708	574.6	0.89		
1214.652	441.8	0.68		
**1215.577**	471.6	0.73	$C57H88O25$	Assamsaponin E
1216.546	390.1	0.6	$C59H92O26$	
**1217.485**	897.5	1.39	$C_{58}H_{90}O_{27}$	Theasaponin F_1
1218.281	454.2	0.7		
1218.926	649.2	1.01		
1219.798	506	0.78		
1220.713	491.7	0.76		
1221.641	700.1	1.08		
1222.509	703.1	1.09		
1223.442	528.3	0.82		
1224.354	433	0.67		
1225.153	381	0.59		
1226.012	422.8	0.66		
1226.779	317.7	0.49		
1227.536	359.8	0.56		
1228.343	292.8	0.45		
**1229.08**	445.7	0.69		Theasaponin E4
**1229.664**	470	0.73		Theasaponin E4
**1230.611**	555.7	0.86	$C59H90O27$	**Theasaponin E1**
**1231.624**	680.4	1.05	$C_{59}H_{92}O_{27}$	Theasaponin A_2
1232.531	361.9	0.56	$C59H92O27$	Assamsaponin D
1233.323	391.4	0.61		
1234.134	486.3	0.75		
1235.065	540.7	0.84		
1235.996	444.7	0.69		
1236.9	416.4	0.65		
1237.899	478.6	0.74		
1238.822	477.1	0.74		
1239.759	544.1	0.84		
1240.593	300.7	0.47		
1241.191	272.3	0.42		
1241.926	306.3	0.47		
1242.799	386.3	0.6		
1243.314	0	0		
1243.815	701.7	1.09		
1244.832	545.1	0.84		
1245.802	492.5	0.76		
1246.727	371.6	0.58		
1247.655	540.1	0.84		
1248.668	396.8	0.61		
1249.625	448.2	0.69		
1250.564	375.2	0.58		
1251.536	508.7	0.79		
1252.465	420.1	0.65		

1253.362	416.1	0.64	$C_{59}H_{90}O_{27}$	
1254.397	437.1	0.68		
1255.486	452.4	0.7		
1256.507	361.3	0.56		
1257.411	326	0.5		
1258.307	368.6	0.57		
1259.271	446.2	0.69	$C_{60}H_{92}O_{28}$	Theasaponins F_2
1260.308	437	0.68		
1260.914	2.7	0		
1261.44	557.6	0.86		
1262.475	566.9	0.88		
1263.526	449.6	0.7		
1264.595	441.5	0.68		
1265.516	469.1	0.73		
1266.56	505.7	0.78		
1267.675	454.5	0.7		
1268.655	334.6	0.52		
1269.516	363	0.56		
1270.457	330.8	0.51		
**1271.424**	**435.3**	**0.67**	**$C_{61}H_{92}O_{28}$**	**Assamsaponin B**
1272.42	345.1	0.53		
1273.25	451.5	0.7	$C_{61}H_{94}O_{28}$	theasaponin A_3
1273.835	0.5	0	$C_{61}H_{94}O_{28}$	theasaponin A_3
1274.353	525.9	0.81		
1275.505	593.3	0.92		
1276.472	406.3	0.63		
1277.397	480	0.74		
1278.37	445.1	0.69		
1279.342	414.6	0.64		
1280.412	372.9	0.58		
1281.372	367.4	0.57		
1282.303	292.2	0.45		
1283.17	320.2	0.5		
1283.878	190.3	0.29		
1284.714	302.9	0.47		
1285.601	315.5	0.49		
1286.31	323.5	0.5		
1287.233	397.1	0.62		
1288.227	357.5	0.55		
1289.093	371.9	0.58		
1289.907	388.5	0.6		
1290.871	393.1	0.61		
1291.818	313.1	0.49		
1292.687	333.9	0.52		
1293.548	296.2	0.46		
1294.487	327.3	0.51		
1295.475	433.9	0.67	$C_{61}H_{92}O_{28}$	
1296.428	344.2	0.53		
1297.399	366.1	0.57		
1298.588	464.2	0.72		

1299.113	0	0
1299.624	335.1	0.52
1300.558	415.4	0.64
1301.617	372.9	0.58
1302.788	646.5	1
1303.804	412.9	0.64
1304.356	0.8	0
1304.9	541.5	0.84
1305.873	486.9	0.75
1306.864	362	0.56
1307.603	302.2	0.47
1308.47	289.4	0.45
1309.192	276.8	0.43
1310.046	284.2	0.44
1310.937	662.9	1.03
1311.87	330.5	0.51
1312.647	210.1	0.33
1313.357	202.7	0.31
1313.994	173.6	0.27
1314.746	292.3	0.45
1315.589	260.7	0.4
1316.463	278.6	0.43
1317.353	379.4	0.59
1317.858	0	0
1318.362	463.3	0.72
1319.359	312.7	0.48
1320.228	277.2	0.43
1321.279	298.3	0.46
1322.17	181.3	0.28
1322.744	156.7	0.24
1323.519	200.9	0.31
1324.307	249.7	0.39
1325.061	239.2	0.37
1325.955	260.3	0.4
1326.923	266.2	0.41
1327.796	242.1	0.38
1328.737	260.1	0.4
1329.607	291.7	0.45
1330.359	243.4	0.38
1331.282	291.2	0.45
1332.14	243.1	0.38
1332.92	236.1	0.37
1333.674	248.4	0.38
1334.527	248.1	0.38
1335.494	337.7	0.52
1336.42	296.1	0.46
1337.474	347.6	0.54
1338.555	305.7	0.47
1339.574	233.1	0.36
1340.645	224	0.35
1341.578	242.8	0.38
1342.339	182.9	0.28

1343.088	213.9	0.33
1343.918	242.4	0.38
1344.767	244.7	0.38
1345.508	201	0.31
1346.222	229.6	0.36
1347.281	451.8	0.7
1348.33	332.5	0.52
1349.363	314.9	0.49
1350.179	206.7	0.32
1350.998	262.6	0.41
1351.849	273.9	0.42
1352.83	301.1	0.47
1353.747	250.1	0.39
1354.64	197	0.31
1355.525	196.5	0.3
1356.474	234.8	0.36
1357.338	224.7	0.35
1358.229	262	0.41
1359.145	230.2	0.36
1359.993	266.6	0.41
1361.03	307.6	0.48
1361.558	5.8	0.01
1362.065	248	0.38
1363.116	238.4	0.37
1364.232	258.5	0.4
1365.229	273.8	0.42
1365.944	6.8	0.01
1366.54	356.2	0.55
1367.506	297.4	0.46
1368.343	139.3	0.22
1368.894	197.3	0.31
1369.592	175.5	0.27
1370.319	154.8	0.24
1371.051	159.7	0.25
1371.812	272.3	0.42
1372.691	171.2	0.27
1373.549	190.6	0.3
1374.413	191.2	0.3
1375.342	257.3	0.4
1376.221	186.4	0.29
1377.38	343.6	0.53
1378.316	179.5	0.28
1379.316	248.8	0.39
1380.353	195.8	0.3
1381.306	228.3	0.35
1382.412	238.4	0.37
1383.426	242.7	0.38
1384.43	200.3	0.31
1385.391	185.2	0.29
1386.339	234.1	0.36
1387.354	365.4	0.57
1388.232	172.6	0.27

1388.985	151.7	0.23
1389.627	154	0.24
1390.561	270.6	0.42
1391.593	240.9	0.37
1392.441	196.5	0.3
1393.315	237.8	0.37
1394.241	251.6	0.39
1395.176	209.8	0.33
1395.94	128.2	0.2
1396.814	171.2	0.27
1397.482	172.3	0.27
1398.291	158.9	0.25
1399.265	222.6	0.34
1400.193	196.5	0.3
1401.255	238.2	0.37
1402.25	171.2	0.27
1403.272	241.4	0.37
1404.471	247	0.38
1405.553	215.9	0.33
1406.502	195.5	0.3
1407.39	185.1	0.29
1408.112	167.3	0.26
1408.773	213.8	0.33
1409.718	312.3	0.48
1410.563	312.9	0.48
1411.318	181.5	0.28
1412.102	206.2	0.32
1412.834	125.2	0.19
1413.724	194	0.3
1414.269	0	0
1414.812	196.4	0.3
1415.869	205.2	0.32
1416.752	150.5	0.23
1417.693	148.1	0.23
1418.556	204.8	0.32
1419.472	241.9	0.37
1420.444	279.5	0.43
1421.454	246.6	0.38
1422.2	162.6	0.25
1422.997	158.4	0.25
1423.748	208.8	0.32
1424.508	122.4	0.19
1425.194	202	0.31
1425.88	156.7	0.24
1426.69	150.9	0.23
1427.765	233.2	0.36
1428.686	165.9	0.26
1429.412	150.1	0.23
1430.327	172.8	0.27
1431.242	241	0.37
1432.241	234.3	0.36
1433.259	272.8	0.42

1433.969	388.9	0.6
1434.822	352.8	0.55
1435.563	229.9	0.36
1436.144	149.1	0.23
1436.811	214.4	0.33
1437.716	214.7	0.33
1438.61	183.9	0.28
1439.616	222.3	0.34
1440.17	0.6	0
1440.682	237.8	0.37
1441.483	221.8	0.34
1442.436	183.2	0.28
1443.222	183	0.28
1444.034	184.2	0.29
1444.87	177.3	0.27
1445.76	247.9	0.38
1446.526	276.8	0.43
1447.537	272.1	0.42
1448.69	270.1	0.42
1449.257	0	0
1449.832	271.9	0.42
1450.832	253.2	0.39
1451.861	264.1	0.41
1452.648	173	0.27
1453.572	263.3	0.41
1454.57	199.9	0.31
1455.566	177.1	0.27
1456.479	155.2	0.24
1457.552	285	0.44
1458.473	145.6	0.23
1459.413	154.3	0.24
1460.226	199.2	0.31
1460.999	135.1	0.21
1461.909	185	0.29
1462.895	179.6	0.28
1463.685	129.2	0.2
1464.654	210	0.33
1465.572	179.6	0.28
1466.403	158.3	0.25
1467.293	127.2	0.2
1467.945	170.3	0.26
1468.808	138.6	0.21
1469.773	194.5	0.3
1470.652	120.5	0.19
1471.405	137.6	0.21
1472.096	118.4	0.18
1472.895	123.4	0.19
1473.743	155.5	0.24
1474.565	138.4	0.21
1475.501	148.2	0.23
1476.438	214.3	0.33
1477.229	191.2	0.3

1478.164	224.2	0.35
1479.079	364.4	0.56
1479.929	318.7	0.49
1480.815	192.4	0.3
1481.659	212.1	0.33
1482.464	135.7	0.21
1483.236	122.5	0.19
1484.221	155.7	0.24
1485.201	195.4	0.3
1486.106	143.7	0.22
1487.157	159.9	0.25
1488.197	159.4	0.25
1489.029	144.3	0.22
1489.756	147.4	0.23
1490.678	139.4	0.22
1491.243	3.4	0.01
1491.798	402.9	0.62
1492.394	0.8	0
1493.004	485.2	0.75
1494.023	286.1	0.44
1494.961	260.4	0.4
1495.773	178.9	0.28
1496.625	151.5	0.23
1497.532	128.6	0.2
1498.439	138.9	0.22
1499.444	109.1	0.17

aponins mixture

SPECTRUM - MS
tea_saponin.raw
- c ESI sid=10.00 Q1MS [500.000-1500.000]
Scan #: 2202-3114
RT: 19.62-27.74
AV: 913

m/z	Intensity	Relative
500.4942	276.2	0.23
501.1507	456.6	0.38
502.1676	286.9	0.24
503.1431	303.3	0.25
503.8186	40.2	0.03
504.4393	170.8	0.14
505.1883	195.2	0.16
506.3137	209.5	0.17
507.0981	163	0.13
507.8938	137.1	0.11
508.6771	220.3	0.18
509.3122	162.9	0.13
510.2527	228.1	0.19
511.2105	237.3	0.2
511.7611	5.8	0
512.3113	157.3	0.13
512.9642	246.6	0.2
513.7208	143.5	0.12
514.7232	271.5	0.22
515.3822	193.8	0.16
516.3614	45.9	0.04
516.9465	403.5	0.33
517.9933	261.9	0.22
519.1251	260.2	0.21
520.2141	190.2	0.16
521.0478	293.8	0.24
522.0326	143.6	0.12
523.028	463.7	0.38
523.5554	1.6	0
524.1225	297.7	0.25
525.086	266.2	0.22
525.9468	109.9	0.09
526.9488	279.7	0.23
527.9874	117.7	0.1
528.9772	348.7	0.29
529.7908	119.8	0.1
530.6435	278.1	0.23
531.2295	192.2	0.16
531.9921	150.5	0.12
532.9249	288.4	0.24
533.5741	90.5	0.07
534.373	148.8	0.12

535.1141	216.8	0.18
536.1769	193.6	0.16
537.1246	262.9	0.22
538.1278	190.3	0.16
539.0184	246.6	0.2
539.9017	137.4	0.11
540.8593	310.2	0.26
541.5051	1.3	0
542.0135	149.1	0.12
543.0261	268.9	0.22
543.8889	165	0.14
545.0798	649.3	0.53
545.9074	238.1	0.2
546.6357	260.2	0.21
547.2455	527.6	0.43
548.2237	297.3	0.24
549.055	233.4	0.19
549.8928	121.4	0.1
550.6055	150.5	0.12
551.2244	194.5	0.16
552.1064	123.4	0.1
552.8471	157.4	0.13
553.6573	140.5	0.12
554.5546	148.4	0.12
555.194	204.6	0.17
555.8948	86.4	0.07
556.8299	232.2	0.19
557.6026	79.8	0.07
558.2901	107.7	0.09
559.0525	189.1	0.16
559.8076	111.5	0.09
560.6345	184.3	0.15
561.3213	197.9	0.16
562.2291	145	0.12
563.1449	610.6	0.5
564.1674	287.5	0.24
565.1403	223.8	0.18
565.8266	0.3	0
566.391	206.8	0.17
567.1867	207	0.17
568.0136	150.3	0.12
568.9393	226.5	0.19
569.9251	160.4	0.13
570.5355	2.2	0
571.0632	398.2	0.33
572.0189	172	0.14
572.9456	273.8	0.23
573.9741	153.8	0.13
575.0041	231.2	0.19
575.9088	80.5	0.07
576.9192	294.5	0.24
578.034	160.5	0.13

578.9528	248.4	0.2
579.7592	134.7	0.11
580.5668	128.1	0.11
581.2114	171.2	0.14
581.7713	82	0.07
582.583	167.8	0.14
583.4066	132.3	0.11
584.415	125.1	0.1
585.1538	153.7	0.13
586.0763	151.8	0.12
586.9555	161.7	0.13
587.9981	119.6	0.1
589.0072	185.2	0.15
589.9866	89.8	0.07
590.9866	273	0.22
591.6361	143.2	0.12
592.7035	283.8	0.23
593.5729	122.7	0.1
594.4459	170.3	0.14
595.2314	144.8	0.12
596.2455	172	0.14
597.2712	182.9	0.15
598.268	136.5	0.11
599.1319	151.6	0.12
600.2264	120.1	0.1
601.1421	138.8	0.11
602.0697	79.1	0.07
602.8361	113.7	0.09
603.413	95.7	0.08
604.4039	169.2	0.14
605.2946	147	0.12
606.2402	109.5	0.09
606.9224	140.6	0.12
607.8567	98.1	0.08
608.9334	198.7	0.16
609.8905	114	0.09
610.809	161.3	0.13
611.443	65.5	0.05
611.9993	0.7	0
612.5231	168.9	0.14
613.3619	162.7	0.13
614.2372	103.2	0.08
615.0926	149.6	0.12
615.9824	85.8	0.07
616.9069	124.9	0.1
617.5891	97.8	0.08
618.2996	52.6	0.04
618.9742	138.8	0.11
620.0834	113.3	0.09
621.1116	151.3	0.12
622.0773	110.4	0.09
623.0343	116.5	0.1

623.8256	72	0.06
624.8127	148	0.12
625.8357	114.5	0.09
626.9947	169.9	0.14
627.9989	116.5	0.1
628.9724	154.5	0.13
629.9532	98.6	0.08
631.0474	205.4	0.17
631.9471	74	0.06
632.7166	102.1	0.08
633.3916	127.1	0.1
634.5712	187.6	0.15
635.4085	108.4	0.09
636.2743	108.8	0.09
637.1997	176.7	0.15
638.1897	122.8	0.1
639.0666	164.5	0.14
640.1307	89.1	0.07
641.1194	143.4	0.12
642.1437	121.3	0.1
642.6606	0.1	0
643.2464	138.4	0.11
644.2722	180.1	0.15
645.0113	108.7	0.09
645.7688	94.5	0.08
646.2797	1.5	0
646.7892	153.7	0.13
647.6228	105.3	0.09
648.6445	127.5	0.1
649.5598	144.6	0.12
650.3817	124.8	0.1
651.1326	120	0.1
651.9243	47.3	0.04
652.5066	131.3	0.11
653.201	181.3	0.15
653.9342	159	0.13
654.597	108.5	0.09
655.2689	100.7	0.08
656.1325	111.5	0.09
656.8849	141.7	0.12
657.8505	118.8	0.1
659.0014	146.8	0.12
660.0631	104.5	0.09
661.0375	171.5	0.14
662.1391	208.8	0.17
663.1733	201.7	0.17
663.8172	3	0
664.3818	191.4	0.16
665.3541	153.5	0.13
666.3305	127.8	0.11
667.1767	146.8	0.12
668.1738	93	0.08

669.0306	108.5	0.09
670.1169	124.6	0.1
671.1954	147.1	0.12
672.2189	107.7	0.09
673.095	159	0.13
673.9237	97.8	0.08
674.7299	150.9	0.12
675.5387	137.6	0.11
676.4807	130.1	0.11
677.3931	125.1	0.1
678.3992	93.8	0.08
679.328	133	0.11
680.0194	60.9	0.05
680.8069	128.7	0.11
681.592	97.7	0.08
682.3894	106	0.09
683.1933	115.5	0.1
683.9904	77.4	0.06
684.9228	111	0.09
685.9199	136	0.11
686.9826	145.2	0.12
688.1426	261.4	0.22
688.9852	165.4	0.14
690.1341	555.5	0.46
690.9117	270.7	0.22
691.4744	8.5	0.01
692.0133	275.4	0.23
692.7838	36	0.03
693.3997	140.6	0.12
694.331	79.3	0.07
695.0993	165.8	0.14
695.9581	111.5	0.09
696.7749	128.6	0.11
697.5574	107.3	0.09
698.4267	100.4	0.08
699.1112	108	0.09
700.1745	133.8	0.11
701.0202	126.2	0.1
702.2731	1037.4	0.85
703.2435	580	0.48
704.2248	253.5	0.21
705.1896	193.9	0.16
706.1562	208.5	0.17
706.9944	189.4	0.16
707.723	107.2	0.09
708.492	107.8	0.09
709.2547	94.6	0.08
709.8376	0.8	0
710.3477	120.5	0.1
711.1617	124.9	0.1
712.1183	90.6	0.07
713.1083	172.5	0.14

714.2067	364.2	0.3
715.0402	149.1	0.12
715.7218	5.8	0
716.3252	7202.2	5.93
717.328	3287.6	2.71
718.3663	863.9	0.71
719.309	311.9	0.26
720.1453	133.6	0.11
721.1219	267.5	0.22
722.0285	216.1	0.18
722.876	201.2	0.17
723.4406	1.6	0
723.9515	146.6	0.12
724.9296	137	0.11
725.9053	143.5	0.12
726.7305	135.1	0.11
728.302	1876.7	1.54
729.3484	893.1	0.74
730.2823	1061.1	0.87
731.0942	503.4	0.41
731.9589	272	0.22
732.8717	174.6	0.14
733.768	166.8	0.14
734.463	147.5	0.12
735.256	153.5	0.13
736.2279	215.2	0.18
737.1942	161.9	0.13
737.9781	112.1	0.09
738.7108	117.9	0.1
739.3583	75.8	0.06
740.129	117.2	0.1
740.9381	149.1	0.12
741.6405	2.1	0
742.2546	528.9	0.44
743.1711	246.8	0.2
743.9039	121.4	0.1
744.713	157.3	0.13
745.6423	129.6	0.11
746.325	3.7	0
747.212	829	0.68
748.2296	574.6	0.47
748.9906	325.7	0.27
749.7759	232.3	0.19
750.7868	196.7	0.16
751.8019	193.6	0.16
752.4192	183.4	0.15
753.3572	195.4	0.16
754.3496	207.3	0.17
755.3922	191.6	0.16
756.4	162.2	0.13
757.2543	127.9	0.11
758.199	157.5	0.13

759.066	143.5	0.12
759.9161	138.3	0.11
761.3459	3052.8	2.51
762.3854	2188	1.8
763.4324	754.6	0.62
764.3386	230.4	0.19
764.8711	156.1	0.13
765.7318	178.3	0.15
766.5711	175.6	0.14
767.5622	151.6	0.12
768.3612	160.4	0.13
769.143	101.3	0.08
769.9736	180.6	0.15
770.8333	194.1	0.16
771.8118	154.8	0.13
772.6915	176	0.14
773.5565	103.3	0.08
774.1381	426.7	0.35
774.8432	296.2	0.24
775.6175	314.5	0.26
776.5697	307.9	0.25
777.4446	199.6	0.16
778.4221	267.5	0.22
779.3882	335.9	0.28
780.3638	174.8	0.14
781.3295	161.6	0.13
782.1727	152.2	0.13
783.1054	159.4	0.13
784.2635	662.6	0.55
784.861	1.9	0
785.3801	440.8	0.36
786.2956	189.3	0.16
787.029	167.8	0.14
787.9141	172.1	0.14
788.8028	231.3	0.19
789.6413	153	0.13
790.6175	186.6	0.15
791.563	133.5	0.11
792.5401	132.4	0.11
793.2536	130.1	0.11
794.1246	114.4	0.09
794.916	182.9	0.15
795.9116	289.6	0.24
796.6888	193.1	0.16
797.453	114.6	0.09
798.1744	131.7	0.11
798.7775	133.2	0.11
799.5595	146.2	0.12
800.4395	186	0.15
801.3393	191.5	0.16
802.1526	218.7	0.18
803.03	156.3	0.13

804.104	331.9	0.27
804.6435	206.7	0.17
805.4136	219	0.18
806.3614	179.2	0.15
807.2608	189.5	0.16
808.123	102.1	0.08
808.9812	162.6	0.13
809.5914	63.2	0.05
810.3442	132.5	0.11
811.2321	215.3	0.18
812.0545	166.5	0.14
812.7924	144.1	0.12
813.4899	150	0.12
814.4222	239.9	0.2
815.2542	154.9	0.13
816.1181	147.4	0.12
817.0352	207.4	0.17
818.1813	127.6	0.11
818.8852	165.6	0.14
819.5942	135	0.11
820.4453	194.3	0.16
821.2785	177.3	0.15
822.3415	198.8	0.16
823.3318	153.6	0.13
824.3706	144.9	0.12
825.2521	185.5	0.15
826.133	118.6	0.1
826.7161	111.2	0.09
827.6746	158	0.13
828.432	64.5	0.05
829.0004	222	0.18
829.5106	0.5	0
830.0595	405.6	0.33
830.9	278.8	0.23
831.7568	184.2	0.15
832.7067	183.8	0.15
833.6049	128.6	0.11
834.5061	129.1	0.11
835.1913	123.8	0.1
835.8571	133.4	0.11
836.6752	135.2	0.11
837.4828	136	0.11
838.2141	110	0.09
838.8641	121.9	0.1
839.5651	132.5	0.11
840.408	100.5	0.08
841.0682	113.5	0.09
841.8933	146.5	0.12
842.5236	171.7	0.14
843.471	166.9	0.14
844.3285	143.9	0.12
845.1674	204.1	0.17

845.8609	153.9	0.13
846.8128	295.1	0.24
847.9309	175.3	0.14
848.8949	132.9	0.11
849.7879	161.9	0.13
850.5074	178.3	0.15
851.4469	195.8	0.16
852.2654	240.6	0.2
853.2229	198.4	0.16
854.0783	116.2	0.1
854.9287	139.9	0.12
855.4921	86.1	0.07
856.2887	124.7	0.1
857.2262	177.7	0.15
858.3327	179	0.15
859.2682	128	0.11
860.0884	79.4	0.07
860.7412	107.3	0.09
861.4348	82.1	0.07
862.2253	159.4	0.13
863.2159	184	0.15
864.1189	159.3	0.13
865.1742	168.7	0.14
866.0742	127.6	0.11
867.1261	134.9	0.11
868.0978	168.4	0.14
869.1802	174	0.14
870.1641	158.5	0.13
871.1537	143	0.12
872.2176	169.3	0.14
873.156	174.7	0.14
874.1386	122.8	0.1
875.1606	259.6	0.21
876.1871	155.9	0.13
877.1253	203.5	0.17
878.1825	160.5	0.13
879.2554	257.2	0.21
880.2457	251.2	0.21
881.2632	206.1	0.17
881.9689	154.7	0.13
882.969	251.1	0.21
883.5871	7.5	0.01
884.2261	164.6	0.14
885.0269	172.8	0.14
885.8226	100.9	0.08
886.4682	118	0.1
887.2632	134.5	0.11
887.9802	99.6	0.08
888.5652	82.6	0.07
889.2705	84.8	0.07
890.1328	96.7	0.08
891.049	123.3	0.1

891.8691	108.2	0.09
892.8897	173.5	0.14
893.8597	118.3	0.1
894.8666	165.4	0.14
895.3678	0	0
895.8739	135.4	0.11
896.8157	100.7	0.08
897.5845	143.1	0.12
898.5128	130.4	0.11
899.3653	99.9	0.08
900.2828	102	0.08
901.2212	114.2	0.09
902.0271	100.5	0.08
902.9886	119.9	0.1
903.9392	138.2	0.11
905.0032	124.5	0.1
906.0048	219.9	0.18
906.8552	254	0.21
907.9148	378.7	0.31
908.525	445.1	0.37
909.3125	263.9	0.22
910.2552	222.5	0.18
911.2	173.4	0.14
912.3406	165.5	0.14
913.407	174.2	0.14
914.2839	140.7	0.12
915.188	148.5	0.12
915.9932	93.3	0.08
916.7264	81.6	0.07
917.4676	126.2	0.1
918.2065	93.8	0.08
919.3086	143.5	0.12
920.1043	159.4	0.13
921.1868	330.5	0.27
922.0352	328.1	0.27
922.6588	166.7	0.14
923.5215	195.9	0.16
924.2756	151	0.12
925.0759	132.2	0.11
925.9668	148.4	0.12
927.0615	183.7	0.15
928.1937	118.7	0.1
929.0012	114.5	0.09
929.7979	144	0.12
930.6267	102.7	0.08
931.4555	126.3	0.1
932.3262	91	0.07
933.0844	120	0.1
933.9866	124.2	0.1
934.7369	63	0.05
935.2711	707.9	0.58
936.3203	426.1	0.35

937.2577	3605	2.97
938.3182	1904.6	1.57
938.8321	0.4	0
939.3889	740.5	0.61
940.4635	318.5	0.26
941.3129	182.2	0.15
942.1077	59.1	0.05
942.7339	107.9	0.09
943.511	151.4	0.12
944.3749	106.7	0.09
945.2775	159.9	0.13
946.3272	138.7	0.11
947.2518	118.3	0.1
948.0191	145.3	0.12
948.8712	126.3	0.1
949.666	126.5	0.1
950.4507	27.5	0.02
951.0177	323	0.27
951.5834	204.1	0.17
952.2683	233.8	0.19
953.1001	244.8	0.2
953.782	169.7	0.14
954.5452	137.1	0.11
955.4336	193.8	0.16
956.4118	122.3	0.1
957.3339	142.9	0.12
958.1699	159.6	0.13
958.8621	19.7	0.02
959.369	221.4	0.18
960.2121	148.6	0.12
961.1916	89.4	0.07
962.1808	127.7	0.11
963.0891	140.3	0.12
963.9623	90.3	0.07
964.6279	87.7	0.07
965.3451	1243.8	1.02
966.3236	649.8	0.53
967.2139	439.4	0.36
968.1745	216.6	0.18
969.1253	194.9	0.16
970.0894	180.2	0.15
971.1432	303.8	0.25
972.014	192.2	0.16
972.6248	5.5	0
973.1551	451.8	0.37
974.1347	308	0.25
975.1747	235.9	0.19
976.1067	137.9	0.11
977.2024	815.5	0.67
978.3922	516.1	0.42
979.2903	13031.5	10.73
980.3441	7073.6	5.82

980.8964	1.4	0
981.4166	2990.7	2.46
982.4436	896.7	0.74
983.3001	582.2	0.48
984.2002	310.5	0.26
985.0311	191.6	0.16
985.7126	121.5	0.1
986.5904	186	0.15
987.2966	153.3	0.13
988.0271	133	0.11
988.9481	210.2	0.17
989.8897	180.2	0.15
990.7906	231.6	0.19
991.6436	130.2	0.11
992.2579	5.4	0
993.2184	739.6	0.61
994.341	336.9	0.28
995.2886	1052.5	0.87
996.2736	596	0.49
996.8194	1	0
997.3748	443.8	0.37
998.3409	225.7	0.19
999.1303	150.9	0.12
1000.023	208.8	0.17
1000.907	226.8	0.19
1001.534	215.6	0.18
1002.312	212.7	0.18
1003.225	331.2	0.27
1004.192	146.6	0.12
1005.25	618.8	0.51
1006.187	396	0.33
1007.261	489.8	0.4
1008.281	296	0.24
1009.279	508.1	0.42
1010.253	193.7	0.16
1011.184	981.6	0.81
1011.915	185.7	0.15
1012.486	441.3	0.36
1013.409	443.9	0.37
1014.387	179.9	0.15
1015.239	1175.9	0.97
1016.294	645.3	0.53
1017.254	916.7	0.75
1018.392	417.3	0.34
1019.4	264.9	0.22
1019.967	4.4	0
1020.493	173.4	0.14
1021.422	248.7	0.2
1022.451	320.2	0.26
1023.374	543.7	0.45
1024.366	418.3	0.34
1025.343	1043.3	0.86

1026.309	671.4	0.55
1027.257	560.4	0.46
1028.202	280.3	0.23
1029.101	268	0.22
1029.94	157.3	0.13
1030.722	138.7	0.11
1031.258	260.6	0.21
1032.205	123.3	0.1
1032.891	304.3	0.25
1033.671	173.5	0.14
1034.307	6	0
1034.831	524.3	0.43
1035.66	260	0.21
1036.423	157.3	0.13
1037.249	604.4	0.5
1038.084	268.8	0.22
1038.835	446.6	0.37
1039.366	2754.2	2.27
1039.911	1	0
1040.45	1665.6	1.37
1041.361	2273.3	1.87
1042.373	1271.7	1.05
1043.331	909.8	0.75
1044.43	454.1	0.37
1045.412	519.8	0.43
1046.462	182.7	0.15
1047.232	1203.6	0.99
1048.348	802.2	0.66
1049.433	441.1	0.36
1050.084	13.4	0.01
1050.607	247.6	0.2
1051.259	498.7	0.41
1052.13	289	0.24
1053.241	1568.4	1.29
1054.252	923.4	0.76
1055.23	926.7	0.76
1056.34	552	0.45
1057.323	796.9	0.66
1058.311	414.6	0.34
1059.315	542	0.45
1060.228	379.5	0.31
1061.119	352.8	0.29
1061.8	263.6	0.22
1062.614	234	0.19
1063.325	317.9	0.26
1064.187	395.3	0.33
1065.295	344.8	0.28
1066.326	209.3	0.17
1067.322	1192	0.98
1068.392	524.9	0.43
1069.318	3942.8	3.25
1070.363	2278	1.88

1071.404	1641.2	1.35
1072.474	746.9	0.61
1073.472	560.5	0.46
1074.435	319.8	0.26
1075.33	680.7	0.56
1075.934	0	0
1076.455	558.5	0.46
1077.383	578.1	0.48
1078.344	505.9	0.42
1079.23	391	0.32
1080.077	271.4	0.22
1080.589	1.2	0
1081.113	528.7	0.44
1081.845	274.5	0.23
1082.566	12.8	0.01
1083.267	1673.7	1.38
1084.376	836.2	0.69
1085.316	2031.4	1.67
1086.345	1227.2	1.01
1087.377	1653.4	1.36
1088.327	828.4	0.68
**1089.143**	722.5	0.59
**1089.935**	550.7	0.45
1090.975	703.3	0.58
1092.027	364.5	0.3
1093.152	997.2	0.82
1093.76	1.5	0
1094.274	736.2	0.61
1094.863	0.5	0
1095.365	724.2	0.6
1096.247	165.8	0.14
1097.354	6844	5.63
1097.942	0.2	0
1098.48	4054.9	3.34
1099.43	3368.5	2.77
1100.405	1492.1	1.23
1101.38	2289.5	1.88
1101.937	0.2	0
1102.439	1167.6	0.96
1103.401	1105.6	0.91
1104.478	672.9	0.55
1105.35	1353.3	1.11
1106.298	776.5	0.64
1107.181	945.8	0.78
1108.126	629.4	0.52
1109.281	971.1	0.8
1110.309	557.7	0.46
1111.363	4557.6	3.75
1112.515	3139.4	2.58
1113.468	1574.2	1.3
1114.417	858.1	0.71
1115.29	2655.8	2.19

1116.357	1641.5	1.35
1117.409	1236.7	1.02
1118.549	567.4	0.47
1119.576	675.6	0.56
1120.462	529.2	0.44
1121.377	532.5	0.44
1122.255	503.3	0.41
1123.237	560.5	0.46
1124.205	368.7	0.3
1125.269	967.2	0.8
1126.184	573.5	0.47
1126.833	1	0
1127.393	6271.5	5.16
1128.477	3759	3.09
1129.538	2364.8	1.95
1130.627	1038	0.85
1131.523	793.8	0.65
1132.393	632.2	0.52
1133.341	1274.2	1.05
1134.314	887	0.73
1135.146	1121.5	0.92
1135.877	728.7	0.6
1136.591	14.2	0.01
1137.158	1762.1	1.45
1137.957	734.9	0.6
1138.828	1081.3	0.89
1139.417	3020.1	2.49
1140.538	2388.6	1.97
1141.427	2611.6	2.15
1142.335	1800.1	1.48
1143.309	4994	4.11
1144.378	2932	2.41
1145.432	2057.2	1.69
1146.42	848.5	0.7
1147.338	2499.9	2.06
1148.401	1670.3	1.38
1148.982	0.1	0
1149.559	2042.5	1.68
1150.651	1086.8	0.89
1151.49	815	0.67
1152.366	602.5	0.5
1153.376	1695.9	1.4
1154.337	1095	0.9
1155.316	1870	1.54
1156.397	1286.5	1.06
1157.45	12614.3	10.38
1158.525	7936.1	6.53
1159.519	6995.9	5.76
1160.601	3615.9	2.98
1161.51	2803.8	2.31
1162.445	1439.4	1.18
1163.462	2530.3	2.08

	1164.146	43	0.04	
	1164.717	2513.5	2.07	
	1165.723	1334.5	1.1	
	1166.65	958.3	0.79	
	1167.459	999.7	0.82	
	1168.281	723.1	0.6	
	1169.233	1384	1.14	
	1170.18	956.3	0.79	
	1170.913	3.3	0	
	**1171.432**	**25291.6**	**20.82**	**C57H88O25**
**Assamsaponin A**	1171.972	6.4	0.01	
	1172.536	16675.2	13.73	
	1173.466	21998.9	18.11	
	1174.511	11648.1	9.59	
	1175.54	6068.2	5	
	1176.608	2874.6	2.37	
	1177.518	1714.2	1.41	
	1178.091	5.1	0	
	1178.623	2657.9	2.19	
	1179.657	2329.6	1.92	
	1180.615	3024.5	2.49	
	1181.464	2301.7	1.89	
	1182.345	1080.6	0.89	
	1183.314	2389.8	1.97	
	1184.163	1235.2	1.02	
	1184.838	21.8	0.02	
	1185.473	19668.8	16.19	
**Theasaponin E3**	1186.532	11556.2	9.51	
	**1187.451**	**21833.8**	**17.97**	**C57H88O26**
Theasaponin A_1	1188.567	11521.9	9.48	
	**1189.582**	8004.8	6.59	$C_{57}H_{90}O_{26}$
	1190.682	4325.8	3.56	
	1191.629	2604.7	2.14	
	1192.603	1678.7	1.38	
	1193.476	5602.7	4.61	
	1194.707	6811.7	5.61	
	1195.716	5619.4	4.63	
	1196.819	4377.6	3.6	
	1197.838	2958.8	2.44	
	1199.203	3975	3.27	
	1199.998	1682.2	1.38	
	1200.803	10279.7	8.46	
	1201.558	19820.6	16.32	
	1202.606	13380.3	11.01	
	1203.533	20680.9	17.02	
	1204.59	12603.4	10.38	
	1205.584	8896.5	7.32	
	**1206.637**	4416.8	3.64	
	**1207.451**	6573.1	5.41	
	1208.506	6000	4.94	

	**1209.436**	6297.3	5.18	
	1210.453	4809.9	3.96	
	**1211.494**	**4225.8**	**3.48**	$C_{57}H_{88}O_{26}$
	1212.466	2302.8	1.9	
	1213.447	5845.2	4.81	
	1214.741	5760.1	4.74	
	**1215.567**	24519.1	20.18	$C_{57}H_{88}O_{25}$
	1216.667	17147.6	14.12	$C_{59}H_{92}O_{26}$
	**1217.483**	26898.7	22.14	$C_{58}H_{90}O_{27}$
	1218.551	17596.3	14.49	
Assamsaponin E	1219.541	13788	11.35	
	1220.574	8780.3	7.23	
Theasaponin F_1	1221.673	11124.4	9.16	
	1222.185	0.5	0	
	1222.694	6941	5.71	
	1223.543	11492.8	9.46	
	1224.433	7903.2	6.51	
	1225.239	7152.4	5.89	
	1225.981	4800.8	3.95	
	1226.87	4373.7	3.6	
	1227.67	3592.2	2.96	
	1228.543	2487.5	2.05	
	**1229.526**	121475.3	100	
	**1230.598**	83303.8	68.58	$C_{59}H_{90}O_{27}$
	**1231.55**	75239.1	61.94	$C_{59}H_{92}O_{27}$
	1232.592	37127.5	30.56	
	1233.547	23825	19.61	
	1234.531	12433	10.24	
Theasaponin A_2	1235.412	8247.7	6.79	
	1236.367	7561	6.22	
	1237.46	10388.6	8.55	
	1238.554	9371.8	7.71	
	1239.481	9432	7.76	
	1240.279	6926.2	5.7	
	1241.243	7402.5	6.09	
	1242.215	3948.6	3.25	
	1243.438	17828.2	14.68	
	1244.562	16886.4	13.9	
	1245.321	20171.9	16.61	
	1245.872	16068.9	13.23	
	1246.802	15643.4	12.88	
	1247.57	18680.5	15.38	
	1248.566	15484.1	12.75	
	1249.679	12139.1	9.99	
	1250.298	16	0.01	
	1250.954	12930.1	10.64	
	1251.703	5959.6	4.91	
	1252.582	18176.4	14.96	
	**1253.304**	13125.8	10.81	$C_{59}H_{90}O_{27}$

	1253.983	12754	10.5	$C59H90O27$
	1254.989	15023.3	12.37	
	1255.942	7754.3	6.38	
	1256.809	4293.6	3.53	
	1257.516	11596.8	9.55	
	1258.554	8581	7.06	
	1259.514	63921	52.62	$C_{60}H_{92}O_{28}$
	1260.093	5.7	0	
	1260.606	46970.5	38.67	
	1261.594	43923	36.16	
	1262.684	25294.5	20.82	
	1263.685	16852.4	13.87	
Theasaponins F_2	1264.492	8412.1	6.92	
	1265.404	19955.9	16.43	
	1265.925	3.1	0	
	1266.58	16484.5	13.57	
	1267.64	17029.4	14.02	
	1268.621	15198.7	12.51	
	1269.527	29616.1	24.38	
	1270.595	16463.9	13.55	
	1271.525	47687.1	39.26	**$C61H92O28$**
	1272.611	31629.6	26.04	
	1273.62	34854.6	28.69	$C_{61}H_{94}O_{28}$
	1274.245	2	0	
**Assamsaponin B**	1274.773	20262.2	16.68	
	1275.509	74197.4	61.08	
theasaponin A_3	1276.562	51003.6	41.99	
	1277.591	37993.2	31.28	
	1278.651	20518.2	16.89	
	1279.67	13742.6	11.31	
	1280.831	9903	8.15	
	1281.755	6172.7	5.08	
	1282.74	10188.7	8.39	
	1283.523	7635.5	6.29	
	1284.147	4558.6	3.75	
	1284.991	6026.4	4.96	
	1285.578	16711.2	13.76	
	1286.562	12579.4	10.36	
	1287.458	15050.4	12.39	
	1288.337	8695.6	7.16	
	1289.364	19387.5	15.96	
	1290.355	13210.3	10.87	
	1291.414	15224.7	12.53	
	1291.983	15.9	0.01	
	1292.554	9749.7	8.03	
	1293.516	9221.1	7.59	
	1294.484	7463.3	6.14	
	1295.43	13574.9	11.17	$C61H92O28$
	1296.508	8544.4	7.03	
	1297.463	12131.6	9.99	

1298.465	7522.8	6.19
1299.469	13974.5	11.5
1300.523	9053.4	7.45
1301.498	17743.7	14.61
1302.543	11228	9.24
1303.529	19992.2	16.46
1304.053	4.8	0
1304.623	12379.1	10.19
1305.531	47482.3	39.09
1306.046	3.1	0
1306.622	32578.9	26.82
1307.638	23557.6	19.39
1308.72	13818.7	11.38
1309.632	9622.3	7.92
1310.585	5989.6	4.93
1311.444	6144.8	5.06
1312.466	4327.9	3.56
1313.439	4606.5	3.79
1314.169	2619	2.16
1314.721	9.7	0.01
1315.469	18125.3	14.92
1316.62	11960.9	9.85
1317.522	28272.5	23.27
1318.606	18275.4	15.04
1319.623	18183.2	14.97
1320.667	10449.1	8.6
1321.658	8652.5	7.12
1322.748	6338.8	5.22
1323.636	5331.2	4.39
1324.611	3809.8	3.14
1325.383	3706	3.05
1326.129	2219.6	1.83
1327.046	2534.9	2.09
1327.552	5118.7	4.21
1328.549	4083.7	3.36
1329.47	7066.2	5.82
1330.44	3962.9	3.26
1331.499	11182.4	9.21
1332.638	7578	6.24
1333.593	8037.5	6.62
1334.632	5666.2	4.66
1335.581	5910.4	4.87
1336.494	4395	3.62
1337.515	7177.7	5.91
1338.558	4571.2	3.76
1339.56	6567.8	5.41
1340.618	4655.9	3.83
1341.587	4139.2	3.41
1342.525	1769.4	1.46
1343.462	9501.7	7.82
1344.065	8.9	0.01
1344.576	6475.7	5.33

1345.579	9648.9	7.94
1346.629	5267.6	4.34
1347.491	9271.4	7.63
1348.576	6623	5.45
1349.592	4943.6	4.07
1350.643	2607.3	2.15
1351.509	2960.3	2.44
1352.447	2010.2	1.65
1353.375	3190.7	2.63
1353.942	1691.8	1.39
1354.776	2405.7	1.98
1355.572	2415.1	1.99
1356.403	1340.1	1.1
1357.363	3659.9	3.01
1358.43	2233.3	1.84
1359.414	3322.2	2.73
1360.266	1412.6	1.16
1361.474	6044.3	4.98
1362.066	6.1	0.01
1362.698	3359.7	2.77
1363.684	2732.8	2.25
1364.562	1708.5	1.41
1365.491	2398.3	1.97
1366.651	2121.7	1.75
1367.565	2648.9	2.18
1368.618	1698.5	1.4
1369.485	2393.4	1.97
1370.463	2058.9	1.69
1370.971	0.2	0
1371.494	2514.7	2.07
1372.575	1785.2	1.47
1373.52	5166.5	4.25
1374.599	3837.5	3.16
1375.636	3528.4	2.9
1376.735	2261.3	1.86
1377.712	1583.6	1.3
1378.578	1104.8	0.91
1379.437	1352.3	1.11
1380.344	1157.6	0.95
1381.406	1340.9	1.1
1382.387	1018.4	0.84
1383.458	2487.8	2.05
1384.467	1578.6	1.3
1385.479	4143.1	3.41
1386.712	2854.4	2.35
1387.616	2293.7	1.89
1388.514	1825.8	1.5
1389.472	1930.2	1.59
1390.421	1217.3	1
1391.356	1537	1.27
1392.241	1072.9	0.88
1393.204	1136.4	0.94

1394.153	820.2	0.68
1394.71	1.2	0
1395.274	1091.3	0.9
1395.994	782.9	0.64
1397.003	1338.1	1.1
1397.73	1057.7	0.87
1398.481	740.7	0.61
1399.427	1975.8	1.63
1400.414	1127.8	0.93
1401.061	1213.5	1
1401.722	1452.3	1.2
1402.675	1278	1.05
1403.59	1177.1	0.97
1404.667	891	0.73
1405.574	1167.5	0.96
1406.517	1095.2	0.9
1407.533	1437.8	1.18
1408.524	1036.5	0.85
1409.083	0.4	0
1409.619	1071.3	0.88
1410.604	837.7	0.69
1411.675	1125.8	0.93
1412.715	696.3	0.57
1413.693	1289.7	1.06
1414.224	11.1	0.01
1414.81	1206.5	0.99
1415.652	1324.7	1.09
1416.424	783.6	0.65
1417.283	1054.5	0.87
1417.972	771.8	0.64
1418.836	753.8	0.62
1419.649	813.8	0.67
1420.562	575.5	0.47
1421.169	694.2	0.57
1421.872	805.7	0.66
1422.867	773.7	0.64
1423.763	721.4	0.59
1424.68	575.5	0.47
1425.511	658.5	0.54
1426.402	469.8	0.39
1427.456	867.1	0.71
1428.463	605.6	0.5
1429.476	936.6	0.77
1430.389	639.7	0.53
1431.296	685.1	0.56
1432.029	548.4	0.45
1433.005	796.6	0.66
1433.823	494.8	0.41
1434.614	580.2	0.48
1435.572	804.8	0.66
1436.394	430.3	0.35
1437.362	779.6	0.64

1438.346	706.1	0.58
1439.509	990	0.82
1440.19	310.2	0.26
1440.991	345.1	0.28
1441.738	497.7	0.41
1442.595	394.9	0.33
1443.516	484.3	0.4
1444.2	492.9	0.41
1445.165	682	0.56
1446.029	348	0.29
1446.906	460.7	0.38
1447.596	377.7	0.31
1448.352	316.2	0.26
1449.198	458.8	0.38
1449.978	468.9	0.39
1450.776	376.5	0.31
1451.735	504.9	0.42
1452.667	450	0.37
1453.427	473.8	0.39
1454.37	492.5	0.41
1455.399	355.3	0.29
1456.255	422.6	0.35
1456.995	444.1	0.37
1457.723	323.5	0.27
1458.521	353.5	0.29
1459.395	461.6	0.38
1460.086	357.5	0.29
1460.828	310.2	0.26
1461.646	478	0.39
1462.574	398.9	0.33
1463.691	545.2	0.45
1464.781	395.3	0.33
1465.742	384.7	0.32
1466.636	449.4	0.37
1467.58	610	0.5
1468.629	446.7	0.37
1469.587	425.1	0.35
1470.396	264.9	0.22
1471.148	309.8	0.26
1471.859	304.2	0.25
1472.687	341.2	0.28
1473.568	453.6	0.37
1474.383	267.7	0.22
1475.267	379	0.31
1475.991	255.9	0.21
1476.76	321.6	0.26
1477.587	292	0.24
1478.537	327.6	0.27
1479.607	397.1	0.33
1480.685	335.8	0.28
1481.721	370.1	0.3
1482.71	332.8	0.27

1483.338	260.6	0.21
1484.004	284.3	0.23
1484.777	306.1	0.25
1485.641	404.4	0.33
1486.437	237.8	0.2
1487.286	343.8	0.28
1488.353	291.9	0.24
1489.085	1.7	0
1489.62	409	0.34
1490.665	333.9	0.27
1491.41	319.3	0.26
1492.314	296.9	0.24
1493.241	264.9	0.22
1494.208	292.1	0.24
1495.019	253.5	0.21
1495.833	271.1	0.22
1496.724	261.8	0.22
1497.601	303.4	0.25
1498.62	303.4	0.25
1499.441	243.5	0.2
1499.945	0.2	0

Assamsaponin A

Theasaponin E3

Theasaponin A_1

Assamsaponin E

Theasaponin F_1

Theasaponin E4

Theasaponin E1

Theasaponin A_2

Theasaponins F_2

Assamsaponin B

theasaponin A_3
