# Supplementary material for: Pharmacological Approaches to Attenuate Inflammation and Obesity with Natural Products Formulations by Regulating the Associated Promoting Molecular Signaling Pathways
Source: Biomed Res Int. 2021 Nov 12;2021:2521273. doi: 10.1155/2021/2521273 (PMC8605410; doi:10.1155/2021/2521273)
Supplement: Supplementary 5 — File 5: theasaponin E1 structure elucidation by NMR. [file 2521273.f5.pdf]

Fig. S1 structures of various saponins extracted from green tea seed extract.

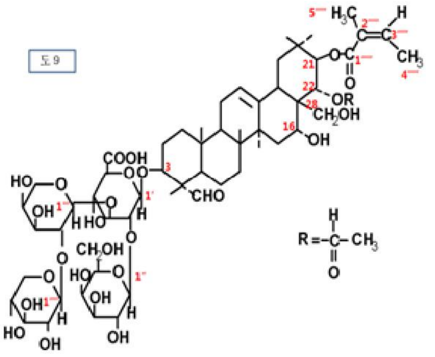

theasaponin E1(GTS E1)

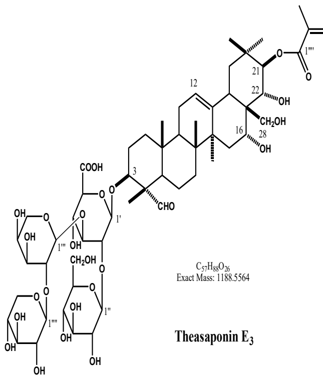

theasaponin E3

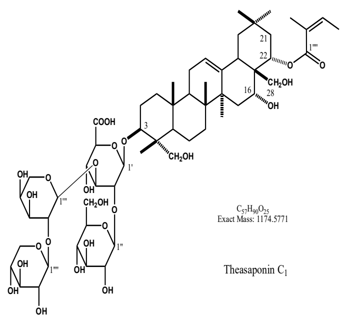

theasaponin C1

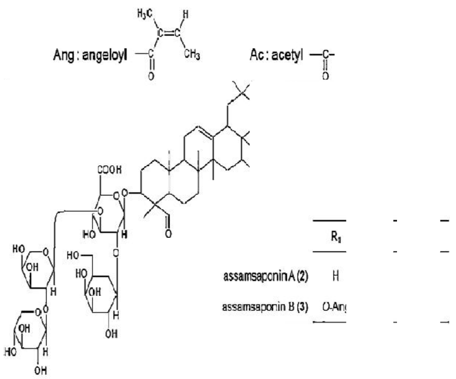

assamsaponin A & B

Table S1. Theasaponin E1 NMR data

**표5.^{13}C-NMR data of 544-4 in pyridine-*d*$_5$.**					
Position	544-4	reference	Position	544-4	reference
1	38.5	38.3	1′	104.4	104.1
2	25.5	25.2	2′	78.6	78.4
3	84.8	84.5	3′	84.1	84.2
4	55.4	55.2	4′	71.2	70.8
5	48.7	48.4	5′	76.9	77.3
6	20.7	20.4	6″	171.3	171.8
7	32.7	32.5	1″	103.6	103.2
8	40.6	40.4	2″	74.0	73.7
9	47.1	46.8	3″	75.7	75.3
10	36.4	36.1	4″	70.8	70.5
11	24.1	23.8	5″	76.8	76.5
12	123.4	123.1	6″	62.4	62.1
13	143.3	142.9	1‴	102.0	101.7
14	42.0	41.8	2‴	82.3	82.3
15	34.9	34.6	3‴	73.8	73.4
16	68.2	68.1	4‴	68.7	68.4
17	48.3	48.0	5‴	66.5	66.1
18	40.4	40.2	1⁗	107.4	107.0
19	47.5	47.2	2⁗	76.3	75.9
20	36.6	36.3	3⁗	78.5	78.2
21	79.2	78.9	4⁗	70.8	70.8
22	74.6	74.5	5⁗	68.0	67.5
23	210.3	209.8	1′′′′′	168.2	167.9
24	11.4	11.0	2′′′′′	129.3	129.0
25	16.1	15.8	3′′′′′	137.5	137.0
26	17.1	16.9	4′′′′′	16.3	15.9
27	27.7	27.4	5′′′′′	21.4	21.0
28	64.1	64.0	1′′′′′′	170.4	171.1

29	29.8	29.5	2''''''	21.2	20.9
30	20.6	20.3			

표6.^{13}C-NMR data of 551G3-1 in pyridine-d_5.

Position	551G3-1	Theasaponin C_1^a	Position	551G3-1	Theasaponin C_1^a
1	39.0	38.7	1′	104.4	104.1
2	25.8	25.5	2′	78.8)	78.5
3	83.3	83.1	3′	84.2	84.6
4	43.8	43.5	4′	70.5	71.0
5	48.4	48.2	5′	77.1	77.4
6	18.4	18.2	6″	172.3	172.0
7	33.1	32.8	1″	103.4	103.2
8	40.4	40.1	2″	74.0	73.8
9	47.3	47.0	3″	75.5	75.3
10	37.0	36.8	4″	70.4	70.1
11	24.1	23.9	5″	76.8	76.5
12	123.4	123.1	6″	62.2	61.9
13	144.0	143.7	1‴	101.9	101.7
14	41.9	41.6	2‴	82.6	82.3
15	35.4	35.2	3‴	73.7	73.4
16	70.4	70.1	4‴	68.6	68.3
17	45.1	44.8	5‴	66.9	66.6
18	41.2	40.9	1⁗	107.3	107.1
19	47.7	47.4	2⁗	76.2	75.9
20	32.3	32.1	3⁗	78.5	78.3
21	42.0	41.7	4⁗	71.1	70.8
22	73.3	73.0	5⁗	67.8	67.5
23	65.1	64.8	1′′′′′	168.3	168.0
24	13.9	13.6	2′′′′′	129.8	129.5
25	16.5	16.2	3′′′′′	136.9	136.6
26	17.2	16.9	4′′′′′	16.2	15.9
27	27.9	27.6	5′′′′′	21.3	21.0
28	63.9	63.6			
29	33.7	33.5			
30	25.5	25.2			

Table S2. TheasaponinC1 NMR data

Table S3. Theasaponin E3 NMR data

**표4.^{13}C-NMR data of 544-3 in pyridine-d_5**

Position	544-3	Theasaponin E_3[a]	Position	544-3	Theasaponin E_3[a]
1	38.5	38.2	1′	104.5	104.2
2	25.6	25.3	2′	78.5	78.3
3	84.4	84.1	3′	84.9	84.5
4	55.5	55.2	4′	71.4	70.8
5	48.7	48.2	5′	77.5	77.3
6	20.7	20.4	6″	171.1	172.0
7	32.7	32.4	1″	103.6	103.3
8	40.6	40.3	2″	74.0	73.7
9	47.1	46.9	3″	75.7	75.4
10	36.4	36.2	4″	70.8	70.4
11	24.1	23.8	5″	76.9	76.5
12	123.4	123.1	6″	62.4	62.1
13	143.1	143.6	1‴	101.9	101.7
14	42.1	41.9	2‴	82.3	82.4
15	34.9	34.4	3‴	73.7	73.4
16	68.2	67.8	4‴	68.6	68.3
17	47.6	47.8	5‴	66.7	66.6
18	40.8	40.5	1⁗	107.4	107.1
19	47.4	47.0	2⁗	75.7	75.9
20	36.3	36.1	3⁗	78.5	78.3
21	81.5	81.7	4⁗	71.1	70.8
22	73.6	73.1	5⁗	67.8	67.5
23	210.4	209.9	1′′′′′	168.9	168.7
24	11.4	11.1	2′′′′′	129.8	129.6
25	16.1	15.8	3′′′′′	136.3	136.0
26	17.3	16.9	4′′′′′	16.2	15.9
27	27.7	27.4	5′′′′′	21.4	21.1
28	66.3	66.0	1′′′′′	64.3	64.5
29	30.1	29.9			
30	20.6	20.4			

Table S4.NMR data ofAssamsaponin A and Assamsaponin B

Assamsaponin A		Assamsaponin B	
δ_C (ppm)	δ_H (ppm, *J*)	δ_C (ppm)	δ_H (ppm, *J*)
37.9	1.53 (m)	37.5	1.53(m)
23.6	1.51 (m)	24.6	1.48 (m)
81.3	3.62 (m)	73.4	3.59 (m)
21.0		22.3	
48.2	1.25 (m)	41.4	1.22 (m)
19.6	1.37 (m)	18.6	1.31 (m)
31.4	1.45 (m)	30.5	1.45 (m)
39.3		35.2	
45.7	1.57(m)	41.7	1.59 (m)
35.1		34.6	
22.7	1.62 (m)	23.8	1.61 (m)
122.4	5.11 (br s)	120.4	5.18 (m)
140.8		140.2	
40.3		40.2	
31.3	1.49 (m)	31.6	1.33 (m)
15.4	0.82 (s)	15.0	0.84 (s)
14.3	0.81 (s)	15.0	0.83 (s)
25.9	1.32 (s)	24.3	1.37 (s)
62.8	3.6 (m),	62.3	3.1 (m)
33.4	1.85 (s)	31.4	1.84 (s)
24.3	2.96 (s)	23.9	2.0 (s)
101.3	4.1(br s)	101.3	4.16 (br s)
73.7	3.41 (m)	73.6	3.46 (m)
59.2	2.51(m)	59.3	3.58 (m)
79.4	2.62(m)	78.2	3.60(m)

65.8	3.61 (m)	62.2	3.65 (m)
171.1		171.2	
101.7	4.31 (m)	101.5	4.30(m)
71.4	3.11 (m)	73.5	3.09(m)
73.5	3.26 (m)	69.7	3.29(m)
68.1	3.45 (m)	68.1	3.42(m)
74.6	3.31(m)	72.3	3.37(m)
59.8	3.56(m)	60.2	3.47 (m)
69.3	3.29 (m)	75.1	3.32 (m)
74.6	3.37 (m)	73.6	3.37 (m)
69.5	3.47 (m)	69.3	3.47 (m)
81.6	3.58 (m)	81.7	3.52 (m)
104.6	4.29 (br s)	104.1	4.32 (d, 7.27)
69.3	3.29 (m)	69.8	3.78(m)
76.4	3.10 (m)	76.7	3.67(m)
73.8	3.00 (m)	73.2	3.45(m)
65.7	3.06 (m)	65.7	3.19 (m)
165.4		165.3	
126.3		126.9	
112.4	5.01 (dq)	112.5	6.08 (dq)
12.3	1.89(m)	12.7	1.89(m)
25.3	1.79(s)	25.3	1.78 (s)
