# Supplementary material for: Pharmacological Approaches to Attenuate Inflammation and Obesity with Natural Products Formulations by Regulating the Associated Promoting Molecular Signaling Pathways
Source: Biomed Res Int. 2021 Nov 12;2021:2521273. doi: 10.1155/2021/2521273 (PMC8605410; doi:10.1155/2021/2521273)
Supplement: Supplementary 6 — File 6: primers sequences used for PCR in this study. [file 2521273.f6.pdf]

Table. S1. Primers list used for RT-PCR analysis in this study

Gene	Primer sequence	Gene bank accession no.	Size (bp)
VEGFR-2	F 5′-AGG TTG CGT GTT CTT CGA GT -3′<br>R 5′-CCC AAA GTG CTG GGT TTT TA -3′	NM_002253.2	934
PI3K	F 5′-CGT GTG CCA TTT GTT TTG AC -3′<br>R 5′-TCA AAC CCT GTT TGC GTT TAC -3′	NM_006218.2	536
VE-Cadherin	F 5′-GGA AGG AGA CAC CAA GCT CA -3′<br>R 5′-CTT GTC ATG CAC CAG TTT GG -3′	NM_001795.3	322
β-Catenin	F 5′-GGT GGG CTG GTA TCT CAG AA -3′<br>R 5′-GGC AAC TGG TAA ACT GTC CAA -3′	NM_001098209.1	629
AKT-1	F 5′-CCG ATT CAC GTA GGG AAA TG -3′<br>R 5′-AGC GTC GAA AAG GTC AAG TG-3′	NM_005163.2	529
NF-kB	F 5′-TGG TCA GCT CCC TTC TCT GT -3′<br>R 5′-GCC AGC TTG GCA ACA GAT-3′	NM_001145138.1	521
ERK	F 5′-TCT GTA GGC TGC ATT CTG GC-3′<br>R 5′-CAG GAC CAG GGG TCA AGA AC-3′	NM_002745.4	528
β-actin (Human)	F 5′-CTC CTG AGC GCA AGT ACT CC -3′<br>R 5′-ACA TCT CAA GTT GGG GGA CA-3′	NM_001101.3	632
PPARγ	F 5′-CTG GCC TCC CTG ATG AAT AA-3′<br>R 5′-GGG TGA AGG CTC ATG TCT GT-3′	NM_001127330.1	393
SREBP	F 5′ -TTG CAC CAG AGA GCA TTT TG-3′<br>R 5′-GAA AAT GAG AGG CTG GTT GC-3′	NM_033218.1	593
CEBPα	F-5′-TTA CAA CAG GCC AGG TTT CC-3′<br>R 5′-CCA CAG GGG TGT GTG TAT GA-3′	NM_007678.3	629
ACC-1	F-5′-ACC TGC CAC AGA AAC CAT TC-3′<br>R-5′-TAT ACA AGC CCA GCC CAC TC -3′	NM_133360.2	397
GPAT	F-5′-ATG TGA GCC CTT TCC TCT CA-3′<br>R-5′-CTC AAA AGC AGC AGG TGT CA-3′	NM_008149.3	299
FAS	F-5′-AAA GGA CCT GCC CAA TCT CT-3′<br>R-5′-TGA TCA AAC TCA GGC TGC AC-3′	NM_007988.3	245
AMPK	F-5′-TGG CTG CCT TCT TAT GCT TT-3′<br>R-5′-GCT TTG AAA CGG CTT CTC AC -3′	NM_178143.2	225
MLYCD	F-5′-GCA GAT CCT CAG CCT GGT AG-3′<br>R-5′-ACA GTG ACA GCC AAG GTG TG-3′	NM_019966.2	205
aP2	F-5′-CAG CCT TTC TCA CCT GGA AG-3′<br>R-5′-TCG ACT TTC CAT CCC ACT TC-3′	NM_024406.2	352
Leptin	F-5′-CTC ATG CCA GCA CTC AAA AA-3′<br>R-5′-AGG TGA CCA AGG TGG CAT AG-3′	NM_008493.3	465
LPL	F-5′-AAG CCC CAC AAG TGT AGT CG-3′<br>R-5′-CGG ACA CAA AGT TAG CAC CA-3′	NM_008509.2	402
β-actin (mouse)	F-5′-GTT GGT TGG AGC AAA CAT CC-3′<br>R-5′-GAG GGT GAG GGA CTT CCT GT-3′	NM_007393.3	151
